# Phosphonium acidic ionic liquid: an efficient and recyclable homogeneous catalyst for the synthesis of 2-arylbenzoxazoles, 2-arylbenzimidazoles, and 2-arylbenzothiazoles†

**DOI:** 10.1039/c8ra01709c

**Published:** 2018-03-27

**Authors:** Quang The Nguyen, Anh-Hung Thi Hang, Thuy-Linh Ho Nguyen, Duy-Khiem Nguyen Chau, Phuong Hoang Tran

**Affiliations:** Department of Organic Chemistry, Faculty of Chemistry, University of Science, Vietnam National University – Ho Chi Minh City 721337 Viet Nam thphuong@hcmus.edu.vn; Center for Innovative Materials and Architectures, Vietnam National University – Ho Chi Minh City 721337 Viet Nam

## Abstract

A highly efficient and green strategy for the synthesis of 2-arylbenzoxazoles, 2-arylbenzimidazoles, and 2-arylbenzothiazoles catalyzed by phosphonium acidic ionic liquid has been developed *via* the condensation of *o*-aminophenol, *o*-phenylenediamines, and *o*-aminothiophenol, respectively, with aldehydes. The reaction has a good yield, the broad substrate scope, and mild condition. Triphenyl(butyl-3-sulphonyl)phosphonium toluenesulfonate catalyst was easily obtained from cheap and available starting materials through a one-pot synthesis. Its structure was identified by ^1^H NMR, ^13^C NMR, ^31^P NMR, and FT-IR techniques. Other properties including thermal stability and acidity were determined by TGA and Hammett acidity function method. Interestingly, the catalyst can maintain its constantly outstanding performance till the fourth recovery.

## Abstract 2

A green, straightforward, and efficient synthesis of 2-arylbenzoxazoles, 2-arylbenzimidazoles, and 2-arylbenzothiazoles catalyzed by phosphonium acidic ionic liquid has been developed.

## Graphical abstract

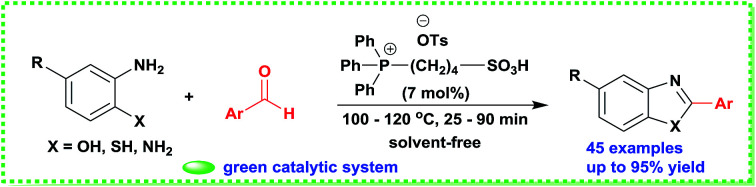

## Introduction

Benzoxazole, benzimidazole, and benzothiazole are well-known heterocyclic scaffolds commonly found in various anticancer, antimicrobial, antidiabetic, anti-inflammatory, antioxidant, anticonvulsant, and analgesic agents due to their wide-spectrum biological and pharmaceutical properties.^1–12^ To date, there have been two pathways for the preparation of these compounds.^13^ The first one relying on the metal-catalyzed cross-coupling is more competent.^14^ However, it has several drawbacks such as expensive catalysts,^15^ addition of auxiliary reagents,^16^ harsh reaction condition,^17^ prolonged reaction time,^18^ low yields,^18^ volatile organic solvents,^19^ which cause a greatly negative impact on the environment as well as an impediment in the scaling up for industrial production. The second synthetic pathway involves the condensation of 2-aminophenol with aldehydes or carboxylic acids under acidic condition.^7,20–24^ Similarly to metal-catalyzed cross-couplings, major defects including the use of expensive chemicals and volatile organic solvents, long reaction times, and tedious work-up procedures have been still unsolvable. Therefore, the development of an efficient, reusable, and green catalyst is eventually favorable to facilitate the large-scale production of these important heterocyclic compounds.

In recent years, ionic liquids (ILs) have received increasing interest as environmentally benign media owing to their special properties such as thermal stability, biodegradability, and non-volatility.^25–29^ They have been widely used as catalysts for a large number of organic transformations.^30–39^ However, their application as catalysts for the synthesis of 2-arylbenzoxazole, 2-arylbenzimidazoles, and 2-arylbenzothiazoles *via* the condensation of aldehydes with *o*-aminophenols, *o*-phenylenediamine, and *o*-aminothiophenols, respectively, has not been known in the literature. In the continuation of our study in ionic liquids application, we reported herein the use of phosphonium acidic ionic liquid as a green and efficient catalyst for the synthesis of the above mentioned arylated heterocycles. The merits of this method are short reaction time, high yield, wide substrate scope, and recyclability of the catalyst.

## Results and discussion

Initially, the condensation of *o*-aminophenol and benzaldehyde is chosen as the model reaction for optimization study. The results were shown in Table 1.

**Table 1:** Optimization data for the synthesis of 2-phenylbenzoxazole

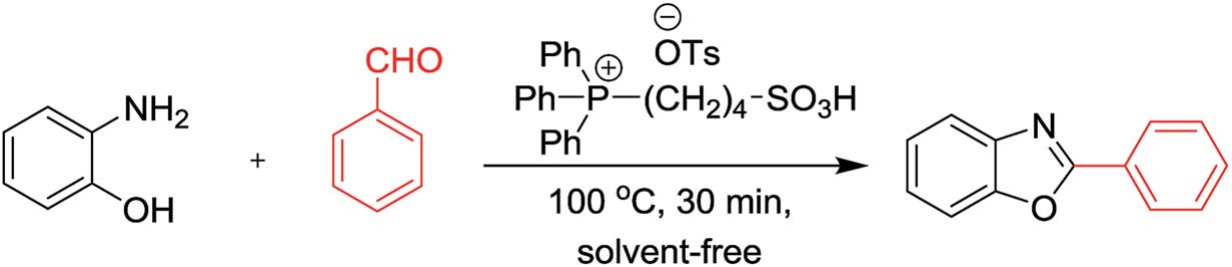				
Entry^a^	Catalyst, (mol%)	Time, (min)	Temperature, (°C)	Yield^b^ (%)
1	10	360	r.t.	Trace
2	10	360	60	47
3	10	90	80	85
4	10	20	100	75
5	10	30	100	92
6	10	40	100	94
7	10	20	120	89
8	7	30	100	91
9	6	30	100	82
10	5	30	100	75
11	0	360	100	—

a Reaction conditions: 2-aminophenol (1 mmol), benzaldehyde (1 mmol), solvent-free.

b Isolated yields by column chromatography (acetone/petroleum ether = 1/19).

The effect of temperature, reaction time, and phosphonium ionic liquid loading was investigated by conducting the reaction at various values of each parameter (r.t. −120 °C, 20–360 min, and 0–10 mol%). It was indicated that best condition at 100 °C, 20 min, and 7 mol% of catalyst could afford the desired 2-phenylbenzoxazole in an excellent yield of 91%. In the absence of the catalyst, the failure in the formation of this product was observed as a result.

**Table 2:** Synthesis of 2-arylbenzoxazoles, 2-arylbenzimidazoles, and 2-arylbenzothiazoles catalyzed by [(C_6_H_5_)_3_P(CH_2_)_4_SO_3_H][OTs]^a^

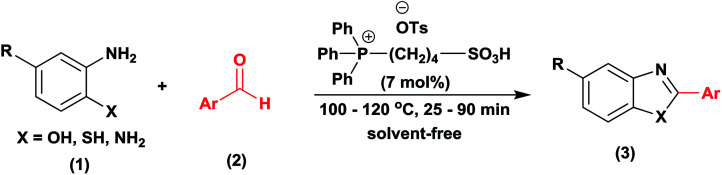					
Entry	_R_R, X, and Ar	Product	Temperature (°C)	Time; (min)	Yield^b^ (%)
1	**1a: R = H, X = OH**, 2a: Ar = Ph	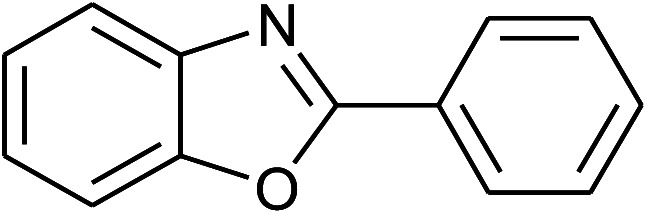	100	30	91
2	2b: Ar = 4-MeC_6_H_4_	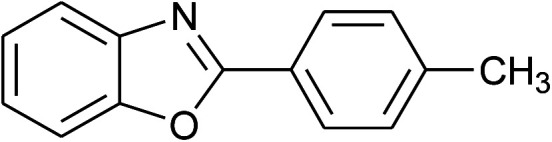	100	45	90
3	2c: Ar = 4-*t*-BuC_6_H_4_	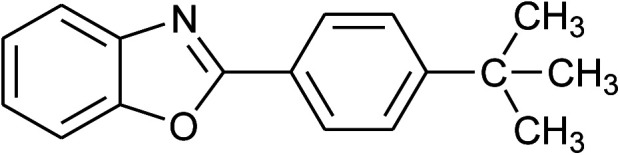	100	45	92
4	2d: Ar = 4-MeOC_6_H_4_	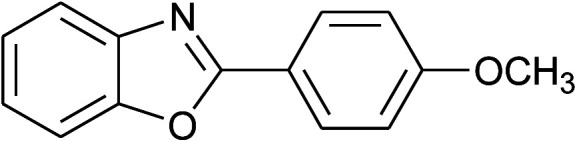	100	50	93
5	2e: Ar = 4-FC_6_H_4_	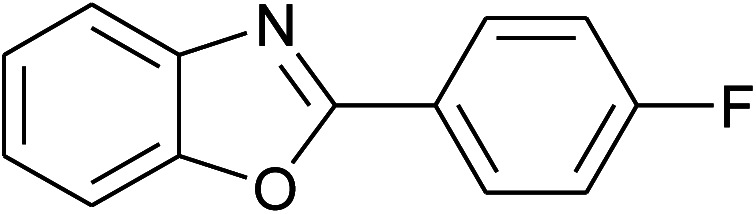	100	45	81
6	2f: Ar = 4-ClC_6_H_4_	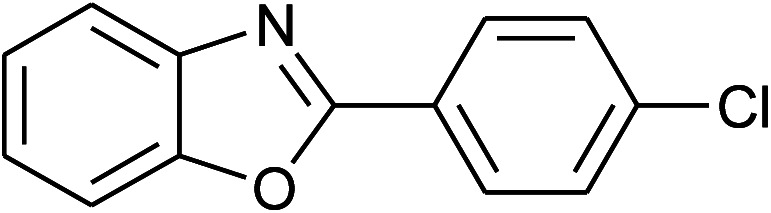	100	45	91
7^c^	2g: Ar = 4-O_2_NC_6_H_4_	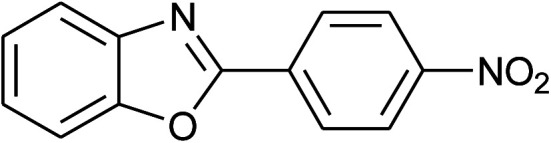	120	50	75
8	2h: Ar = 3-FC_6_H_4_	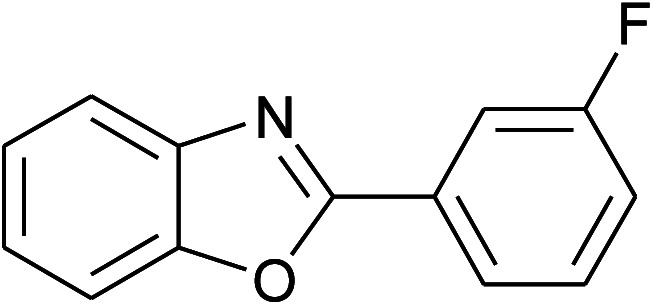	100	40	80
9	2i: Ar = 3-BrC_6_H_4_	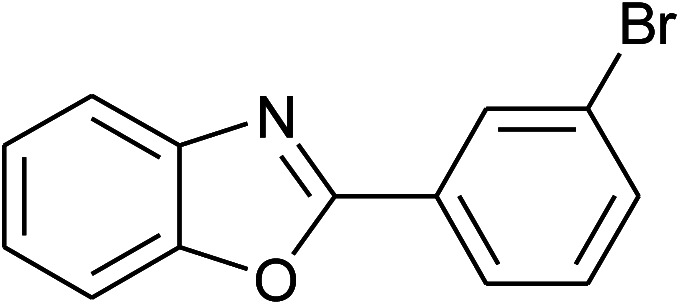	100	45	89
10	2j: Ar = 2-FC_6_H_4_	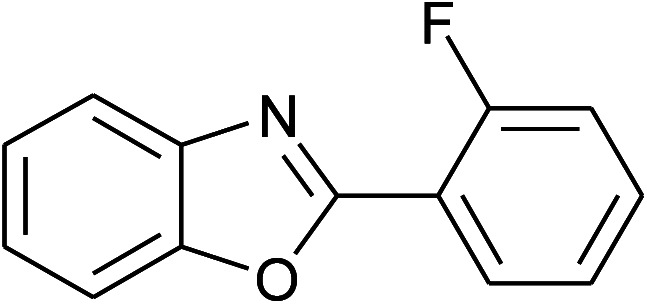	100	45	78
11	2k: Ar = 2-ClC_6_H_4_	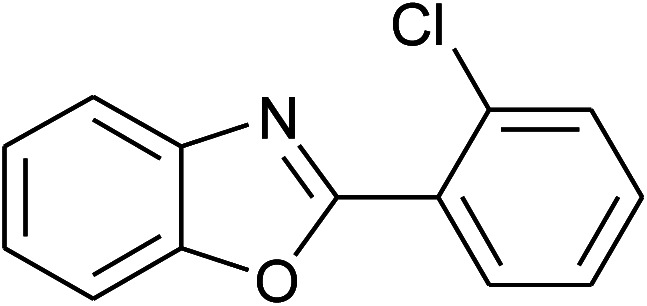	100	45	90
12	2l: Ar = 2-HOC_6_H_4_	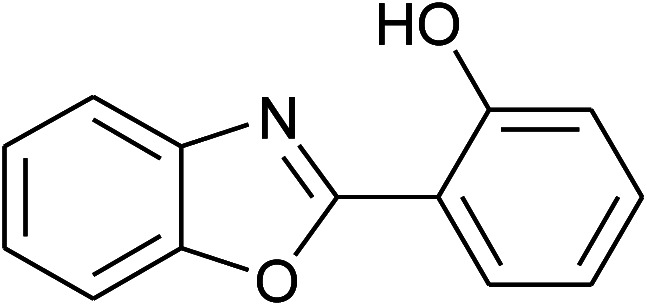	120	50	90
13	2m: Ar = 4-pyridinyl	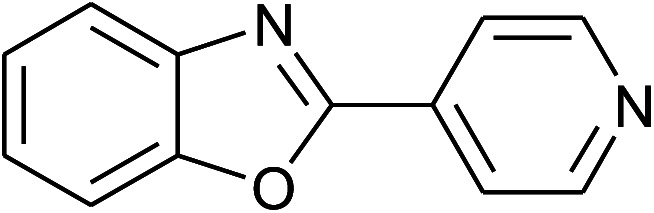	100	50	82
14	**1b: R = Me, X = OH**, 2a: Ar = Ph	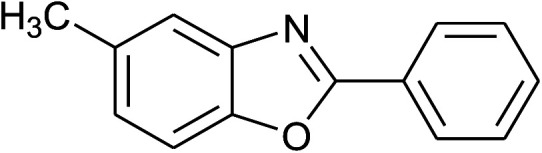	100	35	90
15	2b: Ar = 4-MeC_6_H_4_	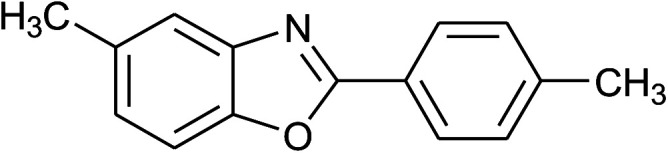	120	40	90
16	2c: Ar = 4-*t*-BuC_6_H_4_	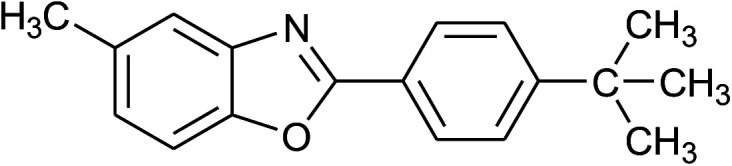	120	45	90
17	2d: Ar = 4-MeOC_6_H_4_	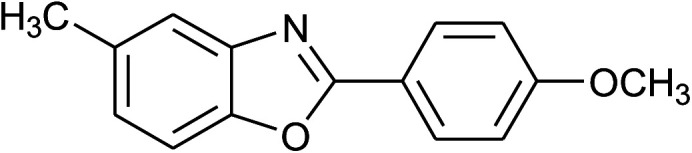	120	50	92
18	2e: Ar = 4-FC_6_H_4_	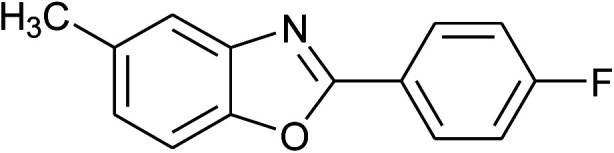	120	45	87
19	2f: Ar = 4-ClC_6_H_4_	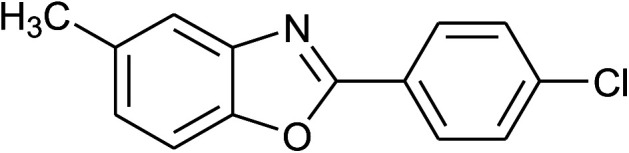	120	45	88
20	2m: Ar = 4-pyridinyl	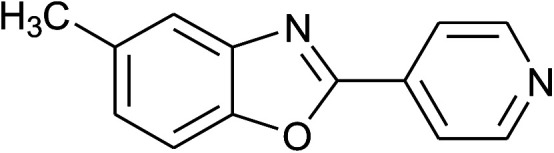	120	50	85
21	2n: Ar = 4-HOC_6_H_4_	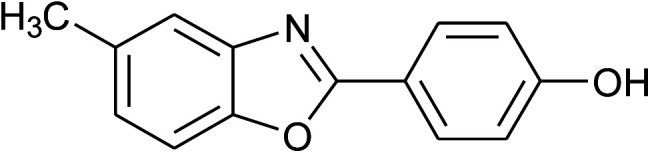	120	60	81
22	**1c: R = Cl, X = OH**, 2a: Ar = Ph	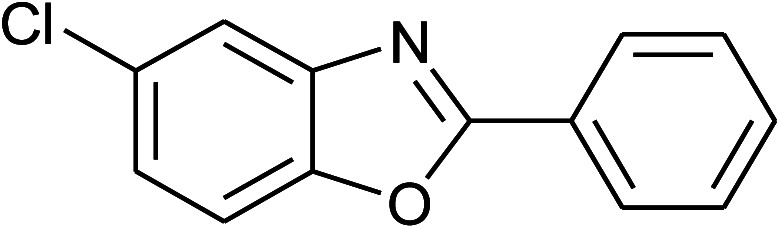	100	25	95
23	2b: Ar = 4-MeC_6_H_4_	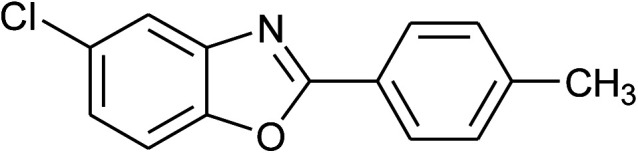	100	45	94
24	2c: Ar = 4-*t*-BuC_6_H_4_	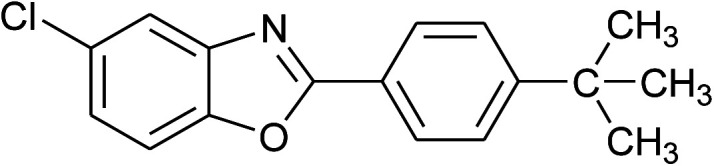	100	50	95
25	2d: Ar = 4-MeOC_6_H_4_	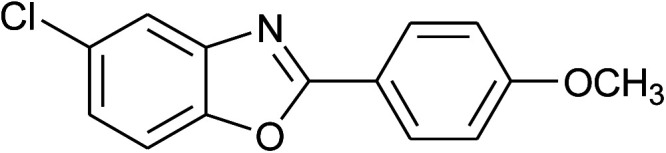	100	50	96
26	2e: Ar = 4-FC_6_H_4_	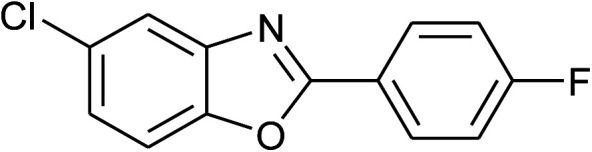	100	45	92
27	2f: Ar = 4-ClC_6_H_4_	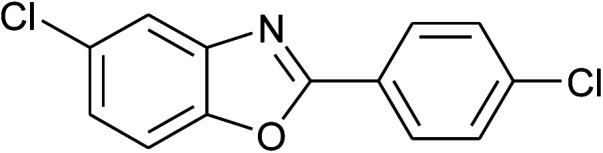	100	45	95
28	2m: Ar = 4-pyridinyl	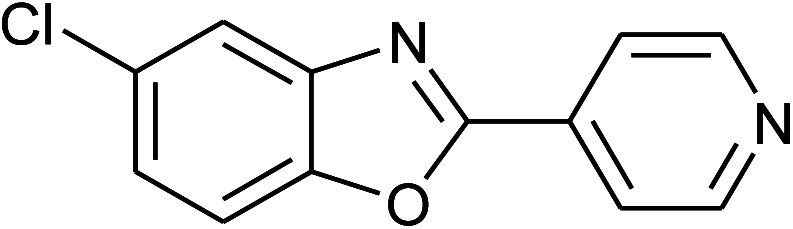	120	45	86
29	2n: Ar = 4-HOC_6_H_4_	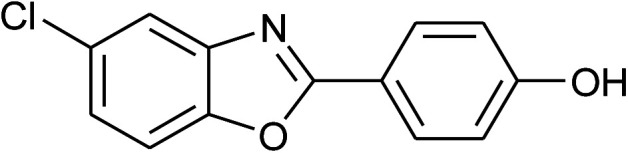	120	50	80
30	**1d: R = NO**_2_, **X = OH**, 2a: Ar = Ph	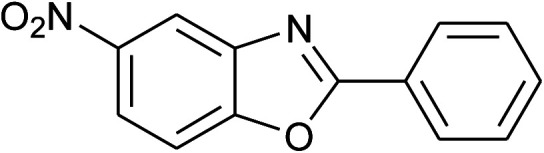	100, 120	75, 50	65, 79^c^
31	2b: Ar = 4-MeC_6_H_4_	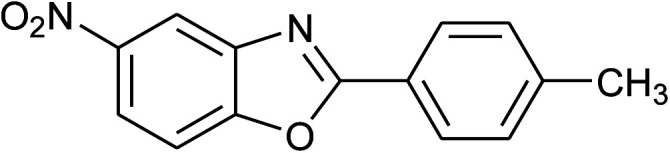	120	50	75
32	2c: Ar = 4-*t*-BuC_6_H_4_	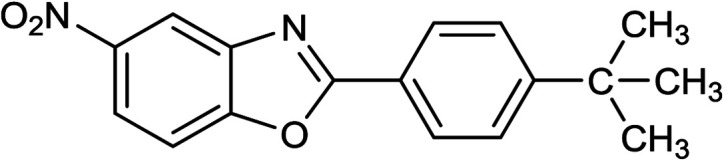	120	55	81
33	2d: Ar = 4-MeOC_6_H_4_	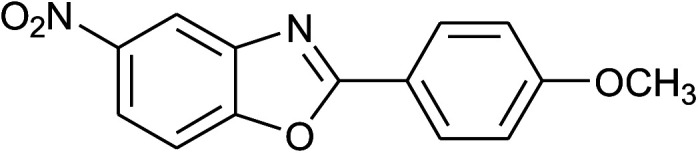	120	50	81
34	2e: Ar = 4-FC_6_H_4_	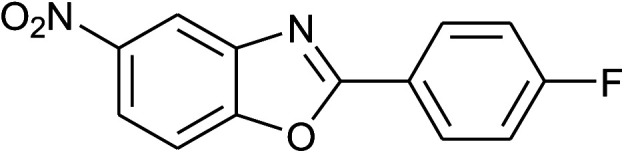	120	60	74
35	2f: Ar = 4-ClC_6_H_4_	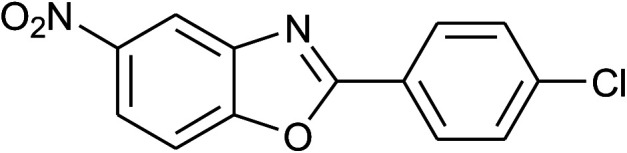	120	60	80
36	**1e: R = H, X = SH**, 2a: Ar = Ph	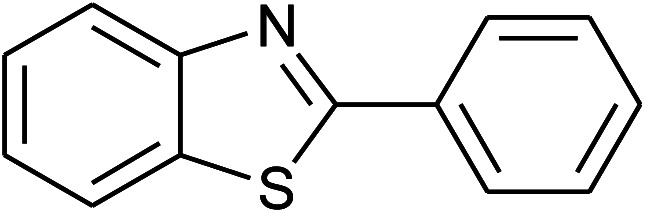	120	50	92
37	2b: Ar = 4-MeC_6_H_4_	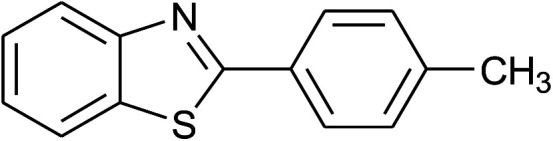	120	60	88
38	2d: Ar = 4-MeOC_6_H_4_	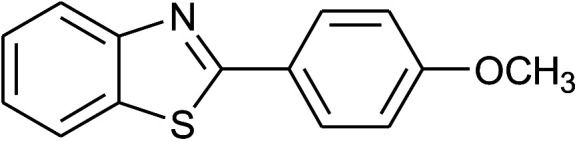	120	60	90
39	2e: Ar = 4-FC_6_H_4_	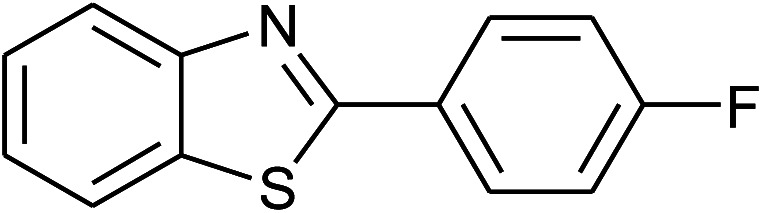	120	60	85
40	2f: Ar = 4-ClC_6_H_4_	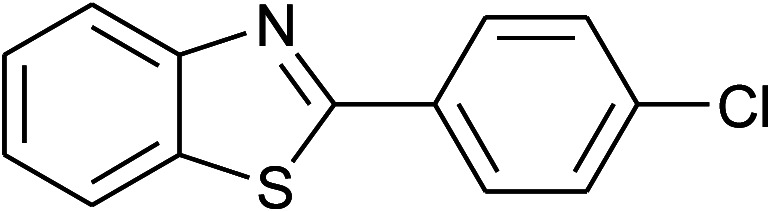	120	60	91
41	2g: Ar = 4-O_2_NC_6_H_4_	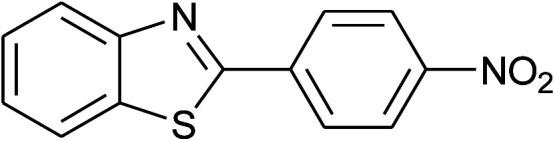	120	70	75^c^
42	**1f: R = H, X = NH**_2_, 2a: Ar = Ph	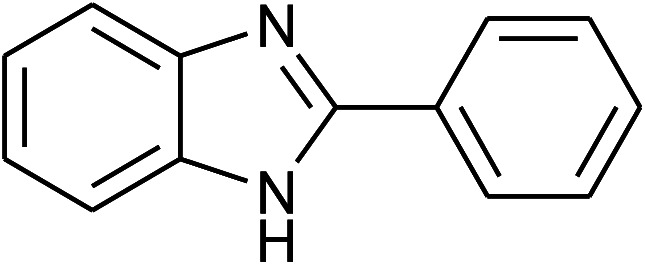	120	90	89
43	2d: Ar = 4-MeOC_6_H_4_	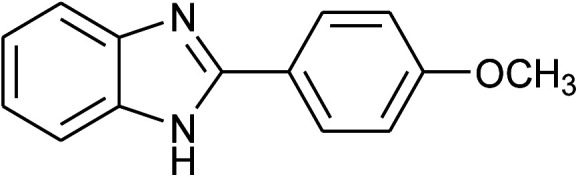	120	90	85
44	2e: Ar = 4-FC_6_H_4_	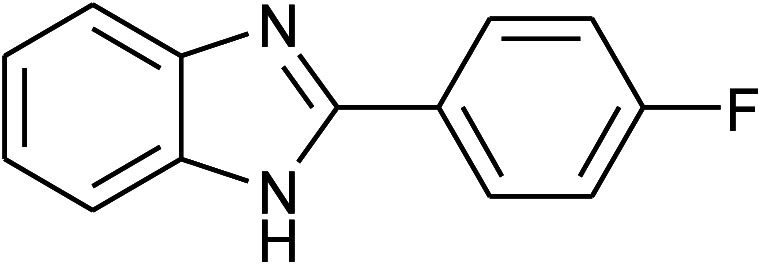	120	90	90
45	2g: Ar = 4-O_2_NC_6_H_4_	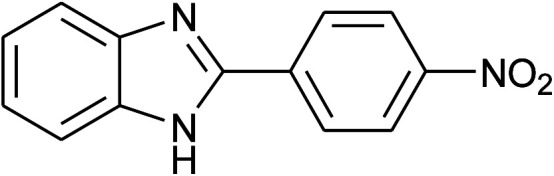	120	90	68^c^

a Reaction conditions: 2-aminophenol (1 mmol), or 2-aminothiophenol (1 mmol), or *o*-phenylenediamine (1 mmol); aldehyde (1 mmol); solvent-free.

b Isolated yields by column chromatography (acetone/petroleum ether = 1/19 or ethyl acetate/hexanes = 1/19).

c Water (0.2 mL) was added to the reaction mixture.

Next, different catalysts were tested for their performance in the synthesis of 2-phenylbenzoxazole (see detailed experiments in the ESI†). The desired product was achieved in the best yield using phosphonium acidic ionic liquid while much lower yields were observed in the same reaction mediated by other catalysts.

With the optimal condition in hand, we explored the reaction scope over a large number of aldehydes in the formation of 2-arylbenzoxazoles and other analogues (Table 2). In general, the reactions proceeded smoothly to give the corresponding products in good to excellent yields. Intuitively, the electronic properties of substituents of benzaldehydes exhibited a little effect on the reaction. As a common trend, electron-rich aldehydes gave the expected products in slightly better yields than electron-poor analogues. Only substrates bearing a powerful electron-withdrawing group such as *p*-nitro or *o*-fluoro can give rise to a dramatic yield decrease as low as 68–78% (entry 7, 10, 41, 45). Similarly, the substituents on 2-aminophenol have certain effect on the yields of the desired products. For 2-aminophenol bearing an electron-donating or a weak electron-withdrawing group such as methyl or chloro, respectively, their condensation with a variety of aldehydes could easily approach the completion with the yields of arylated products above 80%. However, a sharply adverse impact was seen for 2-amino-4-nitrophenol whose arylated products can be only isolated in lower yields of 74–81% compared to 81–93% reported for non-substituted 2-aminophenol (entries 30–35 and 1–6). Meanwhile, on examining the reactivity for a row consisting of eight common aldehydes (entries 14–21 and 22–29), both 2-amino-4-methylphenol and 2-amino-4-chlorophenol can be converted to corresponding heterocyclic adducts in closely similar yields (85–92% for 2-aryl-5-methylbenzoxazoles and 86–96% for 2-aryl-5-chlorobenzoxazoles) compared to those reported in the arylation of non-substituted 2-aminophenol (81–93%). For some arylations in which 2-amino-4-nitrophenol or 4-nitrobenzaldehyde was employed as starting materials, the unexpected solidification of the ongoing reaction mixture can slow down the reaction rate as the result of heterogeneity. Therefore, adding a small amount of DI water (0.2 mL) to the reaction mixture in combination with increasing temperature to 120 °C can be helpful to significantly facilitate the reaction (entries 30, 41, 45).

As our expectation, this synthetic campaign is well-suited for the preparation of not only 2-arylbenzoxazoles but also other heterocyclic analogues including 2-arylbenzimidazoles and 2-arylbenzothiazoles. While 2-aminothiophenol was able to undergo the arylation with the same ease as 2-aminophenol, *o*-phenylenediamine required a prolonged reaction time up to 90 min for a quantitative transformation into expected 2-arylbenzimidazoles. Nevertheless, this harsh condition did not work out in every case. For instance, the arylation of *o*-phenylenediamine by less reactive 4-nitrobenzaldehyde only afforded the desired product in 68% yield even as a small amount of water was added to the reaction mixture to maintain the homogeneity.

**Fig. 1:**
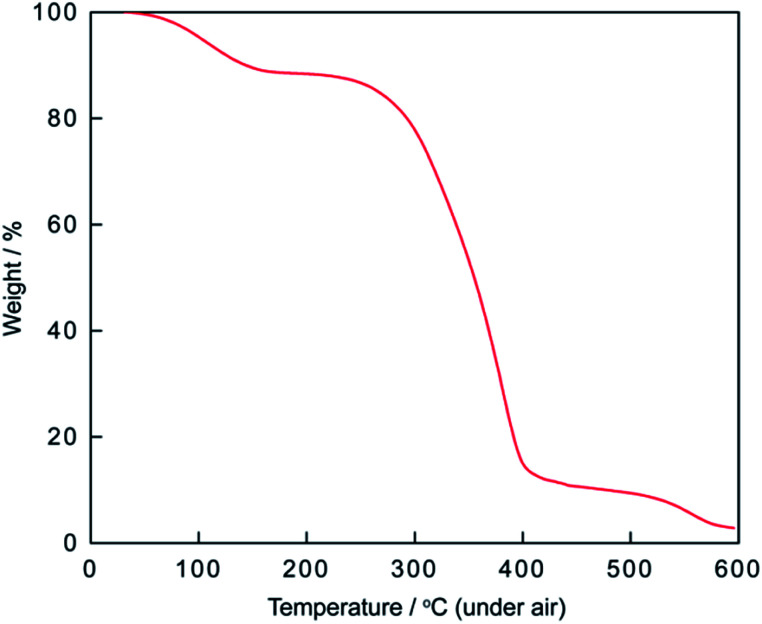
TGA curve of triphenyl(butyl-3-sulphonyl)phosphonium toluenesulfonate.

**Fig. 2:**
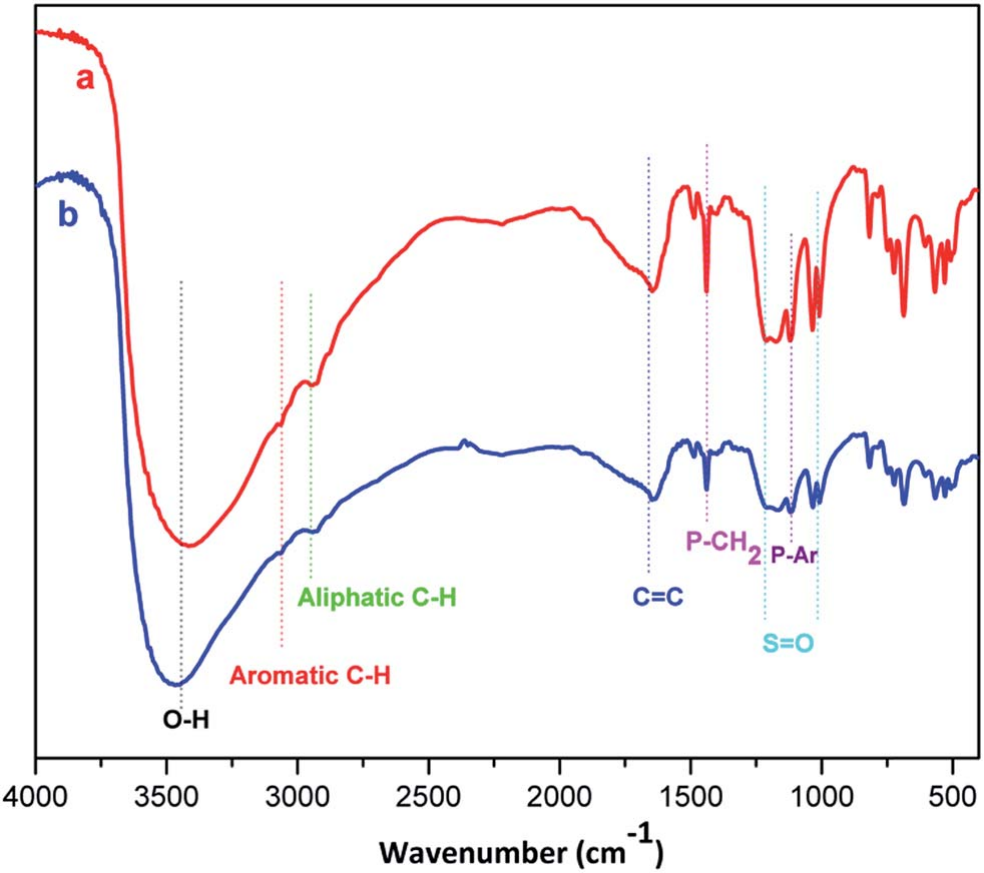
FT-IR spectra of triphenyl(butyl-3-sulphonyl)phosphonium toluenesulfonate (a) and its recycled sample after the fourth run (b).

Phosphonium acidic ionic liquid was readily prepared *via* one-pot two-step procedure in high yield and used as the catalyst for esterification.^40^ However, to the best of our knowledge, no application of this ionic liquid as the catalyst for the condensation between 2-aminophenol, *o*-phenylenediamines or 2-aminothiophenol with aldehydes has been reported in the literature so far. Fig. 1 shows the thermal gravimetric analysis (TGA) of triphenyl(butyl-3-sulphonyl)phosphonium toluenesulfonate. The first weight loss of 11.64% below 200 °C merely corresponds to the loss of residual water in the sample. The phosphonium acidic ionic liquid then maintains its stability up to 220–225 °C before undergoing two consecutive thermal decomposition steps at 225–400 °C and 400–600 °C corresponding to the weight loss of 76.75 and 8.81%, respectively. From the data of TGA as well as latter FT-IR analysis (Fig. 2), it could be firmly assured that there is no structural deformation of the ionic liquid at the temperature range of the investigated arylation of benzoxazole and other analogues (100–120 °C).

The FT-IR spectra of primitively prepared triphenyl(butyl-3-sulphonyl)phosphonium toluenesulfonate and its recovered sample are presented in Fig. 2. The broad peak at approximately 3450 cm^−1^ indicates the presence of hydroxyl group while overlapped shoulder peaks scattered from 2950 to 3100 cm^−1^ are assigned to alkyl and aromatic C–H stretching vibrations. The absorption band at about 1600–1486 cm^−1^ is paired with a number of C=C stretching vibrations in aromatic rings. The signals at 1200 cm^−1^ and 1034 cm^−1^ are asymmetric and symmetric stretching vibrations of S=O bond, respectively. Finally, the presence of phosphonium functional group is confirmed by two concomitant absorption bands at 1121 and 1410 cm^−1^ corresponding with P–Ar and P–CH_2_ bond deformation, respectively. A close similarity in pattern between two FT-IR spectra reinforced the above statement on the thermal stability of phosphonium acidic ionic liquid within the temperature range of arylation.

**Fig. 3:**
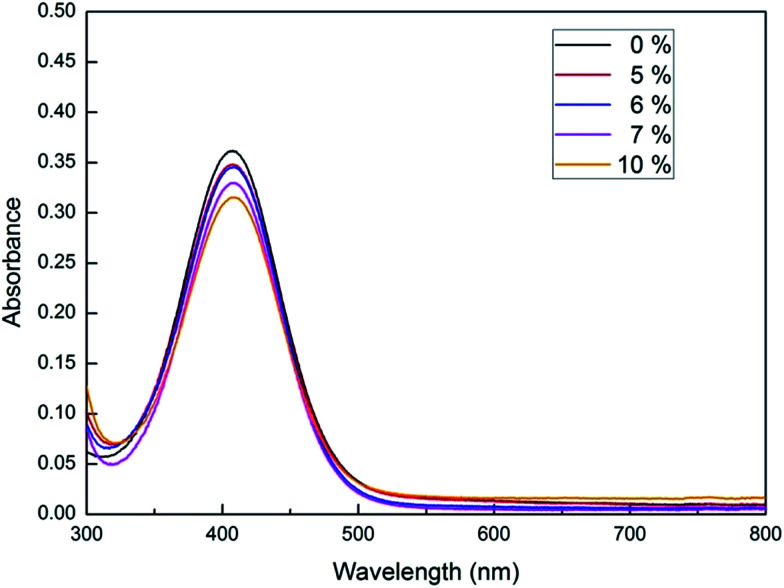
The UV/Vis spectra of 4-nitrodiphenylamine indicator measured in its cosolutions with IL at different concentrations.

The acidity of triphenyl(butyl-3-sulphonyl)phosphonium toluenesulfonate was examined by means of Hammett acidity function method using 4-nitrodiphenylamine as an indicator. *H*_0_ values of ionic liquid solutions with a concentration range of 5–10 mol% were calculated from the [In]/[InH^+^] ratio which was determined by UV-Vis spectroscopy. Technically, this ratio is directly proportional to the absorbance difference of the indicator in its monocomponent solution and its cosolution with a given quantity of ionic liquid. As can be seen from the Table 3 and Fig. 3, the 7 mol% solution of triphenyl(butyl-3-sulphonyl)phosphonium toluenesulfonate has *H*_0_ value of −1.78 claiming that this ionic liquid is about 50 times more acidic than [1,2-DiMIMPs][OTs] analogue with an *H*_0_ value of −0.11 as reported by Yang *et al.*^41^ It could be noted that two analogues almost resemble each other except their cation centers. For the imidazolium-based sulfonic acid ionic liquid, the delocalization of positive charge over imidazole ring gives rise to a lessen acidity of the sulfonic acid moiety. Meanwhile, the sulfonic acid functional group tethered to a localized positive-charged center as triphenylphosphonium can behave with more acidic property owing to a better inductive electron-withdrawing effect of positive charge onto Brønsted sulfonic acid group.

**Table 3:** Hammett acidity function values of various concentrations of investigated IL

Entry	IL (mol%)	*A*_max_	[In] (%)	[InH^+^] (%)	*H*_0_
1	0	0.361	100	0	
2	5	0.347	96.20	3.80	−1.40
3	6	0.345	95.59	4.41	−1.46
4	7	0.329	91.22	8.78	−1.78
5	10	0.315	87.28	12.72	−1.96

**Fig. 4:**
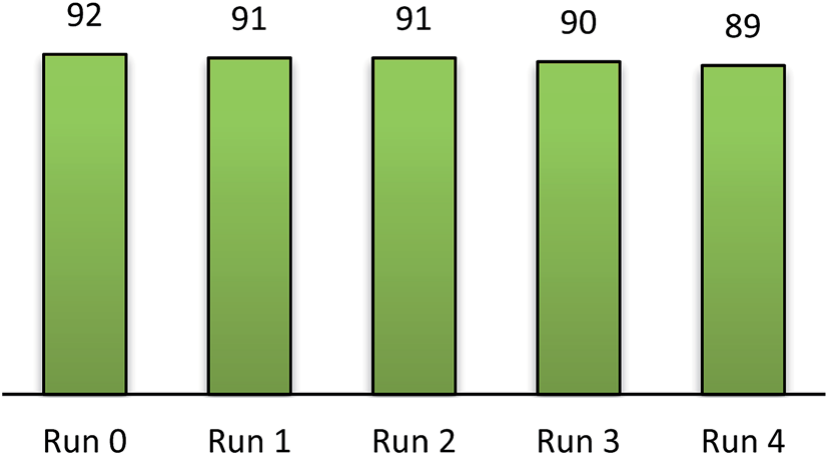
Recycling test of the catalyst.

The recyclability of triphenyl(butyl-3-sulphonyl)phosphonium toluenesulfonate was surveyed on the optimized arylation of 2-aminophenol by benzaldehyde. Upon completion of the reaction, the recovered ionic liquid was easily separated from other organic matters by washing many times with diethyl ether. It was subsequently dried *in vacuo* at 80 °C for 30 min before the reuse for consecutive cycles. It can be seen from Fig. 4 that only a very minor loss of catalytic performance was observed over four times of its reuse in the same condensation of benzaldehyde with 2-aminophenol.

A comparative study of the present method with previous literatures was reported in Table 4. The phosphonium acidic ionic liquid-catalyzed condensation between 2-aminophenol and benzaldehyde afforded the 2-phenylbenzoxazole product in excellent yield under a mild condition without the demand for any additives as in previous reports (Table 4). Furthermore, no loss of catalytic activity in the recycling test of this catalyst is the most prominence of the present method.

**Table 4:** The comparison of the present method with previous literatures in the synthesis of 2-phenylbenzoxazole

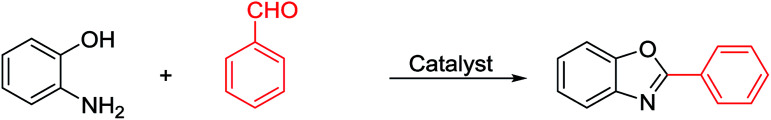			
Entry	Catalyst	Condition	Yield (%)
1	TiO_2_–ZrO_2_ (10 mol%), acetonitrile	60 °C, 15 min	91 (ref. 42)
2	Hf-MOF (1 mol%), solvent-free	140 °C, 6 h	95 (ref. 43)
3	NaCN (10 mol%), DMF, air	80 °C, 24 h	74 (ref. 44)
4	Sm(OTf)_3_ (10 mol%), ethanol–water	50–60 °C, 2 h	92 (ref. 21)
5	Poly(melamine-formaldehyde) (10 mg), oxygen, toluene	110 °C, 24 h	91 (ref. 45)
6	Present work: triphenyl(butyl-3-sulphonyl)phosphonium toluenesulfonate (7 mol%), solvent-free	100 °C, 30 min	91

## Experimental

### Chemicals, supplies, and instruments

All starting materials were purchased from Sigma-Aldrich and employed without further purification. Silica gel (230–400 mesh) for flash chromatography was obtained from HiMedia Laboratories Pvt. Ltd. (India). TLC (silica gel 60 F_254_) was purchased from Merck. Ethyl acetate (purity ≥ 99.5%) and hexanes (≥95%) were obtained from Xilong Chemical Co., Ltd (China). Chloroform-*d* (99.8 atom% D, stab. with Ag) was obtained from Armar (Switzerland). GC-MS spectra were taken on an Agilent GC System 7890 equipped with a mass selective detector Agilent 5973N and a capillary DB-5MS column (30 m × 250 μm × 0.25 μm). FT-IR spectra were recorded in the form of KBr pellets by a Bruker Vertex 70. ^1^H and ^13^C NMR spectra were recorded on a Bruker Advance II 500 MHz.

### Preparation of triphenyl(butyl-3-sulphonyl)phosphonium toluenesulfonate

**Scheme 1:**
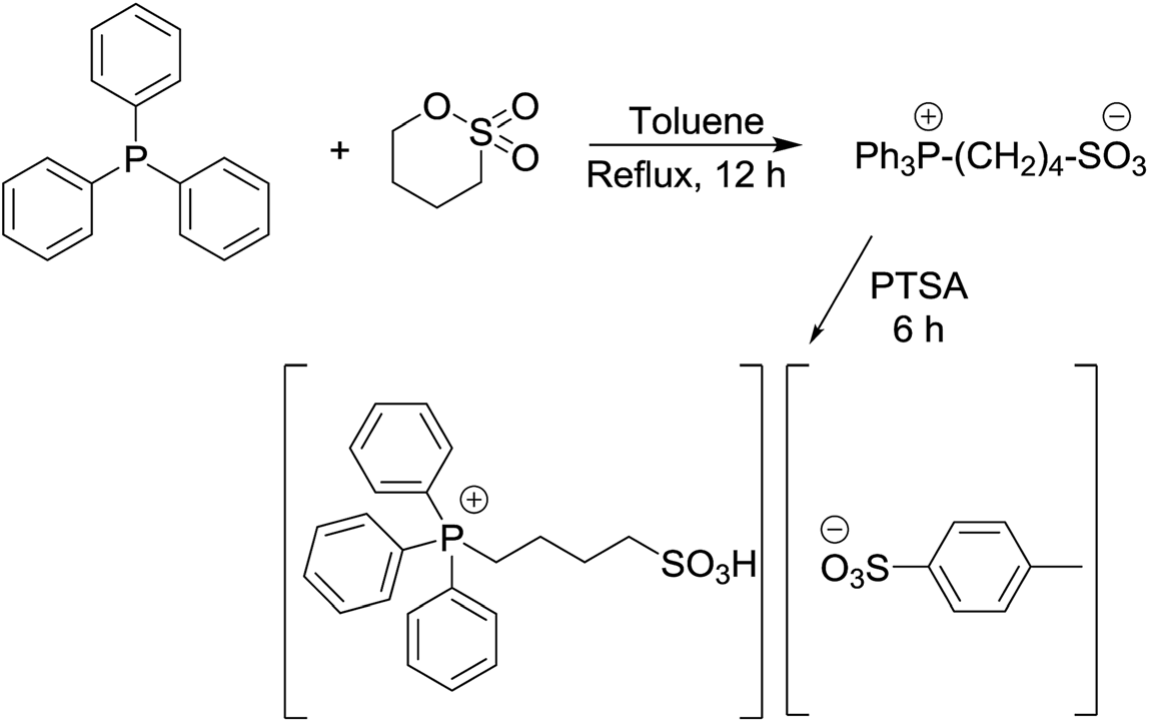
The synthetic pathway of triphenyl(butyl-3-sulphonyl)phosphonium toluenesulfonate.

Triphenyl(butyl-3-sulphonyl)phosphonium toluenesulfonate catalyst was obtained by the one-pot method as depicted in Scheme 1 (97%).^11–14^ Its structure was then authenticated by ^1^H-NMR, ^13^C-NMR, FT-IR and TGA. An equimolar mixture of triphenylphosphine and 1,4-butane sultone in toluene solvent (10 mL) was stirred and heated at reflux for 12 h to obtain the zwitterion as a white solid. Then *p*-toluenesulfonic acid was added dropwise to the resulting zwitterion until a separated clear and colorless liquid layer was formed at the bottom of the round bottom flask after approximately 6 h. After completion of the reaction, the crude product was washed with diethyl ether (5 × 20 mL) to remove non-ionic residues. Finally, the ionic liquid was obtained in 97% yield after the removal of solvent *in vacuo*.

### General procedure for the synthesis of 2-arylbenzoxazole derivatives

2-Aminophenol (109 mg, 1.0 mmol) was treated with benzaldehyde (106 mg, 1.0 mmol) in the presence of triphenyl(butyl-3-sulphonyl)phosphonium toluenesulfonate (20.5 mg, 7 mol%) in a 10 mL glass tube at 100 °C under solvent-free magnetic stirring. Upon completion of the reaction as indicated by TLC after 30 min, the mixture was diluted and extracted with diethyl ether (10 × 5 mL). Then the ethereal solution was washed with water (2 × 20 mL) and dried over Na_2_SO_4_. The final product was obtained after solvent removal by a rotary evaporator followed by the purification on a silica gel column chromatography using acetone/petroleum ether (1/19) as an eluent solvent. The structural characterization was performed using ^1^H, ^13^C-NMR, and GC-MS. The recovered catalyst was reactivated by heating under reduced vacuum at 80 °C for 30 min and reused for next cycles.

## Conclusions

In summary, a green and efficient pathway to access to 2-arylbenzoxazoles, 2-arylbenzimidazoles, and 2-arylbenzothiazoles from *o*-aminophenol, *o*-phenylenediamines, and *o*-aminothiophenol, respectively, *via* the condensation with aldehydes has been successfully developed by outstanding catalytic performance of triphenyl(butyl-3-sulphonyl)phosphonium toluenesulfonate. Not only did this ionic liquid work out on a wide range of substrates and reagents, but it also expressed excellent sustainability of remaining reactivity after four times of recycling. A simple work-up step allowing to isolate the desired products as well as simultaneously recover the ionic liquid catalyst is also an apparent advantage of this process.

## Conflicts of interest

There are no conflicts to declare.

## Supplementary Material

RA-008-C8RA01709C-s001
