# Supplementary material for: Phosphonium acidic ionic liquid: an efficient and recyclable homogeneous catalyst for the synthesis of 2-arylbenzoxazoles, 2-arylbenzimidazoles, and 2-arylbenzothiazoles
Source: RSC Adv. 2018 Mar 27;8(21):11834–42. doi: 10.1039/c8ra01709c (PMC9079114; doi:10.1039/c8ra01709c)
Supplement: RA-008-C8RA01709C-s001 [file RA-008-C8RA01709C-s001.pdf]

Electronic Supplementary Material (ESI) for RSC Advances.
This journal is © The Royal Society of Chemistry 2018

Supporting Information

For

Phosphonium acidic ionic liquid: an efficient and recyclable homogeneous catalyst for the synthesis of 2-arylbenzoxazoles, 2-arylbenzimidazoles, and 2-arylbenzothiazoles

Quang The Nguyen,[a] Anh-Hung Thi Hang,[a] Thuy-Linh Ho Nguyen,[a,b] Duy-Khiem Nguyen Chau,[a] Phuong Hoang Tran[a*]

Department of Organic Chemistry, Faculty of Chemistry, University of Science, Vietnam National University - Ho Chi Minh City, 721337, Viet Nam.

Center for Innovative Materials and Architectures, Vietnam National University – Ho Chi Minh City, 721337, Viet Nam.

*To whom correspondence should be addressed: thphuong@hcmus.edu.vn (Phuong Hoang Tran)

Table of Contents

Section S1. **Materials and analytical techniques** **S2**

Section S2. **Hammett acidity function test** **S2-S3**

Section S3. **Optimization of the catalyst** **S4-S5**

Section S4. **Spectral data** **S6-S29**

Section S5. **^{1}H and ^{13}C NMR spectroscopy** **S30-S75**

Section S5. **References** **S76**

Section S1. Materials and analytical techniques

Chemicals and supplies

1,4-butansultone (≥ 99 %), triphenylphosphophine (≥ 99 %), *p*-toluenesulfonic acid (≥ 99 %), benzaldehyde (≥ 99 %), 4-methylbenzaldehyde (≥ 97 %), 4-*tert*-butylbenzaldehyde (≥ 98 %), 4-methoxybenzaldehyde (≥ 98 %), 4-fluorobenzaldehyde (≥ 98 %), 4-chlorobenzaldehyde (≥ 97 %), 4-bromobenzaldehyde (≥ 99 %), 4-nitrobenzaldehyde (≥ 98 %), 4-pyridinecarboxaldehyde (≥ 97 %), 3-fluorobenzaldehyde (≥ 97 %), 3-bromobenzaldehyde (≥ 97 %), 3-chlorobenzaldehyde (≥ 97 %), 2-fluorobenzaldehyde (≥ 97 %), 2-bromobenzaldehyde (≥ 98 %), 2-aminophenol (≥ 99 %), 2-amino-4-chlorophenol (≥ 97 %), 2-amino-4-methylphenol (≥ 97 %), 2-aminothiolphenol (≥ 99 %), *o*-phenylenediamine (≥ 99.5 %), 4-nitro-*o*-phenylenediamine (≥ 98 %) were obtained from Sigma-Aldrich.

Silica gel 230 – 400 mesh for flash chromatography was obtained from HiMedia Laboratories Pvt. Ltd. (India). TLC (silica gel 60 F_{254}) was purchased from Merck. Ethyl acetate (purity ≥ 99.5 %) and hexanes (≥ 95 %) were obtained from Xilong Chemical Co., Ltd (China). Chloroform-*d*, 99.8 Atom % D, stab. with Ag was obtained from Armar (Switzerland).

All starting materials, reagents, and solvents were used without further purification.

Analytical techniques

GC–MS spectra were carried out on an Agilent GC System 7890 equipped with a mass selective detector Agilent 5973N and a capillary DB–5MS column (30 m x 250 µm x 0.25 µm). FT-IR spectra were recorded from KBr pellets by using a Bruker Vertex 70. ^{1}H and ^{13}C-NMR spectra were investigated on a Bruker Advance II 500 MHz. Hammett acidity function of catalyst was investigated by JASCO V-670 UV-Vis spectrophotometer.

Section S2. Hammett acidity function test

The acidity of various solutions of Brønsted-acidic IL was determined using UV/Vis spectroscopy with 4-nitrodiphenylamine (Sigma-Aldrich, ≥ 98 %) as the indicator. The anhydrous IL was dried by heating *in vacuo* at 90 ^oC in hour in a glove box. Under inert atmosphere, to a series of IL solutions in DI water at given concentrations were added 4-nitrodiphenylamine as the indicator whose concentration in the final solutions is constant at 5.10^{-3} $mol.L^{-1}$. These resulting two-component solutions were shaken for 30 min and then analyzed for the variation in the absorption curves of 4-nitrodiphenylamine at the wavelength region from 350 to 500 nm (**Figure S1**). The experimental data are summarized in **Figure S1** and **Table S1**.

The reported Hammett acidity function values (H_0) were calculated on the basis of the following equation: $H_0 = pK_a(InH^+) + \log([In]/[InH^+])$, wherein $pK_a(InH^+)$ is the pK_a value corresponding to the protonated form of 4-nitrodiphenylamine (-2.38), [In] and $[InH^+]$ are the molar concentrations of the protonated and unprotonated forms of 4-nitrodiphenylamine indicator, respectively.

Based on the UV spectra of **Figure S1**, the quantitative data of H_0 values were obtained in the **Table S1**. The corresponding H_0 values of 5%, 6%, 7%, and 10% IL solutions were -1.40, -1.46, -1.78, and - 1.96, respectively (**Table S1**).

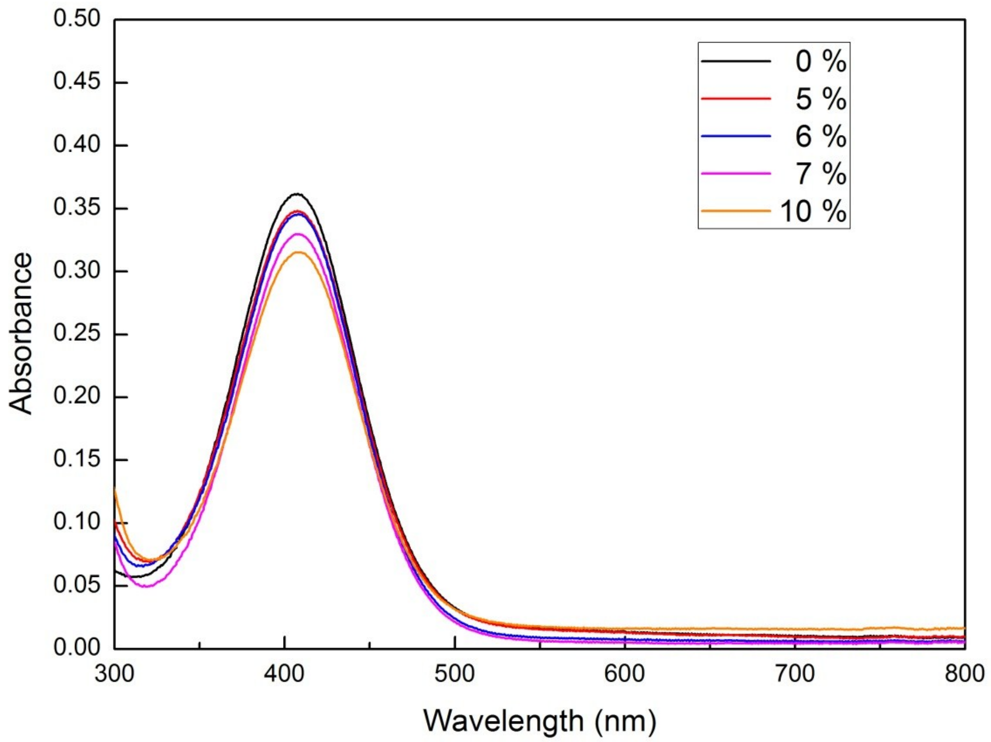

Figure S1. The UV/Vis spectra of 4-nitrodiphenylamine indicator measured in its cosolutions with IL at different concentration.

Table S1. Hammett function values of various concentrations of IL.

**The concentration of catalyst (mol%)**	$\mathbf{A_{max}}$	**[In] (%)**	**[InH$^+$] (%)**	$\mathbf{H_0}$
0	0.361	100	0	
5	0.347	96.20	3.80	-1.40
6	0.345	95.59	4.41	-1.46
7	0.329	91.22	8.78	-1.78
10	0.315	87.28	12.72	-1.96

Section S3. Optimization of the catalyst.

Table S2. The screening of catalyst for the synthesis of 2-phenylbenzoxazole

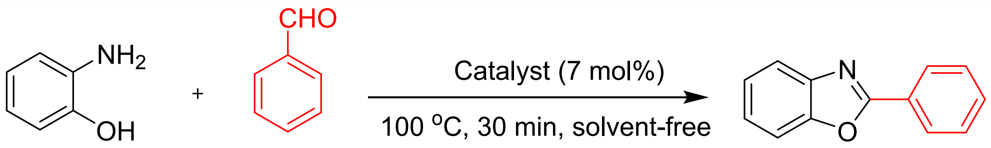

Entry	Type	Catalysts	Yield (%)
1		$CuCl_2$	15
2	Homogeneous salts	$AlCl_3$	18
3		$FeCl_3$	17
4		$FeSO_4$	10
5		Fe_2O_3	7
6		MgO	5
7	Heterogenous oxides	TiO_2	10
8		Al_2O_3	15
9		CuO	5
10		HCl	20
11	Brønsted acids	H_2SO_4	45
12		TsOH	48
13		CH_3COOH	10
14		[MIM]HSO_4	20
15	Brønsted acidic ionic liquids	[1-methylpyrolidine]HSO_4	17
16		[BMIM]$[BF]_6$	5
17	Lewis acidic ionic liquid	[EMIM]$FeCl_4$	10

[a]Reaction conditions: 2-aminophenol (1 mmol), benzaldehyde (1 mmol), catalyst (7 mol%) in solvent-free condition at 100 °C for 30 min.

[b]Yield of pure compound was isolated by column chromatography (acetone/petroleum ether).

Section S4. Spectral data

Triphenyl(butyl-4-sulphonyl)phosphonium toluenesulfonate[1]

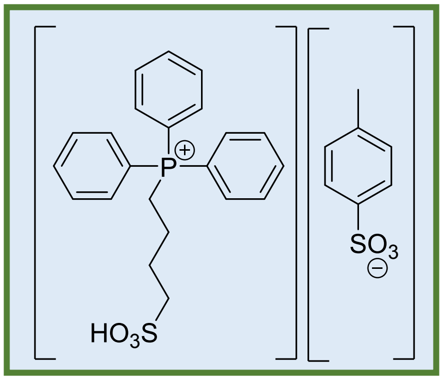

IR (KBr, 4000 – 400 cm^{-1}) 3450, 3075, 2950, 1600, 1486, 1410, 1175, 1121, 1034.

^{1}H NMR (500 MHz, D_2O) δ 7.76 – 7.73 (m, 3H), 7.65 – 7.73 (m, 6H), 7.62 – 7.56 (m, 14), 7.23 (d, J = 8.0 Hz, 3H), 3.33 – 3.17 (m, 2H), 2.78 (t, J = 7.5 Hz, 2H), 2.27 (s, 3H), 1.85 – 1.79 (m, 2H), 1.75 – 1.67 (m, 2H) ppm.

^{13}C NMR (125 MHz, D_2O) δ 142.43, 139.48, 135.02 (d, J = 2.5 Hz), 133.51 (d, J = 10.0), 130.08 (d, J = 12.5 Hz), 129.42, 125.37, 118.33, 49.93, 25.13 (d, J = 17.5 Hz), 21.22 (d, J = 51.2 Hz), 20.73 (d, J = 3.8 Hz), 20.48 ppm.

Characterization of 2-arylbezoxazoles

2-Phenylbenzoxazole[2-5]

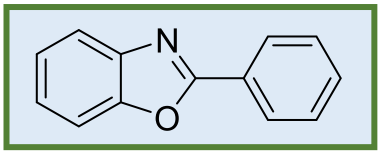

Analytical TLC on silica gel, 1/19 acetone/petroleum ether.

White solid, mp = 102-103 °C.

FT-IR (KBr, 4000 – 400 cm^{-1}): 3059, 2925, 2854, 1775, 1615, 1551, 1475, 1448, 1285, 1240.

^{1}H NMR (500 MHz, $CDCl_3$): δ 8.31 – 8.22 (m, 2H), 7.82 – 7.75 (m, 1H), 7.61 – 7.56 (m, 1H), 7.55 – 7.50 (m, 3H), 7.40 – 7.33 (m, 2H) ppm.

^{13}C NMR (125 MHz, $CDCl_3$) δ 163.2, 150.9, 142.2, 131.7, 129.0, 127.8, 127.3, 125.3, 124.7, 120.1, 110.7 ppm.

GC-MS (EI, 70 eV) *m/z*: 195 ($[M]^+$).

2-(4-Methylphenyl)benzoxazole[5]

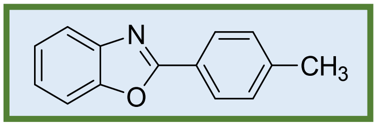

Analytical TLC on silica gel, 1/19 acetone/petroleum ether.

White solid, mp = 113-114 °C.

FT-IR (KBr, 4000 – 400 cm^{-1}): 3056, 2920, 2854, 1728, 1620, 1554, 1499, 1450, 1242.

^{1}H NMR (500 MHz, $CDCl_3$) δ 8.16 – 8.14 (m, 2H), 7.77 – 7.74 (m, 1H), 7.58 – 7.55 (m, 1H), 7.36 – 7.32 (m, 4H), 2.44 (s, 3H) ppm.

^{13}C NMR (125 MHz, $CDCl_3$) δ 163.5, 150.8, 142.3, 142.2, 129.8, 127.8, 125.1, 124.7, 124.5, 120.0, 110.6, 21.8 ppm.

GC-MS (EI, 70 eV) *m/z*: 209 ($[M]^+$).

2-(4-*Tert*-butylphenyl)benzoxazole[3]

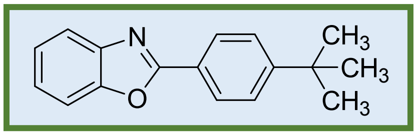

Analytical TLC on silica gel, 1/19 acetone/petroleum ether.

White solid, mp = 107-108 °C.

FT-IR (KBr, 4000 – 400 cm^{-1}): 3059, 2927, 1728, 1547, 1452, 1429, 1287, 1239.

^{1}H NMR (500 MHz, $CDCl_3$) δ 8.20 – 8.18 (m, 2H), 7.75 – 7.78 (m, 1H), 7.59 – 7.57 (m, 1H), 7.56 – 7.54 (m, 2H), 7.36 – 7.32 (m, 2H), 1.38 (s, 9H) ppm.

^{13}C NMR (125 MHz, $CDCl_3$) δ 163.4, 155.4, 150.8, 142.1, 127.7, 126.1, 125.1, 124.7, 124.4, 120.0, 110.7, 35.2, 31.3 ppm.

GC-MS (EI, 70 eV) *m/z*: 251 ($[M]^+$).

2-(4-Methoxyphenyl)benzoxazole[5]

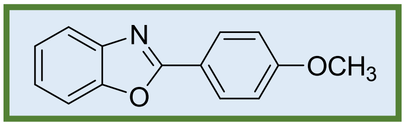

Analytical TLC on silica gel, 1/19 acetone/petroleum ether.

White solid, mp = 103-104 °C.

FT-IR (KBr, 4000 – 400 cm^{-1}): 3050, 2924, 2849, 1615, 1501, 1450, 1420, 1244.

^{1}H NMR (500 MHz, $CDCl_3$) δ 8.22 – 8.18 (m, 2H), 7.75 – 7.73 (m, 1H), 7.56 – 7.54 (m, 1H), 7.35 – 7.30 (m, 2H), 7.04 – 7.01 (m, 2H), 3.89 (s, 3H) ppm.

^{13}C NMR (125 MHz, $CDCl_3$) δ 163.3, 162.6, 150.8, 142.1, 129.7, 124.8, 124.7, 119.7, 119.7, 114.6, 110.6, 55.6 ppm.

GC-MS (EI, 70 eV) *m/z*: 225 ($[M]^+$).

2-(4-Nitrophenyl)benzoxazole[5]

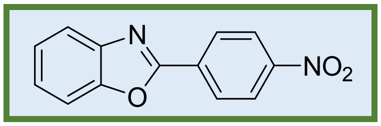

Analytical TLC on silica gel, 1/19 acetone/petroleum ether.

Yellow solid, mp = 256 - 257 ^{o}C.

FT-IR (KBr, 4000 – 400 cm^{-1}): 2925, 2854, 1678, 1610, 1534, 1449, 1237.

^{1}H NMR (500 MHz, $CDCl_3$) δ 8.15 (dd, *J* = 8.0, 1.0 Hz, 1H), 7.89 (dd, *J* = 8.0, 1.0 Hz, 1H), 7.83 – 7.80 (m, 1H), 7.74 (td, *J* = 8.0, 1.0 Hz, 1H), 7.69 (td, *J* = 8.0, 1.0 Hz, 1H), 7.59 – 7.57 (m, 1H), 7.42 – 7.37 (m, 2H) ppm.

^{13}C NMR (125 MHz, $CDCl_3$) δ 158.9, 151.2, 141.7, 132.4, 132.0, 131.6, 126.2, 125.1, 124.3, 120.9, 111.1 ppm.

GC-MS (EI, 70 eV) *m/z*: 240 ([M]$^{+}$).

2-(4-Fluorophenyl)benzoxazole[3]

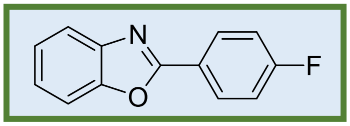

Analytical TLC on silica gel, 1/19 acetone/petroleum ether.

White solid, mp = 99 - 99.5 ^{o}C.

FT-IR (KBr, 4000 – 400 cm^{-1}): 3061, 2925, 1619, 1584, 1582, 1542, 1473, 1448, 1247, 1225.

^{1}H NMR (500 MHz, $CDCl_3$) δ 8.27 – 8.23 (m, 2H), 7.78 – 7.74 (m, 1H), 7.58 – 7.55 (m, 1H), 7.37 – 7.33 (m, 2H), 7.23 – 7.18 (m, 2H) ppm.

^{13}C NMR (125 MHz, $CDCl_3$) δ δ 165.0 (d, *J* = 251 Hz), 162.3, 150.9, 142.2, 130.0 (d, *J*=8.8 Hz), 130.0 (d, *J* = 255.1 Hz) 125.0 (d, *J* = 59.5 Hz), 123.7 (d, *J*=3.3 Hz), 120.1, 116.3 (d, *J*=22 Hz), 110.7 ppm.

GC-MS (EI, 70 eV) *m/z*: 213 ([M]$^{+}$)

2-(4-Chlorophenyl)benzoxazole[5]

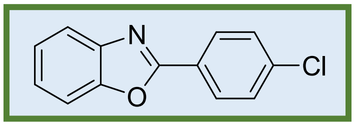

Analytical TLC on silica gel, 1/19 acetone/petroleum ether.

White solid, mp = 146 - 147.5 ^{o}C.

FT-IR (KBr, 4000 – 400 cm^{-1}): 3061, 2925, 1610, 1584, 1582, 1542, 1473, 1448, 1247, 1225.

^{1}H NMR (500 MHz, acetone-d_6) δ 8.26 – 8.23 (m, 2H), 8.27 – 8.22 (m, 2H), 7.77 (m, 1H), 7.71–7.69 (m, 1H), 7.65 – 7.63 (m, 2H), 7.46 –7.39 (m, 2H) ppm.

^{13}C NMR (125 MHz, acetone-d_6) δ 161.7, 150.8, 142.1, 137.2, 129.3, 129.0, 125.9, 125.59, 124.8, 120.0, 110.7 ppm.

GC-MS (EI, 70 eV) *m/z*: 229 ([M]$^{+}$).

2-(3-Fluorophenyl)benzoxazole[3]

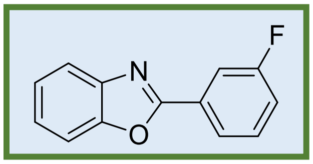

Analytical TLC on silica gel, 1/19 acetone/petroleum ether.

White solid, mp = 99-100 ^{o}C.

FT-IR (KBr, 4000 – 400 cm^{-1}): 3073, 2925, 2855, 1591, 1552, 1480, 1448, 1341, 1269, 1242.

^{1}H NMR (500 MHz, $CDCl_3$): δ 8.05 – 8.04 (m, 1H), 7.96 – 7.94 (m, 1H), 7.79 – 7.77 (m, 1H), 7.59-7.58 (m, 1H), 7.51-7.47 (m, 1H), 7.38 – 7.36 (m, 2H), 7.25 – 7.21 (m, 1H) ppm.

^{13}C NMR (125 MHz, $CDCl_3$) δ 163.0 (d, J = 245.5 Hz), 150.9, 142.1, 130.8 (d, J = 8.1 Hz), 129.3 (d, J = 8.5 Hz), 125.6, 124.9, 123.5 (d, J = 3 Hz), 120.4, 118.7, 118.6 (d, J = 21.3 Hz),114.7 (d, J= 23.9 Hz), 110.8 ppm.

GC-MS (EI, 70 eV) *m/z*: 213 ([M]$^{+}$)

2-(3-Bromophenyl)benzoxazole[4]

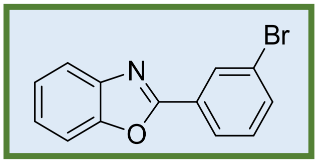

Analytical TLC on silica gel, 1/19 acetone/petroleum ether.

White solid, mp = 129-130 ^oC.

FT-IR (KBr, 4000 – 400 cm^{-1}): 3423, 3055, 2926, 1613, 1570, 1545, 1451, 1426, 1287, 1240.

^{1}H NMR: (500 MHz, $CDCl_3$): δ 8.42 (t, J = 1.5 Hz, 1H), 8.20 – 8.18 (m, 1H), 7.79 – 7.77(m, 1H), 7.67 (m, 1H), 7.66 – 7.65 (m, 1H), 7.42 – 7.37 (m, 3H) ppm.

^{13}C NMR (125 MHz, $CDCl_3$)**:** δ 161.7, 151.0, 142.0, 134.6, 130.7, 130.6, 129.2, 126.3, 125.7, 125.0, 123.2, 120.4, 110.9 ppm.

GC-MS (EI, 70 eV) *m/z*: 273 ($[M]^+$)

2-(2-Fluorophenyl)benzoxazole[6]

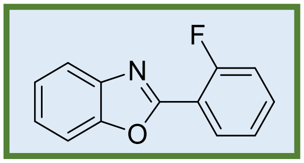

Analytical TLC on silica gel, 1/19 acetone/petroleum ether.

White solid, mp = 93-95 ^oC.

FT-IR (KBr, 4000 – 400 cm^{-1}): 3061, 2925, 1719, 1584, 1582, 1542, 1473, 1448, 1247, 1225.

^{1}H NMR (500 MHz, $CDCl_3$) δ 8.24 (td, J = 7.5, 2.0 Hz, 1H), 7.85 – 7.81(m, 1H), 7.63 – 7.59 (m, 1H), 7.54 – 7.49 (m, 1H), 7.41 – 7.36 (m, 2H), 7.32 – 7.25 (m, 2H) ppm.

^{13}C NMR (125 MHz, $CDCl_3$)**:** δ 162.0 (s), 156.0(s), 150.7 (s), 141.9 (s), 133.25 (d, J = 8.6 Hz), 130.7 (d, J = 1.1 Hz), 125.6 (s), 124.8 (s), 124.63 (d, J = 3.8 Hz), 120.52 (s), 117.32 (d, J = 21.3 Hz), 115.7 (d, J = 10.4 Hz), 110.8 (s) ppm.

GC-MS (EI, 70 eV) *m/z*: 213 ($[M]^+$)

2-(2-Chlorophenyl)benzoxazole[7]

Analytical TLC on silica gel, 1/19 acetone/petroleum ether.

White solid, mp = 100-102 °C.

FT-IR (KBr, 4000 – 400 cm^{-1}): 2925, 1608, 1584, 1569, 1533, 1470, 1452, 1343, 1237, 1185.

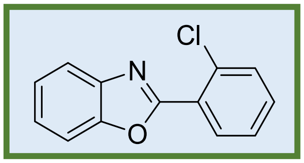

^{1}H NMR (500 MHz, $CDCl_3$)**:** δ 8.16 – 8.14 (m, 1H), 7.87 – 7.84 (m, 1H), 7.64 – 7.61 (m, 1H), 7.58 – 7.56 (m, 1H), 7.47 – 7.37 (m, 4H) ppm.

^{13}C NMR (125 MHz, $CDCl_3$)**:** δ 161.1, 150.8, 141.8, 133.7, 132.1, 132.0, 131.5, 127.1, 126.5, 125.7, 124.8, 120.7, 110.9 ppm.

GC-MS (EI, 70 eV) *m/z*: 229 ($[M]^+$).

2-(2-Hydroxyphenyl)benzoxazole[4]

Analytical TLC on silica gel, 1/19 acetone/petroleum ether.

White solid, mp = 120-122 °C.

FT-IR (KBr, 4000 – 400 cm^{-1}): 2921, 2851, 1630, 1587, 1543, 1487, 1452, 1244, 1155.

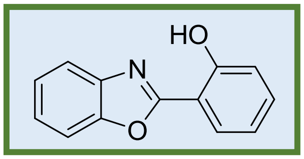

^{1}H NMR (500 MHz, $CDCl_3$)**:** δ 8.04 (dd, J = 8.0, 1.5 Hz, 1H), 7.74 (m, 1H), 7.63 – 7.60 (m, 1H), 7.47 – 7.42 (m, 1H), 7.41 – 7.37 (m, 2H), 7.13 (d, J = 8.5 Hz, 1H), 7.03 – 7.00 (m, 1H) ppm.

^{13}C NMR (125 MHz, $CDCl_3$)**:** δ 133.72, 127.29, 125.54, 125.17, 119.72, 119.43, 117.59, 110.82 ppm.

GC-MS (EI, 70 eV) *m/z*: 213 ($[M]^+$).

2-(Pyridine-4-yl)benzoxazole[8]

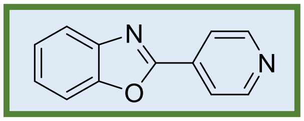

Analytical TLC on silica gel, 1/19 acetone/petroleum ether.

White solid, mp = 102-104°C.

FT-IR (KBr, 4000 – 400 cm^{-1}): 3460, 2946, 1628, 878.

^{1}H NMR (500 MHz, $CDCl_3$): δ 8.82 (d, J = 5.5 Hz, 2H), 8.09 (d, J = 6.0 Hz, 2H), 7.84 – 7.80 (m, 1H), 7.63 (m, 1H), 7.42 (m, 2H) ppm.

^{13}C NMR (125 MHz, $CDCl_3$): δ 161.0, 151.1, 150.9, 141.9, 134.6, 126.5, 125.3, 121.2, 120.9, 111.1 ppm.

GC-MS (EI, 70 eV) *m/z*: 196 ($[M]^+$).

5-Methyl-2-phenylbenzoxazole[3]

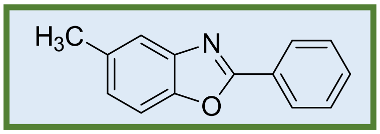

Analytical TLC on silica gel, 1/19 acetone/petroleum ether.

White solid, mp = 112-115 °C.

FT-IR (KBr, 4000 – 400 cm^{-1}): 3422, 2922, 2869, 1647, 1550, 1474, 1334, 1261.

^{1}H NMR (500 MHz, acetone-d_6**)** δ 8.25 – 8.23 (m, 2H), 7.62 − 7.59 (m, 3H), 7.57 – 7.56 (m, 2H), 7.24 (d, J = 8.5, 1H), 2.47 (s, 3H) ppm.

^{13}C NMR (125 MHz, $CDCl_3$): δ 163.3, 149.2, 142.5, 134.5, 131.5, 129.0, 127.7, 127.5, 126.4, 120.1, 110.1, 21.6 ppm.

GC-MS (EI, 70 eV) *m/z*: 209 ($[M]^+$)

5-Methyl-2-(*p*-tolyl)benzoxazole[9]

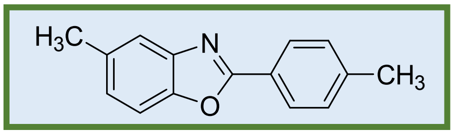

Analytical TLC on silica gel, 1/19 acetone/petroleum ether.

White solid, mp = 134-136 °C.

FT-IR (KBr, 4000 – 400 cm^{-1}): 3304, 2921, 2856, 1615, 1501, 1450, 1333, 1262.

^{1}H NMR (500 MHz, acetone-d_6) δ 8.11 (d, J = 8.0 Hz, 2H), 7.53 – 7.51 (m, 2H), 7.39 (d, J = 8.0 Hz, 2H), 7.22 – 7.19 (m, 1H), 2.46 (s, 3H), 2.43 (s, 3H) ppm.

^{13}C NMR (125 MHz, acetone-d_6) δ 163.2, 149.2, 142.7, 142.3, 134.5, 129.9, 127.5, 126.3, 124.8, 119.9, 110.1, 20.9, 20.8 ppm.

GC-MS (EI, 70 eV) *m/z*: 223 ($[M]^+$)

5-Methyl-2-(4-*tert*-butylphenyl)benzoxazole[6]

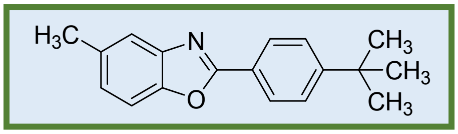

Analytical TLC on silica gel, 1/19 acetone/petroleum ether.

White solid, mp = 138-140 °C.

FT-IR (KBr, 4000 – 400 cm^{-1}): 3107, 2963, 2930, 2871, 1729, 1621, 1532, 1460, 1269.

^{1}H NMR (500 MHz, acetone-d_6) δ 8.56 (d, J = 2.0 Hz, 1H), 8.34 (dd, J = 9.0, 2.0 Hz, 1H), 8.20 (d, J = 8.5 Hz, 2H), 7.92 (d, J = 9.0 Hz, 1H), 7.69 (d, J = 9.0 Hz, 2H), 2.81 (s, 3H), 1.39 (s, 9H) ppm.

^{13}C NMR (125 MHz, acetone-d_6) δ 162.9, 155.0, 149.0, 142.5, 134.3, 127.2, 126.1, 126.0, 124.6, 119.7, 109.9, 34.7, 30.5, 20.5 ppm.

GC-MS (EI, 70 eV) *m/z*: 265 ($[M]^+$).

5-Methyl-2-(4-methoxyphenyl)benzoxazole[9]

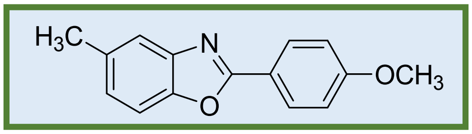

Analytical TLC on silica gel, 1/19 acetone/petroleum ether.

White solid, mp = 112 °C.

FT-IR (KBr, 4000 – 400 cm^{-1}): 2924, 2854, 1730, 1608, 1499, 1420, 1254.

^{1}H NMR (500 MHz, DMSO-d_6) δ 8.12 (d, J = 9.0 Hz, 2H), 7.61 (d, J = 8.0 Hz, 1H), 7.55 (s, 1H), 7.20 (d, J = 8.5 Hz, 1H), 7.15 (d, J = 9.0 Hz, 2H), 3.87 (s, 3H), 2.44 (s, 3H) ppm.

^{13}C NMR (125 MHz, DMSO-d_6) δ 162.5, 148.7, 142.1, 134.7, 129.5, 126.5, 119.6, 119.5, 119.2, 115.2, 110.6, 55.9, 21.3 ppm.

GC-MS (EI, 70 eV) *m/z*: 239 ($[M]^+$).

5-Methyl-2-(4-chlorophenyl)benzoxazole[6]

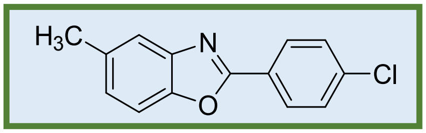

Analytical TLC on silica gel, 1/19 acetone/petroleum ether.

White solid, mp = 124-125 °C.

FT-IR (KBr, 4000 – 400 cm^{-1}): 3050, 2957, 2849, 1615, 1501, 1450, 1420, 1287.

^{1}H NMR (500 MHz, $CDCl_3$): δ 8.18 (d, J = 9.0 Hz, 2H), 7.55 (s, 1H), 7.50 (d, J = 8.5 Hz, 2H), 7.45 (d, J = 8.0 Hz, 1H), 7.17 (d, J = 9.0 Hz, 1H), 2.49 (s, 3H) ppm.

^{13}C NMR (125 MHz, acetone-d_6): δ 162.3, 149.2, 142.4, 137.7, 134.7, 129.3, 128.9, 126.6, 126.0, 120.1, 110.1, 21.6 ppm.

GC-MS (EI, 70 eV) *m/z*: 243 ($[M]^+$).

5-Methyl-2-(4-fluorophenyl)benzoxazole[6]

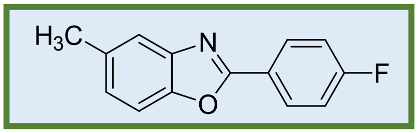

Analytical TLC on silica gel, 1/19 acetone/petroleum ether.

White solid, mp = 170-171 °C.

FT-IR (KBr, 4000 – 400 cm^{-1}): 3050, 2957, 2849, 1615, 1501, 1450, 1420, 1287.

^{1}H NMR (500 MHz, $CDCl_3$) δ 8.27 – 8.17 (m, 2H), 7.53 (s, 1H), 7.42 (d, J = 8.5 Hz, 1H), 7.21 – 7.12 (m, 3H), 2.47 (s, 3H) ppm.

^{13}C NMR (125 MHz, $CDCl_3$): δ 164.9 (d, J = 250.9 Hz), 162.4, 149.2, 142.4, 134.6, 129.9 (d, J = 8.8 Hz), 126.4, 123.8 (d, J = 3 Hz), 120.0, 116.2 (d, J = 22.1 Hz), 110.0, 21.6 ppm

GC-MS (EI, 70 eV) *m/z*: 227 ($[M]^+$)

5-Methyl-2-(4-hydroxyphenyl)benzoxazole[10]

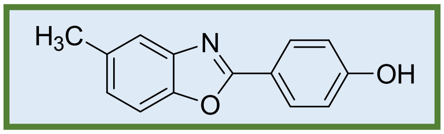

Analytical TLC on silica gel, 1/19 acetone/petroleum ether.

White solid, Yield 81%, mp = 220-221 °C.

FT-IR (KBr, 4000 – 400 cm^{-1}): 3050, 2957, 2849, 1615, 1501, 1450, 1420, 1287.

^{1}H NMR (500 MHz, acetone-d_6): δ 9.23 (s, 1H), 8.10 (d, J = 8.5 Hz, 2H), 7.50 (d, J = 8.5 Hz, 2H), 7.18 (d, J = 8.5 Hz, 1H), 7.03 (d, J = 8.5 Hz, 2H), 2.45 (s, 3H) ppm.

^{13}C NMR (125 MHz, acetone-d_6): δ 161.9, 154.5, 149.0, 143.6, 135.0, 132.8, 130.2, 126.5, 120.3, 116.8, 110.6, 21.4 ppm.

GC-MS (EI, 70 eV) *m/z*: 225 ($[M]^+$).

5-Methyl-2-(pyridin-4-yl)benzoxazole[11]

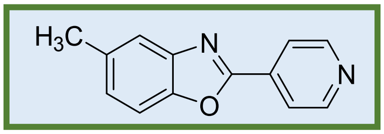

Analytical TLC on silica gel, 1/19 acetone/petroleum ether.

White solid, mp = 129 - 130 °C.

FT-IR (KBr, 4000 – 400 cm^{-1}): 3406, 2921, 1612, 1537, 1414, 1344, 1068, 808.

^{1}H NMR (500 MHz, $CDCl_3$): δ 8.80 (d, J = 4.5 Hz, 2H), 8.07 (d, J = 5.0 Hz, 2H), 7.59 (s, 1H), 7.48 (d, J = 8.5 Hz, 1H), 7.23 (d, J = 8.0 Hz, 1H), 2.50 (s, 3H) ppm.

^{13}C NMR (125 MHz, $CDCl_3$): δ 160.8, 150.7, 149.3, 142.1, 135.2, 134.8, 127.7, 121.1, 120.6, 110.4, 21.6 ppm.

GC-MS (EI, 70 eV) *m/z*: 210 ($[M]^+$).

5-Chloro-2-phenylbenzoxazole[7]

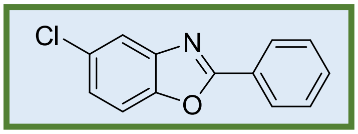

Analytical TLC on silica gel, 1/19 acetone/petroleum ether.

White solid, mp = 102-104 °C.

FT-IR (KBr, 4000 – 400 cm^{-1}): 3061, 1612, 1551, 1443, 1333, 1265.

^{1}H NMR (500 MHz, acetone-d_6) δ 8.23 (d, J = 7.5 Hz, 2H), 7.77 (d, J = 1.5 Hz, 1H), 7.70 (d, J = 7.5 Hz, 1H), 7.59 – 7.64 (m, 3H), 7.42 (dd, J = 9.0, 1.5 Hz, 1H) ppm.

^{13}C NMR (125 MHz, acetone-d_6) δ 165.3, 150.6, 144.5, 133.1, 130.7, 130.2, 128.6, 127.7, 126.4, 120.7, 112.9 ppm.

GC-MS (EI, 70 eV) *m/z*: 229 ($[M]^+$).

5-Chloro-2-(*p*-tolyl)benzoxazole[12]

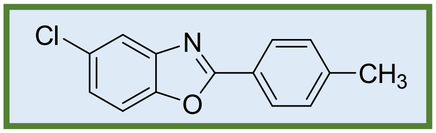

Analytical TLC on silica gel, 1/19 acetone/petroleum ether.

White solid, mp = 138-140 °C.

FT-IR (KBr, 4000 – 400 cm^{-1}): 2925, 1610, 1551, 1479, 1258.

^{1}H NMR (500 MHz, acetone-d_6) δ 8.13 (d, J = 8.0 Hz, 2H), 7.75 (d, J = 2.0 Hz, 1H), 7.70 (d, J = 9.0 Hz, 1H), 7.40 – 7.33 (m, 4H), 2.44 (s, 3H) ppm.

^{13}C NMR (125 MHz, acetone-d_6) δ 165.6, 150.5, 144.6, 143.8, 130.8, 130.6, 128.6, 126.2, 125.0, 120.5, 112.8, 21.7 ppm.

GC-MS (EI, 70 eV) *m/z*: 243 ($[M]^+$).

5-Chloro-2-(4-*tert*-butylphenyl)benzoxazole[13]

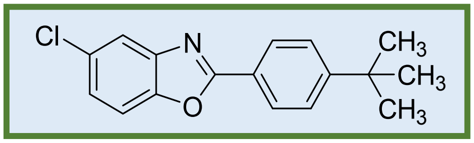

Analytical TLC on silica gel, 1/19 acetone/petroleum ether.

White solid, mp = 140-142 °C.

FT-IR (KBr, 4000 – 400 cm^{-1}): 2957, 2902, 2866, 1611, 1552, 1493, 1457, 1262.

^{1}H NMR (500 MHz, acetone-d_6) δ 8.17 (d, J = 8.5 Hz, 2H), 7.76 (s, 1H), 7.70 (d, J = 8.5 Hz, 1H), 7.66 (d, J = 8.5 Hz, 2H), 7.41 (dd, J = 8.5, 2.0 Hz, 1H), 1.38 (s, 9H) ppm.

^{13}C NMR (125 MHz, acetone-d_6) δ 165.5, 156.7, 150.6, 144.6, 130.6, 128.6, 127.2, 126.2, 125.0, 120.5, 112.8, 35.9, 31.5 ppm.

GC-MS (EI, 70 eV) *m/z*: 285 ($[M]^+$).

5-Chloro-2-(4-methoxyphenyl)benzoxazole[12]

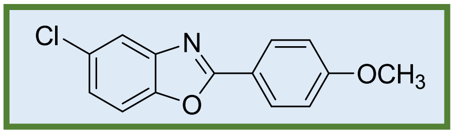

Analytical TLC on silica gel, 1/19 acetone/petroleum ether.

White solid, mp = 148-150 ^{o}C.

FT-IR (KBr, 4000 – 400 cm^{-1}): 2962, 1610, 1551, 1479, 1448, 1258.

^{1}**H NMR** (500 MHz, acetone-d_6) δ 8.19 – 8.17 (m, 2H), 7.72 (d, J = 2.0 Hz, 1H), 7.67 (d, J = 8.5 Hz, 1H), 7.38 (dd, J = 8.5, 2.0 Hz, 1H), 7.16 – 7.14 (m, 2H), 3.92 (s, 3H) ppm.

^{13}**C NMR** (125 MHz, acetone-d_6) δ 165.5, 164.1, 150.5, 144.8, 130.5, 126.6, 125.8, 120.3, 120.0, 115.7, 112.6, 56.2 ppm.

GC-MS (EI, 70 eV) *m/z*: 259 ([M]$^+$).

5-Chloro-2-(4-fluororophenyl)benzoxazole[14]

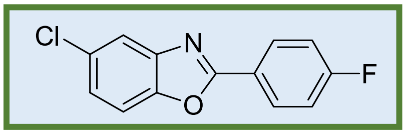

Analytical TLC on silica gel, 1/19 acetone/petroleum ether.

White solid, mp = 156-157 ^{o}C.

FT-IR (KBr, 4000 – 400 cm^{-1}): 3061, 2922, 1610, 1551, 1481, 1450, 1331, 1260, 1227.

^{1}**H NMR** (500 MHz, acetone-d_6) δ 8.31 (dd, J = 8.5, 5.5 Hz, 2H), 7.78 (s, 1H), 7.73 (d, J = 8.6 Hz, 1H), 7.47 – 7.38 (m, 3H) ppm.

^{13}**C NMR** (125 MHz, acetone-d_6): δ 166.0 (d, J = 250 Hz), 164.3, 150.5, 144.3, 131.1 (d, J = 9.0 Hz), 130.6, 126.3, 124.2, 120.5, 117.2 (d, J = 22.4 Hz), 112.7 ppm.

GC-MS (EI, 70 eV) *m/z*: 247 ([M]$^+$)

5-Chloro-2-(4-chlorophenyl)benzoxazole[12]

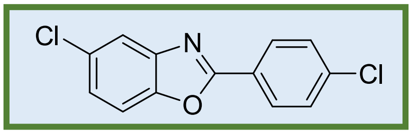

Analytical TLC on silica gel, 1/19 acetone/petroleum ether.

White solid, mp = 190-192 °C.

FT-IR (KBr, 4000 – 400 cm^{-1}): 2925, 1610, 1549, 1542, 1480, 1449, 1260, 1197.

^{1}H NMR (500 MHz, acetone-d_6) δ 8.28 – 8.23 (m, 2H), 7.80 (d, J = 1.5 Hz, 1H), 7.74 (d, J = 8.5 Hz, 1H), 7.69 – 7.65 (m, 2H), 7.46 (dd, J = 8.5, 1.5 Hz, 1H) ppm.

^{13}C NMR (125 MHz, acetone-d_6): δ 164.6, 150.7, 144.4, 138.8, 130.9, 130.5, 130.3, 126.8, 126.6, 120.8, 113.0 ppm.

GC-MS (EI, 70 eV) *m/z*: 263.

5-Chloro-2-(4-hydroxyphenyl)benzoxazole[10]

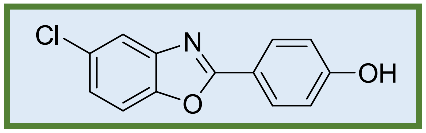

Analytical TLC on silica gel, 1/19 acetone/petroleum ether.

White solid, mp = 185-187 °C.

FT-IR (KBr, 4000 – 400 cm^{-1}): 3162, 2920, 2850, 1666, 1596, 1553, 1446, 1448, 1333, 1286, 1217.

^{1}H NMR (500 MHz, acetone-d_6): δ 8.13 – 8.09 (m, 2H), 7.69 (dd, J = 8.5, 2.0 Hz, 2H), 7.38 (dd, J = 8.5, 2.0 Hz, 1H), 7.07 – 7.04 (m, 2H) ppm.

^{13}C NMR (125 MHz, acetone-d_6): δ 162.1, 150.2, 144.7, 130.6, 125.5, 121.6, 120.0, 118.9, 117.0, 112.4, 88.3 ppm.

GC-MS (EI, 70 eV) *m/z*: 245 ($[M]^+$).

5-Chloro-2-(pyridin-4-yl)benzoxazole[11]

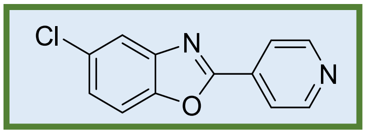

Analytical TLC on silica gel, 1/19 acetone/petroleum ether.

White solid, mp = 152 - 153 °C.

FT-IR (KBr, 4000 – 400 cm^{-1}): 3431, 2922, 1612, 1568, 1539, 1449, 1057, 874.

^{1}H NMR (500 MHz, ($CDCl_3$): δ 8.83 (d, J = 5.5 Hz, 2H), 8.07 (dd, J = 4.5, 1.5 Hz, 2H), 7.80 (d, J = 2.0 Hz, 1H), 7.55 (d, J = 8.5 Hz, 1H), 7.40 (dd, J = 8.5, 2.0 Hz, 1H) ppm.

^{13}C NMR (125 MHz, ($CDCl_3$): δ 162.0, 150.8, 149.6, 143.0, 134.2, 130.9, 126.9, 121.3, 120.8, 111.9 ppm.

GC-MS (EI, 70 eV) *m/z*: 230 ($[M]^+$).

5-Nitro-2-phenylbenzoxazole[12]

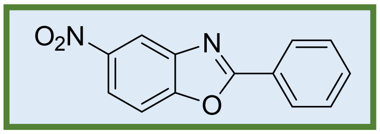

Analytical TLC on silica gel, 1/19 acetone/petroleum ether.

Yellow solid, mp = 167-168 °C.

FT-IR (KBr, 4000 – 400 cm^{-1}): 3107, 2922, 2852, 1704, 1604, 1525, 1473, 1448, 1247, 1285.

^{1}H NMR (500 MHz, DMSO-d_6) δ 8.68 (d, J = 2.5 Hz, 1H), 8.35 (dd, J = 9.0, 2.0 Hz, 1H), 8.26 – 8.24 (m, 2H), 8.06 (d, J = 9.0 Hz, 1H), 7.71 – 7.65 (m, 3H) ppm.

^{13}C NMR (125 MHz, DMSO-d_6) δ 165.4, 154.0, 145.1, 141.9, 132.9, 129.5, 127.8, 125.5, 121.5, 115.7, 111.8 ppm.

GC-MS (EI, 70 eV) *m/z*: 240 ($[M]^+$)

5-Nitro-2-(*p*-tolyl)benzoxazole[15]

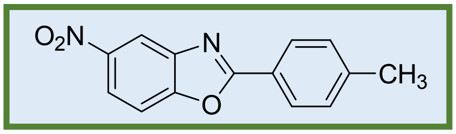

Analytical TLC on silica gel, 1/19 acetone/petroleum ether.

Yellow solid, mp = 125-126 ^{o}C.

FT-IR (KBr, 4000 – 400 cm^{-1}): 3467, 3106, 2964, 2869, 1723, 1531, 1462, 1420, 1130.

^{1}H NMR (500 MHz, acetone-d_6) δ 8.31 – 8.27 (m, 2H), 7.57 – 7.55 (m, 2H), 7.40 – 7.35 (m, 2H), 7.24 (d, J = 9.0 Hz, 1H), 2.47 (s, 3H) ppm.

^{13}C NMR (125 MHz, acetone-d_6) δ 166.7, 164.7, 150.0, 143.3, 135.5, 130.8, 130.7, 127.30, 124.8, 120.7, 117.2, 117.0, 110.9, 21.4 ppm.

GC-MS (EI, 70 eV) *m/z*: 254 ([M]$^{+}$)

5-Nitro-2-(4-*tert*-butylphenyl)benzoxazole[16]

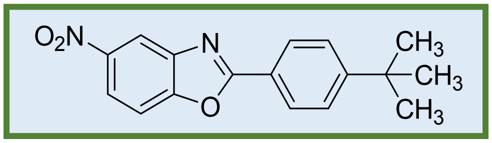

Analytical TLC on silica gel, 1/19 acetone/petroleum ether.

White solid, mp = 154-156 ^{o}C.

FT-IR (KBr, 4000 – 400 cm^{-1}): 3106, 2964, 2869, 1723, 1531, 1462, 1410, 1267.

^{1}H NMR (500 MHz, acetone-d_6) δ 8.18 – 8.16 (m, 2H), 7.66 – 7.63 (m, 2H), 7.56 – 7.54 (m, 2H), 7.23 – 7.21 (m, 1H), 1.38 (s, 9H) ppm.

^{13}C NMR (125 MHz, acetone-d_6) δ 166.9, 157.3, 155.3, 146.4, 143.6, 132.1, 129.7, 128.7, 127.2, 124.2, 122.0, 116.4, 112.1, 35.8, 31.4 ppm.

GC-MS (EI, 70 eV) *m/z*: 296 ([M]$^{+}$)

5-Nitro-2-(4-methoxyphenyl)benzoxazole[12]

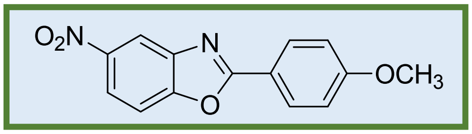

Analytical TLC on silica gel, 1/19 acetone/petroleum ether.

Yellow solid, mp = 182-183 ^{o}C.

FT-IR (KBr, 4000 – 400 cm^{-1}): 3457, 2964, 1723, 1631, 1462, 1410, 1267.

^{1}H NMR (500 MHz, acetone-d_6) δ 8.55 (d, J = 2.5 Hz, 1H), 8.34 (dd, J = 9.0, 2.5 Hz, 1H), 8.25 – 8.23 (m, 2H), 7.91 (d, J = 9.0 Hz, 1H), 7.20 – 7.18 (m, 2H), 3.95 (s, 3H) ppm.

^{13}C NMR (125 MHz, acetone-d_6) δ 163.5, 154.4, 142.8, 129.8, 120.8, 118.3, 115.2, 114.8, 111.0, 55.2 ppm.

GC-MS (EI, 70 eV) *m/z*: 270 ([M]$^+$)

5-Nitro-2-(4-fluorophenyl)benzoxazole[15]

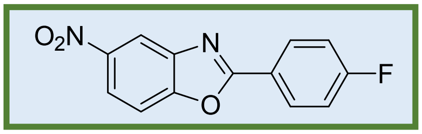

Analytical TLC on silica gel, 1/19 acetone/petroleum ether.

White solid, Yield 74%, mp = 202-205 ^{o}C.

FT-IR (KBr, 4000 – 400 cm^{-1}): 3061, 2925, 1650, 1584, 1582, 1542, 1473, 1448, 1247, 1225.

^{1}H NMR (500 MHz, acetone-d_6): δ 8.60 (d, J = 2.3 Hz, 1H), 8.39 – 8.35 (m, 3H), 7.96 (d, J = 8.9 Hz, 1H), 7.47 – 7.41 (m, 2H) ppm.

^{13}C NMR (125 MHz, acetone-d_6): δ 166.2 (d, J = 250.0 Hz), 155.3, 131.3 (d, J = 10.0 Hz), 123.5, 122.0, 117.3 (d, J = 22.5 Hz), 116.5, 112.1 ppm.

GC-MS (EI, 70 eV) *m/z*: 258 ([M]$^+$).

5-Nitro-2-(4-chlorophenyl)benzoxazole[15]

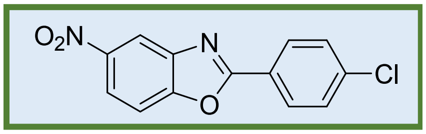

Analytical TLC on silica gel, 1/19 acetone/petroleum ether.

White solid, mp = 207-210 ^{o}C.

FT-IR (KBr, 4000 – 400 cm^{-1}): 3420, 3105, 2964, 2853, 1723, 1528, 1434, 1410, 1256.

^{1}H NMR (500 MHz, acetone-d_6): δ 8.62 (d, J = 2.0 Hz, 1H), 8.39 (dd, J = 8.5, 2.0 Hz, 1H), 8.31 – 8.29 (m, 2H), 7.97 (d, J = 9 Hz, 1H), 7.72 – 7.68 (m, 2H) ppm.

^{13}C NMR (125 MHz, acetone-d_6): δ 155.4, 139.3, 130.4, 130.3, 129.5, 125.3, 122.2, 116.6, 114.8, 112.2, 103.5ppm.

GC-MS (EI, 70 eV) m/z: 274 ([M]$^{+}$).

Characterization of 2-arylbezothiazoles

2-Phenylbenzothiazole[17]

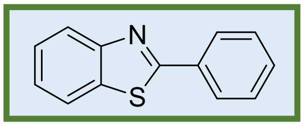

Analytical TLC on silica gel, 1/19 ethyl acetate/hexanes.

White solid, mp = 125-126 °C.

FT-IR (KBr, 4000 – 400 cm^{-1}): 3435, 3063, 1508, 1283, 1027, 763.

^{1}H NMR (500 MHz, $CDCl_3$) δ 8.12 – 8.08 (m, 3H), 7.91 (d, J = 8.0 Hz, 1H), 7.52 – 7.48 (m, 4H), 7.41 – 7.36 (m, 1H) ppm.

^{13}C NMR (125 MHz, $CDCl_3$): δ 168.2, 154.3, 135.2, 133.8, 131.1, 129.2, 127.7, 126.5, 125.3, 123.4, 121.6 ppm.

GC-MS (EI, 70 eV) m/z: 211 ([M]$^{+}$)

2-(4-Methylphenyl)benzothiazole[17]

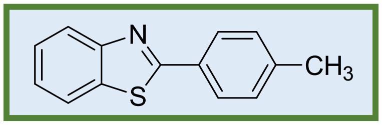

Analytical TLC on silica gel, 1/19 ethyl acetate/hexanes.

White solid, mp = 95 – 96 °C.

FT-IR (KBr, 4000 – 400 cm^{-1}): 3427, 2919, 1605, 1477, 1216.

^{1}H NMR (500 MHz, $CDCl_3$): δ 8.10 – 8.04 (m, 1H), 7.99 (d, J = 8.0 Hz, 2H), 7.88 (dd, J = 8.0, 0.5 Hz, 1H), 7.50 – 7.46 (m, 1H), 7.39 – 7.35 (m, 1H), 7.29 (d, J = 8.0 Hz, 2H), 2.42 (s, 3H) ppm.

^{13}C NMR (125 MHz, $CDCl_3$): δ 168.3, 154.3, 141.5, 135.1, 131.1, 129.8, 127.6, 126.4, 125.1, 123.2, 121.7, 21.6 ppm.

GC-MS (EI, 70 eV) m/z: 225 ([M]$^{+}$)

2-(4-Methoxyphenyl)benzothiazole[17]

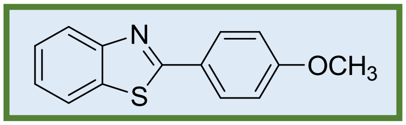

Analytical TLC on silica gel, 1/19 ethyl acetate/hexanes.

White solid, mp = 123-124 °C.

FT-IR (KBr, 4000 – 400 cm^{-1}): 3408, 2926, 1599, 1478, 1255, 1023, 830.

^{1}H NMR (500 MHz, $CDCl_3$): δ 8.06 – 8.01 (m, 3H), 7.88 (d, J = 8.0 Hz, 1H), 7.49 – 7.45 (m, 1H), 7.38 – 7.33 (m, 1H), 7.02 – 6.98 (m, 2H), 3.89 (s, 3H) ppm.

^{13}C NMR (125 MHz, $CDCl_3$): δ 168.0, 162.1, 154.3, 135.0, 129.3, 126.6, 126.4, 125.0, 123.0, 121.7, 114.5, 55.6 ppm.

GC-MS (EI, 70 eV) *m/z*: 241 ($[M]^+$)

2-(4-Fluorophenyl)benzothiazole[17]

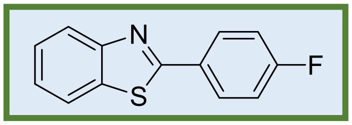

Analytical TLC on silica gel, 1/19 ethyl acetate/hexanes.

White solid, mp = 110 °C.

FT-IR (KBr, 4000 – 400 cm^{-1}): 3430, 2922, 1639, 1480, 1226, 964.

^{1}H NMR (500 MHz, $CDCl_3$): δ 8.10 – 8.05 (m, 3H), 7.89 (d, J = 8.0 Hz, 1H), 7.52 – 7.48 (m, 1H), 7.41 – 7.37 (m, 1H), 7.20 – 7.16 (m, 2H) ppm.

^{13}C NMR (125 MHz, $CDCl_3$): δ 166.9, 164.0 (d, J = 250 Hz), 154.3, 135.2, 130.1 (d, J = 2.5 Hz), 129.5 (d, J = 8.8 Hz), 126.5, 125.4, 123.4, 121.7, 116.1 (d, J = 22.5 Hz) ppm.

GC-MS (EI, 70 eV) *m/z*: 229 ($[M]^+$).

2-(4-Chlorophenyl)benzothiazole[17]

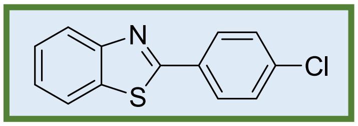

Analytical TLC on silica gel, 1/19 ethyl acetate/hexanes.

White solid, mp = 119-120 °C.

FT-IR (KBr, 4000 – 400 cm^{-1}): 3432, 3053, 1635, 1505, 1311, 1086, 826.

^{1}H NMR (500 MHz, $CDCl_3$) δ 8.09 – 8.05 (m, 1H), 8.04 – 8.01 (m, 2H), 7.90 (d, J = 8.0 Hz, 1H), 7.52 – 7.44 (m, 3H), 7.40 (m, 1H) ppm.

^{13}C NMR (125 MHz, $CDCl_3$): δ 166.8, 154.3, 137.2, 135.2, 132.3, 129.4, 128.9, 126.6, 125.6, 123.5, 121.8 ppm.

GC-MS (EI, 70 eV) *m/z*: 245 ($[M]^+$)

2-(4-Nitrophenyl)benzothiazole[17]

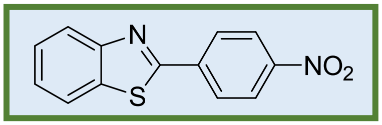

Analytical TLC on silica gel, 1/19 ethyl acetate/hexanes.

Yellow solid, Yield 75%, mp = 207 °C.

FT-IR (KBr, 4000 – 400 cm^{-1}): 3429, 2922, 1518, 1339, 1101, 965.

^{1}H NMR (500 MHz, $CDCl_3$): δ 8.37 – 8.34 (m, 2H), 8.29 – 8.26 (m, 2H), 8.13 (d, J = 8.0 Hz, 1H), 7.96 (d, J = 8.0 Hz, 1H), 7.58 – 7.53 (m, 1H), 7.48 – 7.45 (m, 1H) ppm.

^{13}C NMR (125 MHz, $CDCl_3$): δ 165.0, 154.3, 149.2, 139.3, 135.7, 128.4, 127.1, 126.4, 124.5, 124.1, 122.0 ppm.

GC-MS (EI, 70 eV) *m/z*: 256 ($[M]^+$)

Characterization of 2-arylbezimidazoles

2-Phenylbenzimidazole[5,3,18]

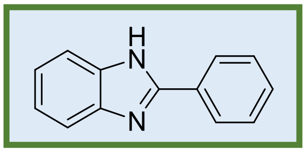

Analytical TLC on silica gel, 1/19 ethyl acetate/petroleum ether, mp = 293-294 °C.

FT-IR (KBr, 4000 – 400 cm^{-1}): 3411, 1540, 1450, 1408, 967, 774.

^{1}H NMR (500 MHz, DMSO-d_6): δ 8.18 (d, J = 7.5 Hz, H), 7.60 (s, 1H), 7.57 – 7.55 (m, 2H), 7.51 – 7.48 (m, 1H), 7.22 – 7.19 (m, 2H) ppm.

^{13}C NMR (125 MHz, DMSO-d_6): δ 151.2, 130.2, 129.8, 128.9, 126.4, 122.1 ppm.

GC-MS (EI, 70 eV) *m/z* 195 ($[M]^+$).

2-(4-Methoxyphenyl)benzimidazole[5]

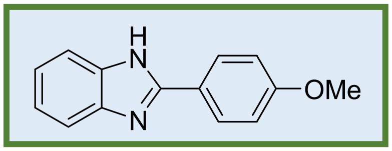

Analytical TLC on silica gel, 1/19 ethyl acetate/petroleum ether, mp = 204 °C.

FT-IR (KBr, 4000 – 400 cm^{-1}): 3395, 3055, 1452, 1406, 1274, 741.

^{1}H NMR (500 MHz, DMSO-d_6): δ 8.49 (s, 1H), 8.05 (d, J = 7.5 Hz, 1H), 7.88 (d, J = 9Hz, 2H), 7.81 (d, J = 8.0 Hz, 1H), 7.47 – 7.41 (m, 2H), 7.17 (d, J = 8.5 Hz, 2H), 3.90 (s, 3H) ppm.

^{13}C NMR (125 MHz, DMSO-d_6): δ 166.4, 163.2, 144.3, 143.7, 132.4, 132.1, 131.6, 125.1, 124.7, 124.5, 120.1, 115.0, 114.4, 55.7 ppm.

GC-MS (EI, 70 eV) *m/z* 225 ($[M]^+$).

2-(4-Fluorophenyl)benzimidazole[5]

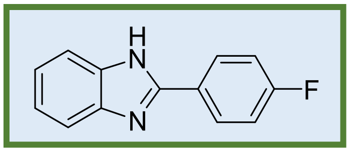

Analytical TLC on silica gel, 1/9 ethyl acetate/petroleum ether, mp = 253-254 °C

FT-IR (KBr, 4000 – 400 cm^{-1}): 3429, 3060, 1604, 1440, 964, 743.

^{1}H NMR (500 MHz, DMSO-d_6): δ 8.23 – 8.21 (m, 2H), 7.59 – 7.58 (m, 2H), 7.42 – 7.38 (m, 2H), 7.22 – 7.18 (m, 2H) ppm.

^{13}C NMR (125 MHz, DMSO-d_6): δ 163.0 (d, *J* = 245.0 Hz), 150.4, 131.3, 128.7 (d, *J* = 8.75 Hz), 126.8, 122.1, 116.0, 115.9 ppm.

GC-MS (EI, 70 eV) *m/z* 213 ($[M]^+$).

2-(4-Nitrophenyl)benzimidazole[5]

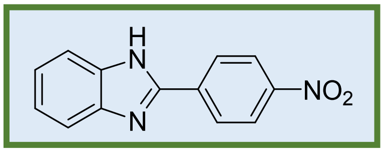

Analytical TLC on silica gel, 1/9 ethyl acetate/petroleum ether, mp = 234-236 °C

FT-IR (KBr, 4000 – 400 cm^{-1}): 3321, 3047, 1517, 1340, 853, 741.

^{1}H NMR (500 MHz, DMSO-d_6): δ 7.89 – 7.87 (m, 2H), 7.82 – 7.80 (m, 1H), 7.77 – 7.74 (m, 1H), 7.66 – 7.63 (m, 2H), 7.49 – 7.43 (m, 2H) ppm.

^{13}C NMR (125 MHz, DMSO-d6): δ 167.5, 144.3, 143.8, 141.9, 133.1, 132.7, 131.9, 129.7, 129.0, 125.4, 125.0, 120.1, 115.2 ppm.

GC-MS (EI, 70 eV) *m/z* 240 ($[M]^+$).

Section S5. ^{1}H and ^{13}C NMR spectroscopy

Triphenyl(butyl-4-sulphonyl)phosphonium toluenesulfonate

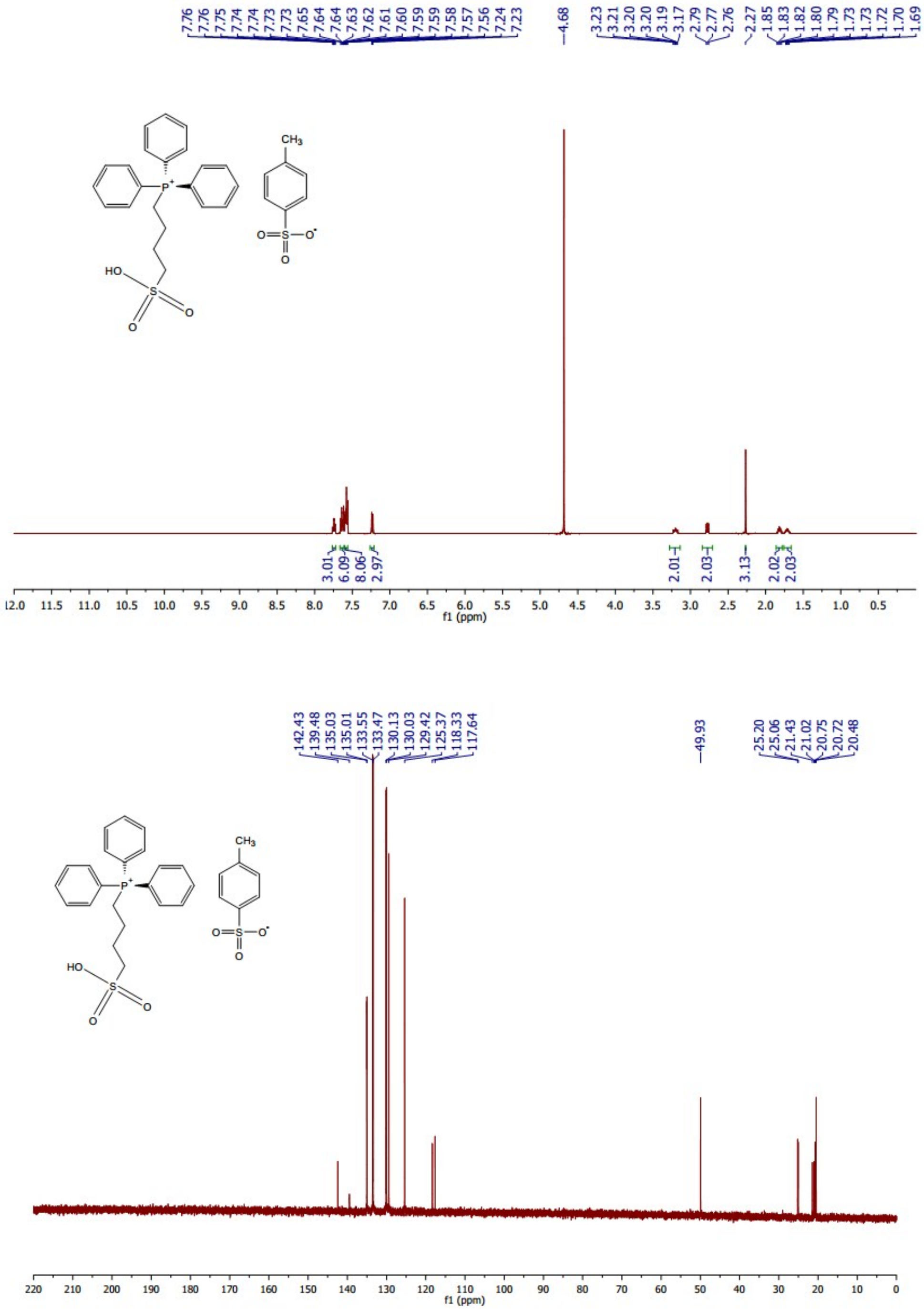

Figure S2. ^{1}H and ^{13}C NMR spectra of triphenyl(butyl-4-sulphonyl)phosphonium toluenesulfonate

Characterization of 2-arylbezoxazoles

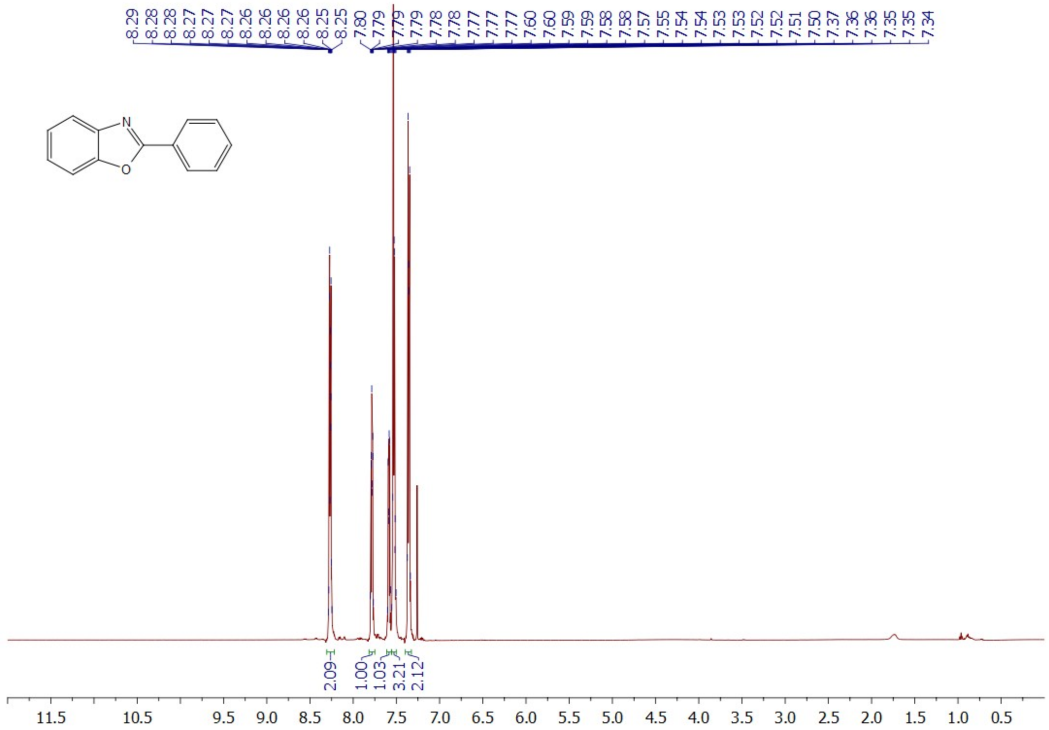

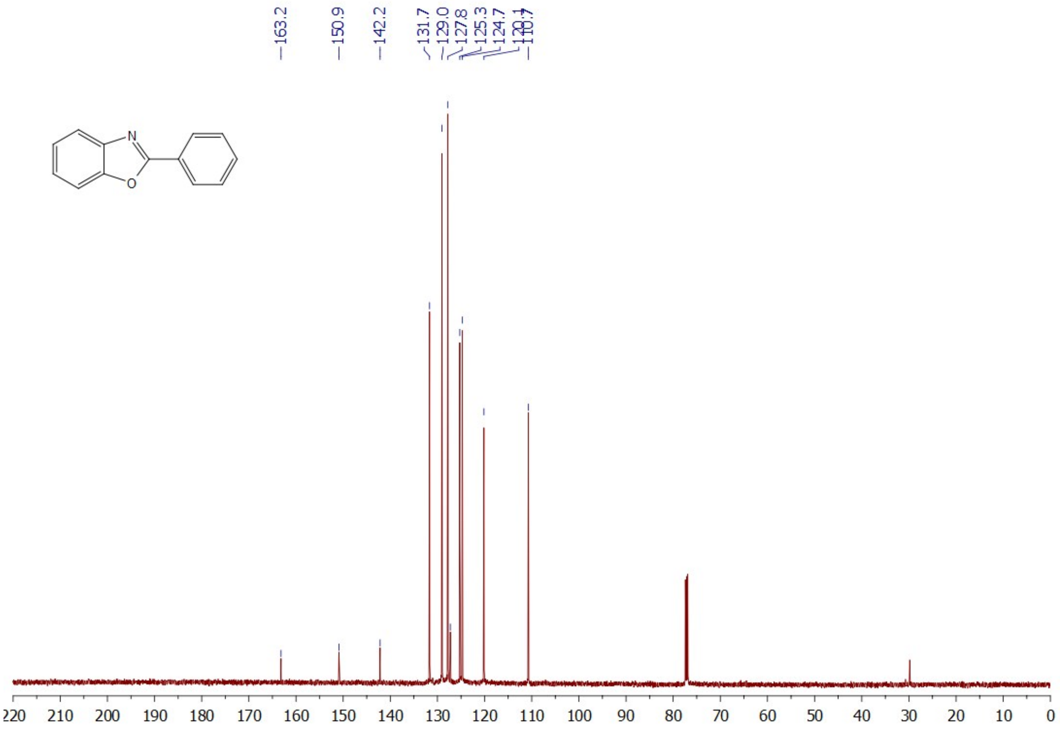

Figure S3. ^{1}H and ^{13}C NMR spectra of 2-phenylbenzoxazole

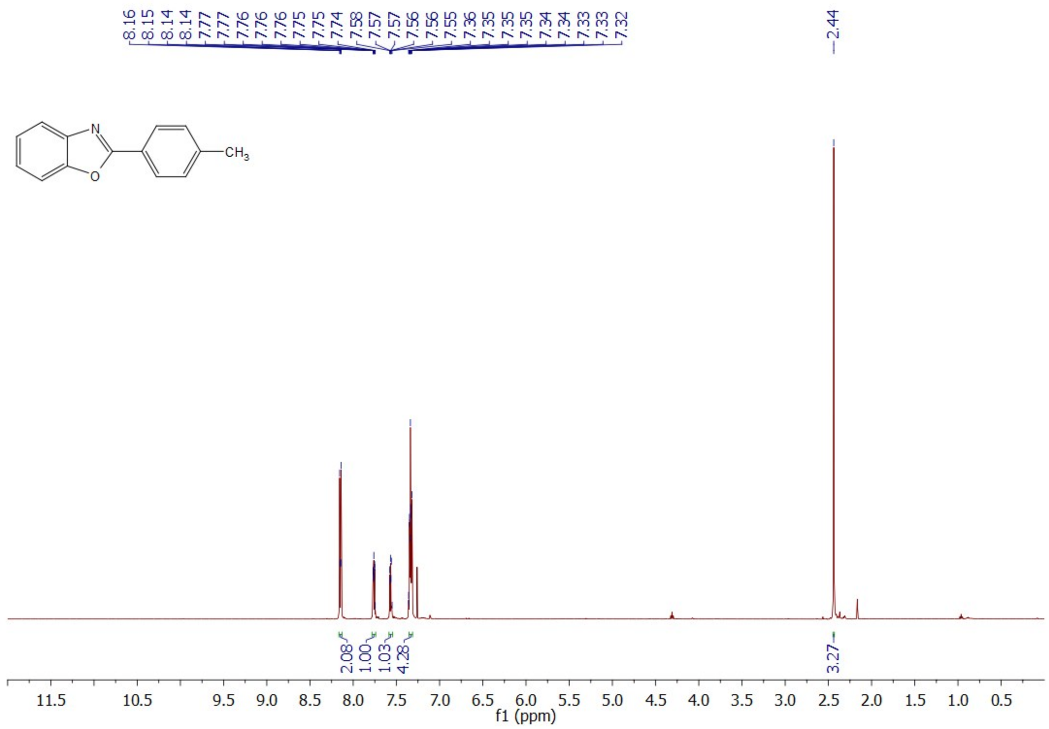

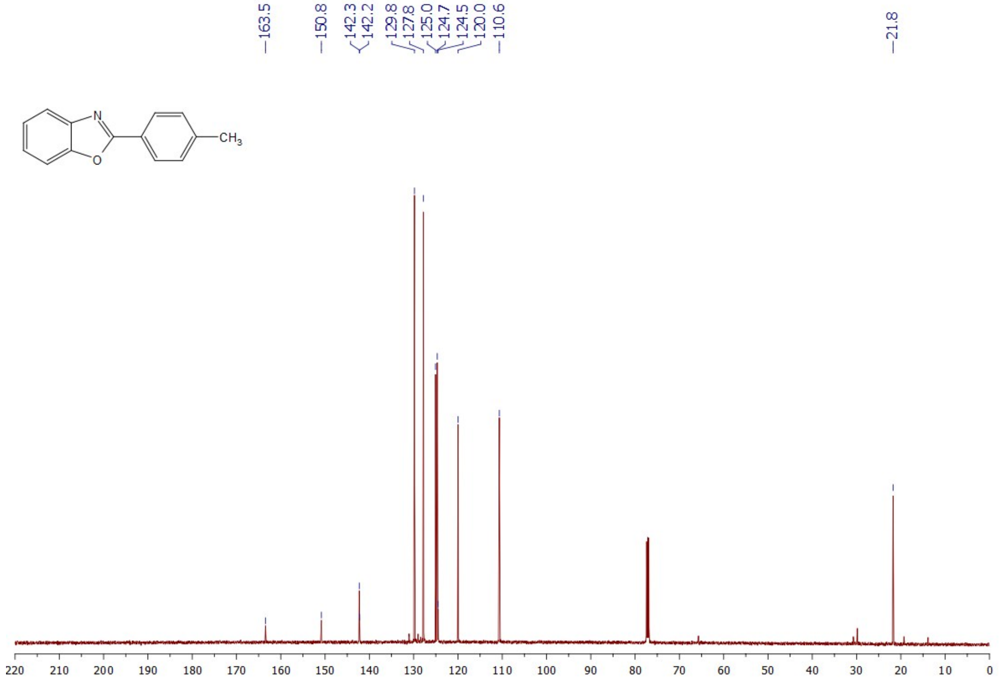

Figure S4. ^{1}H and ^{13}C NMR spectra of 2-(4-methylphenyl)benzoxazole

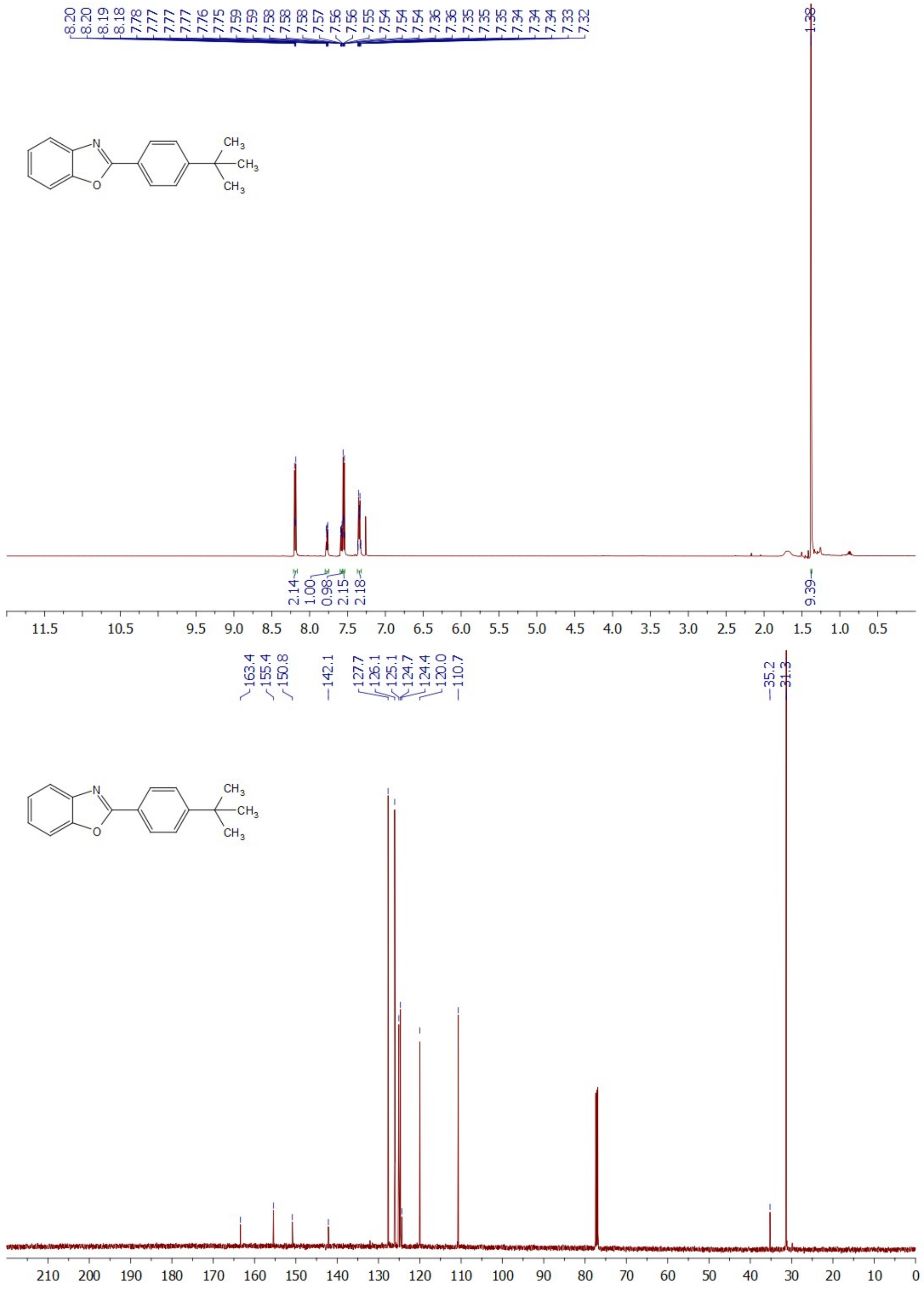

Figure S5. ^{1}H and ^{13}C NMR spectra of 2-(4-*tert*-butylphenyl)benzoxazole

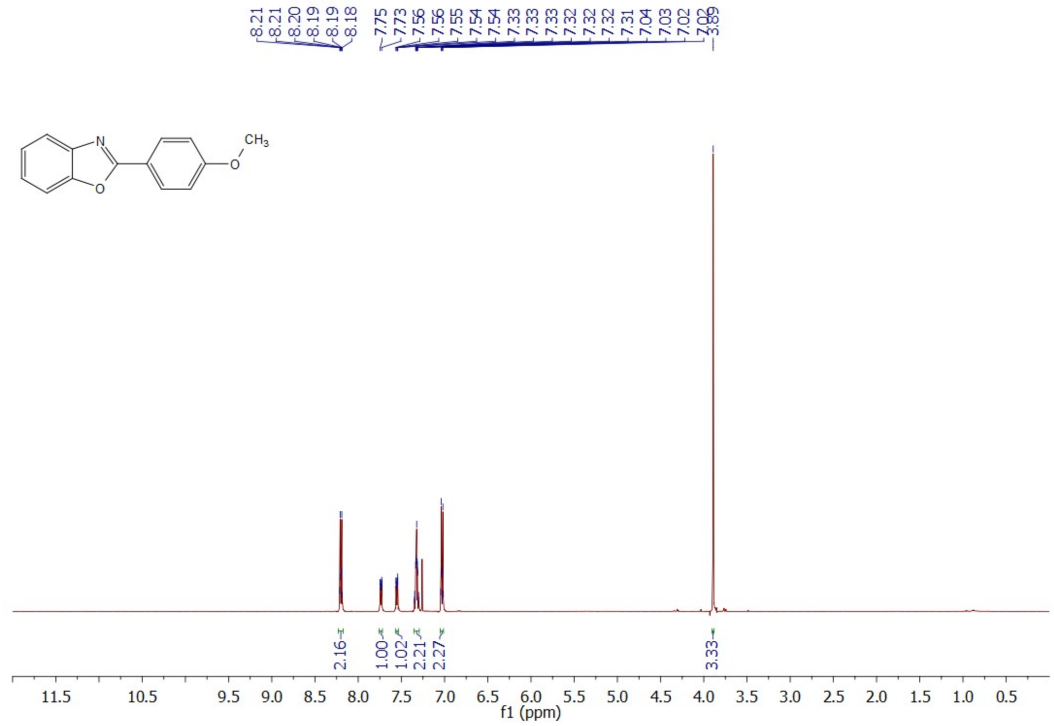

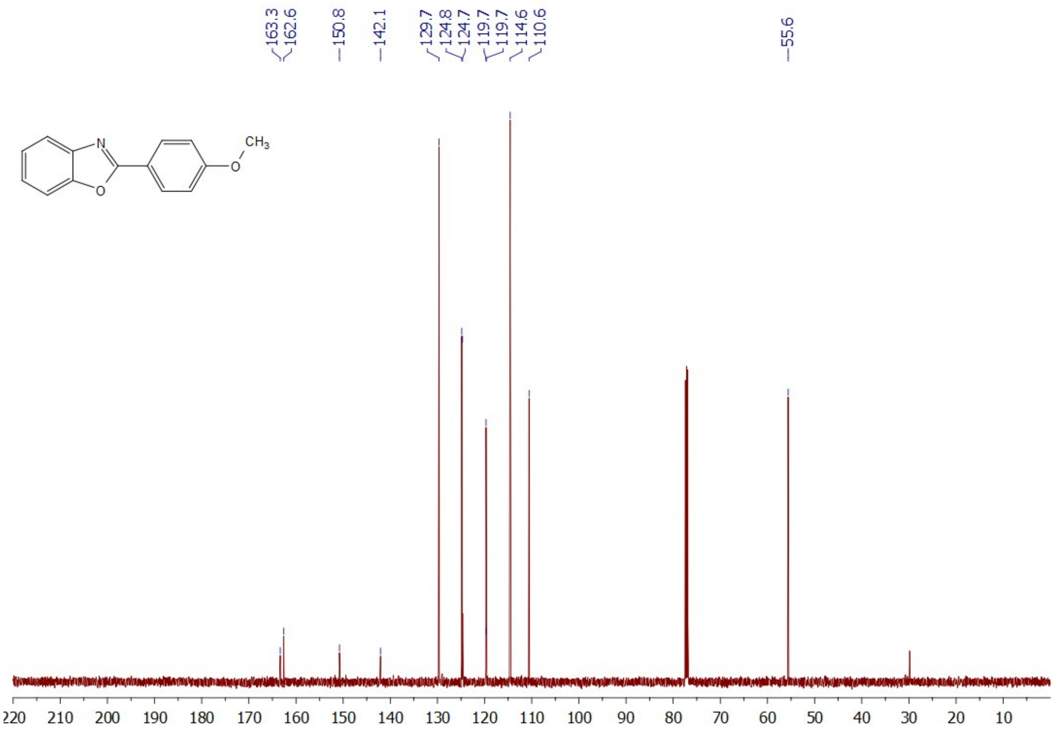

Figure S6. ^{1}H and ^{13}C NMR spectra of 2-(4-methoxyphenyl)benzoxazole

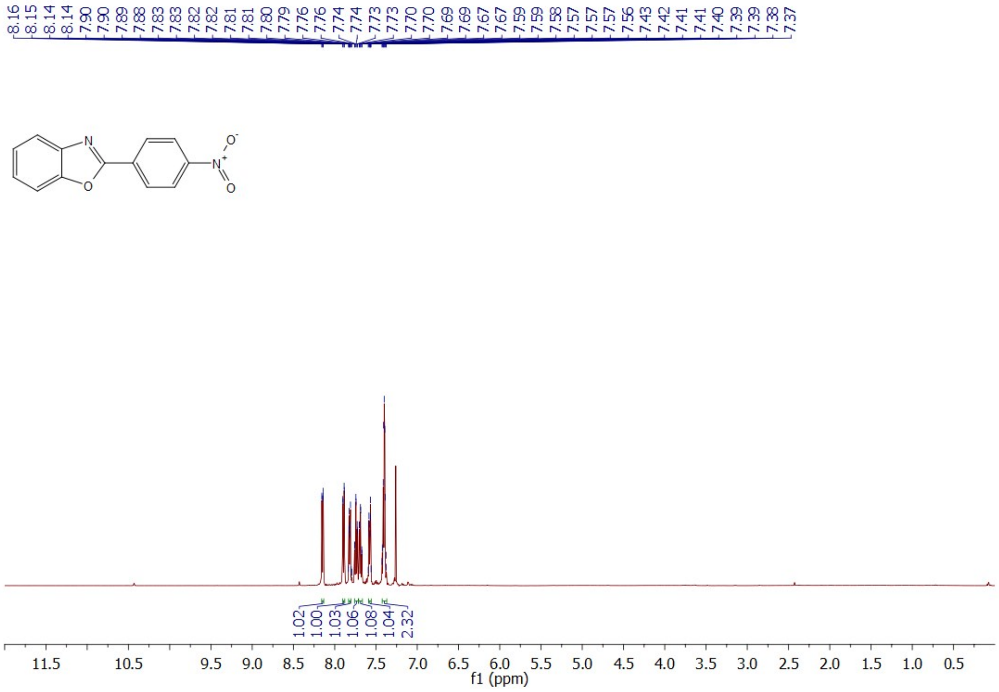

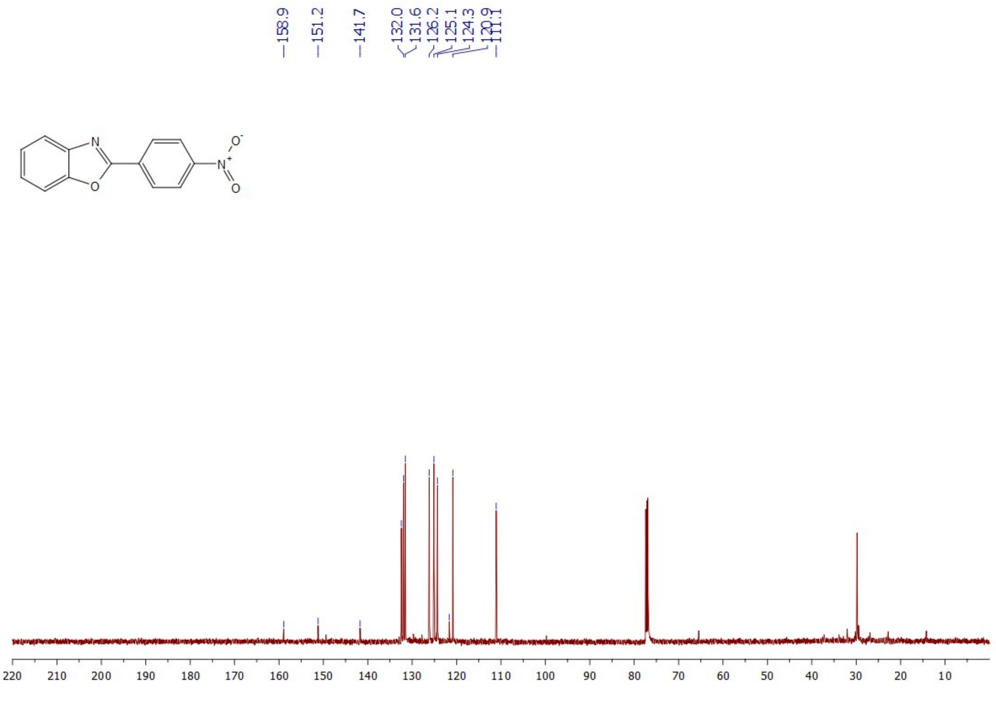

Figure S7. ^{1}H and ^{13}C NMR spectra of 2-(4-nitrophenyl)benzoxazole

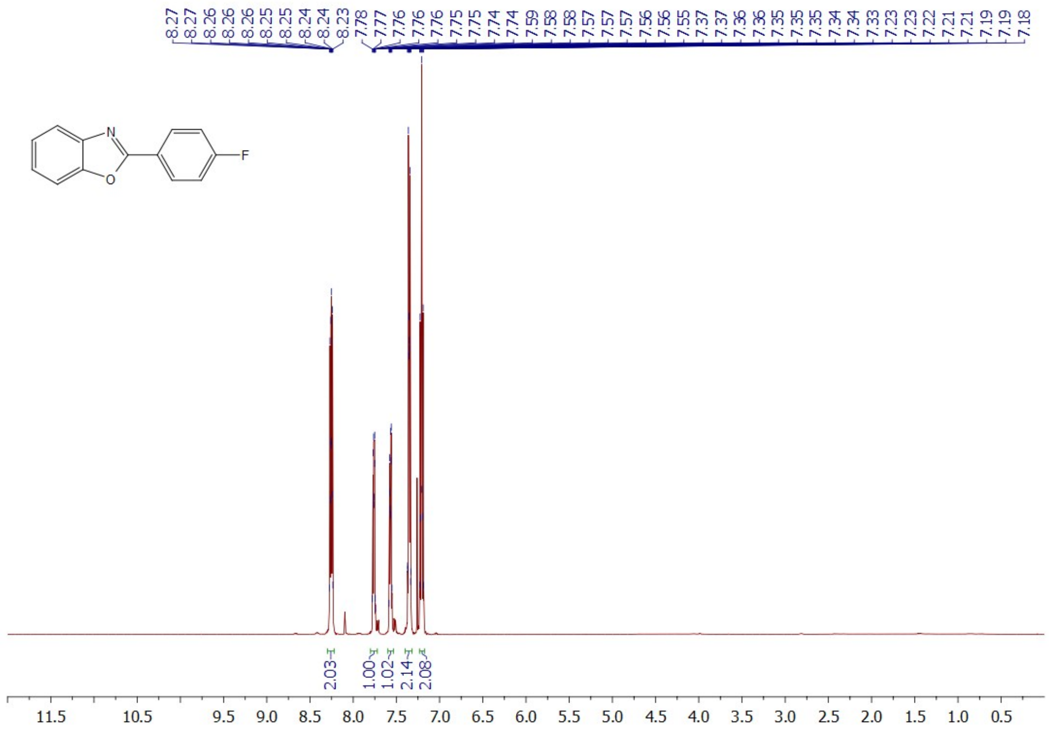

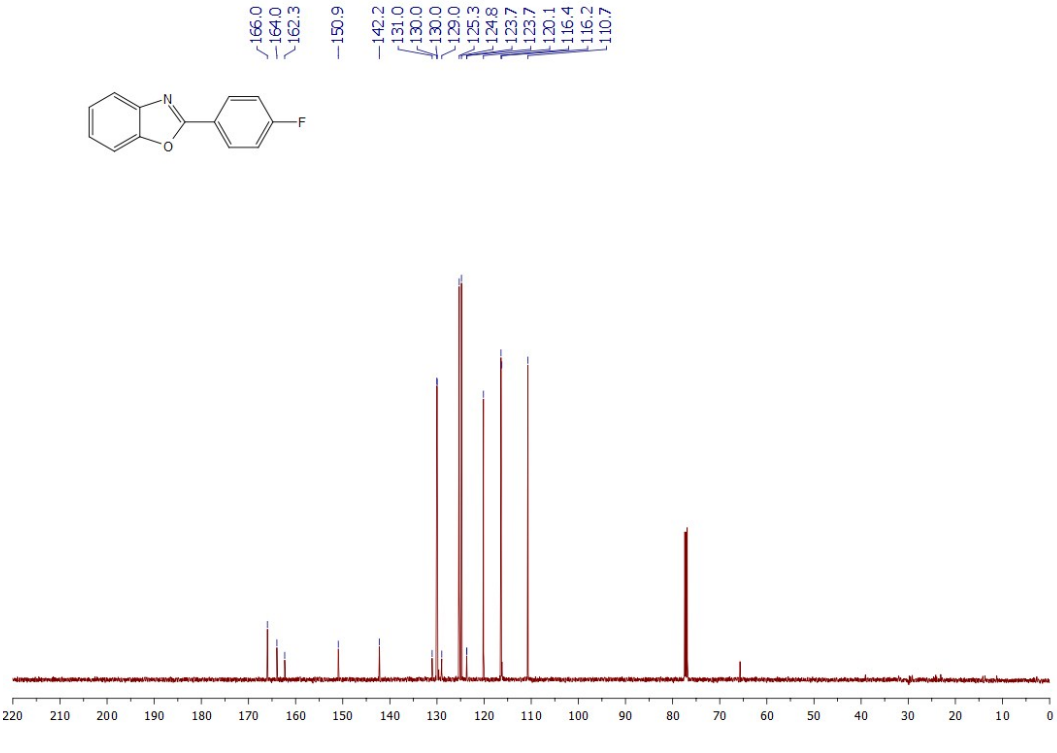

Figure S8. ^{1}H and ^{13}C NMR spectra of 2-(4-fluorophenyl)benzoxazole

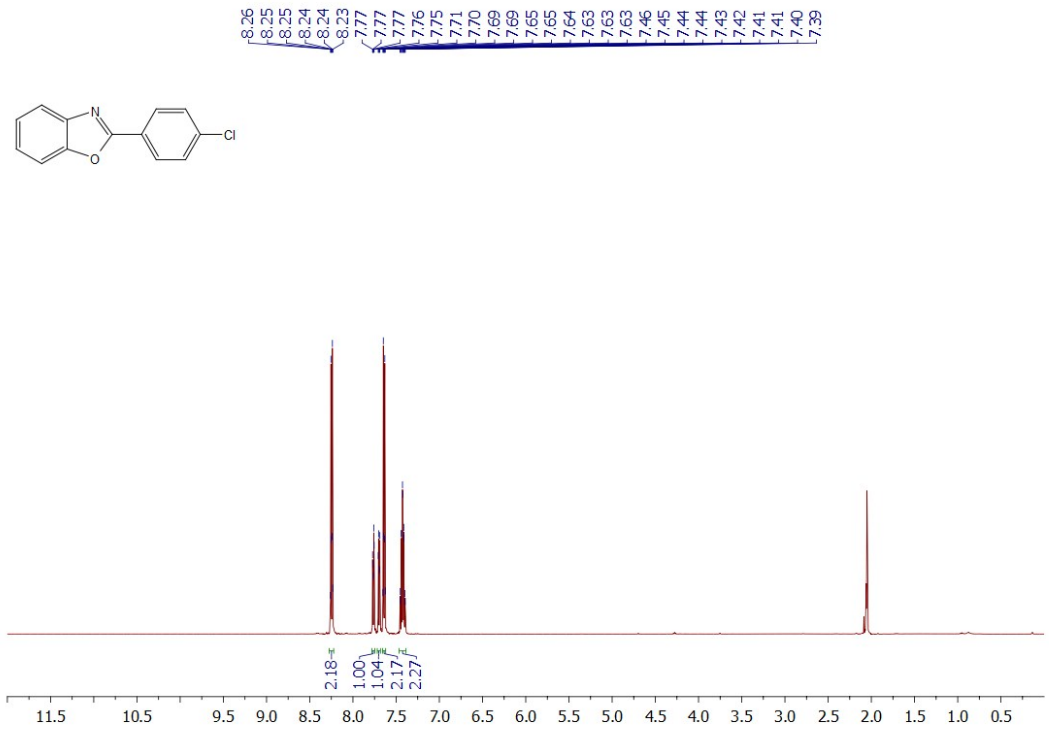

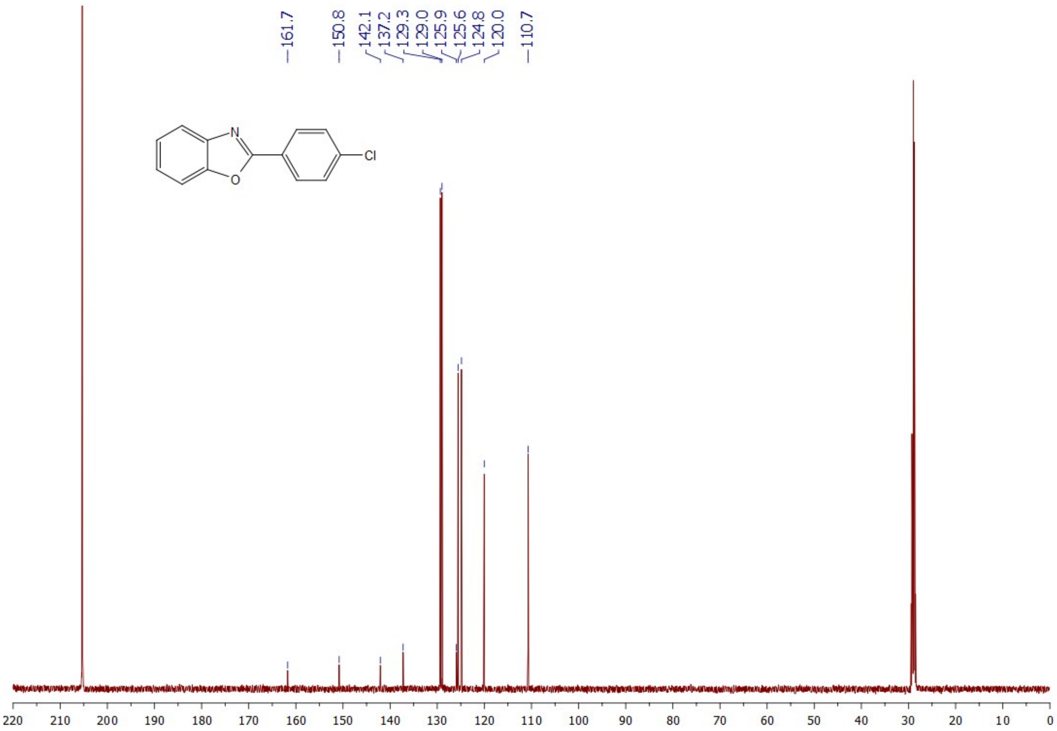

Figure S9. ^{1}H and ^{13}C NMR spectra of 2-(4-chlorophenyl)benzoxazole

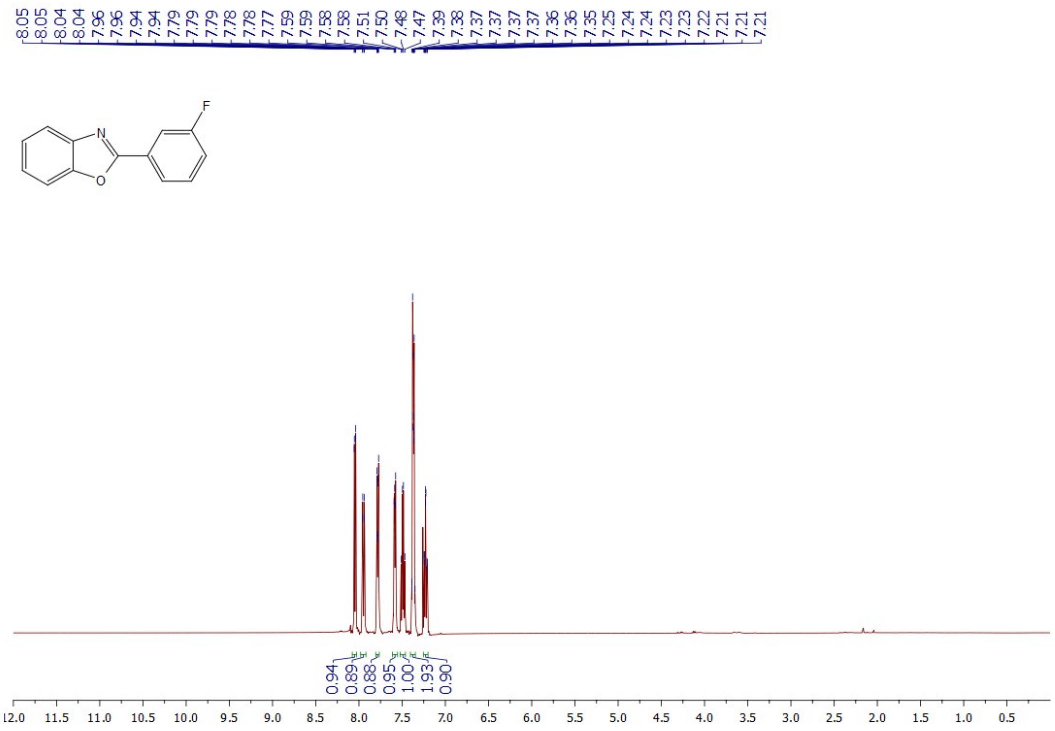

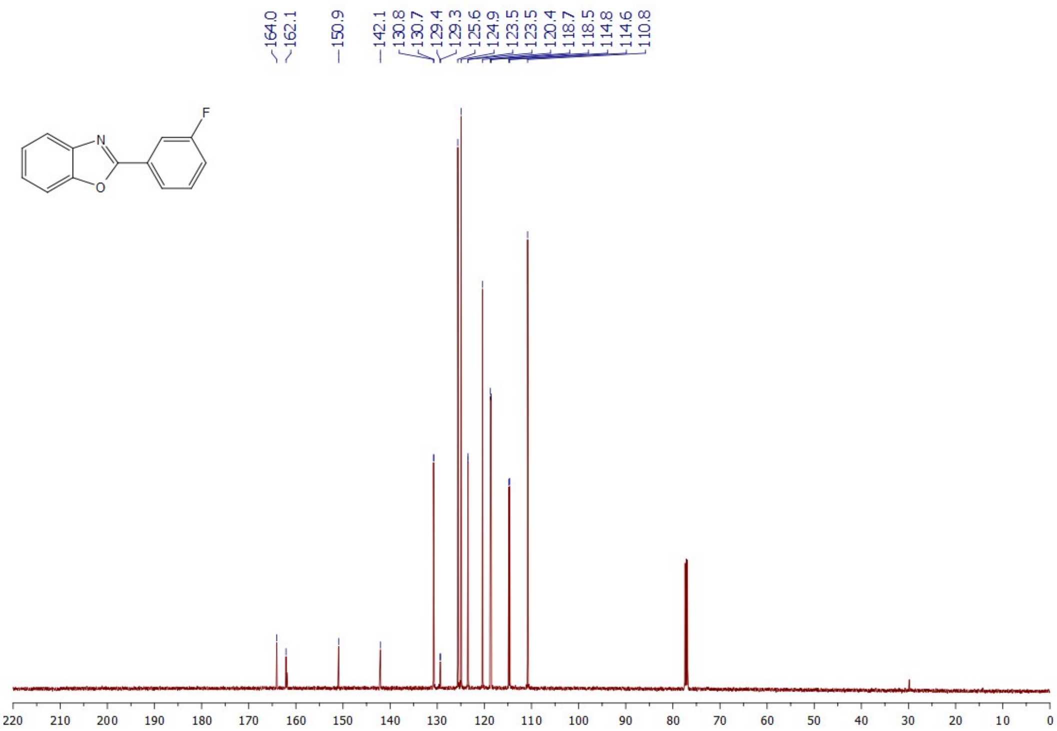

Figure S10. ^{1}H and ^{13}C NMR spectra of 2-(3-fluorophenyl)benzoxazole

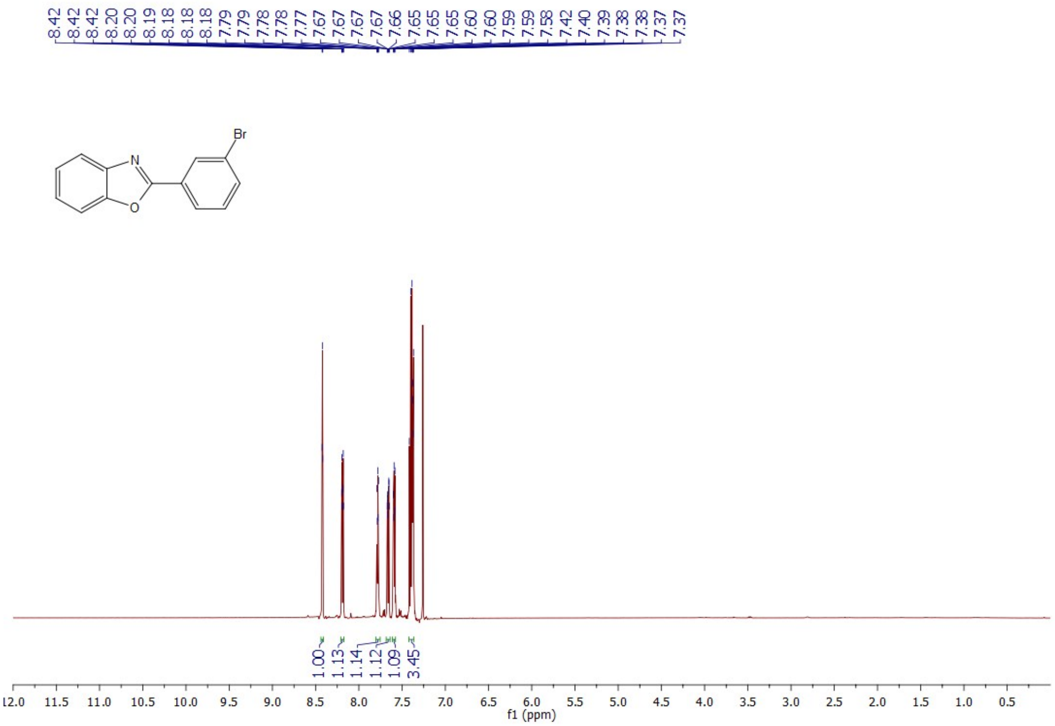

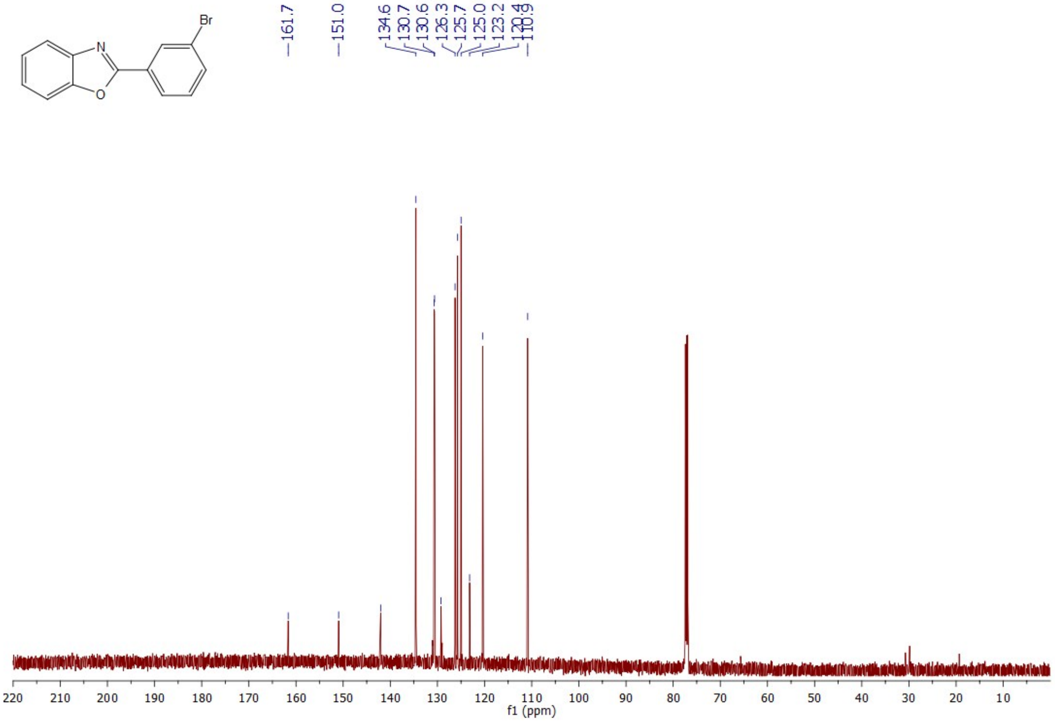

Figure S11. ^{1}H and ^{13}C NMR spectra of 2-(3-bromophenyl)benzoxazole

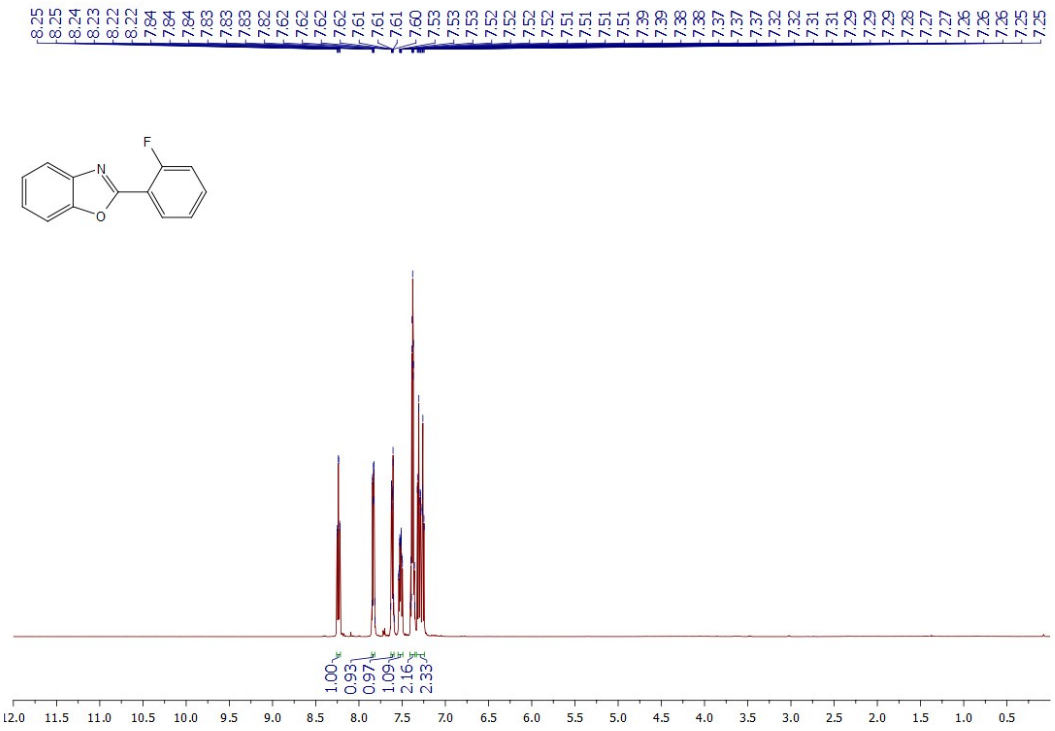

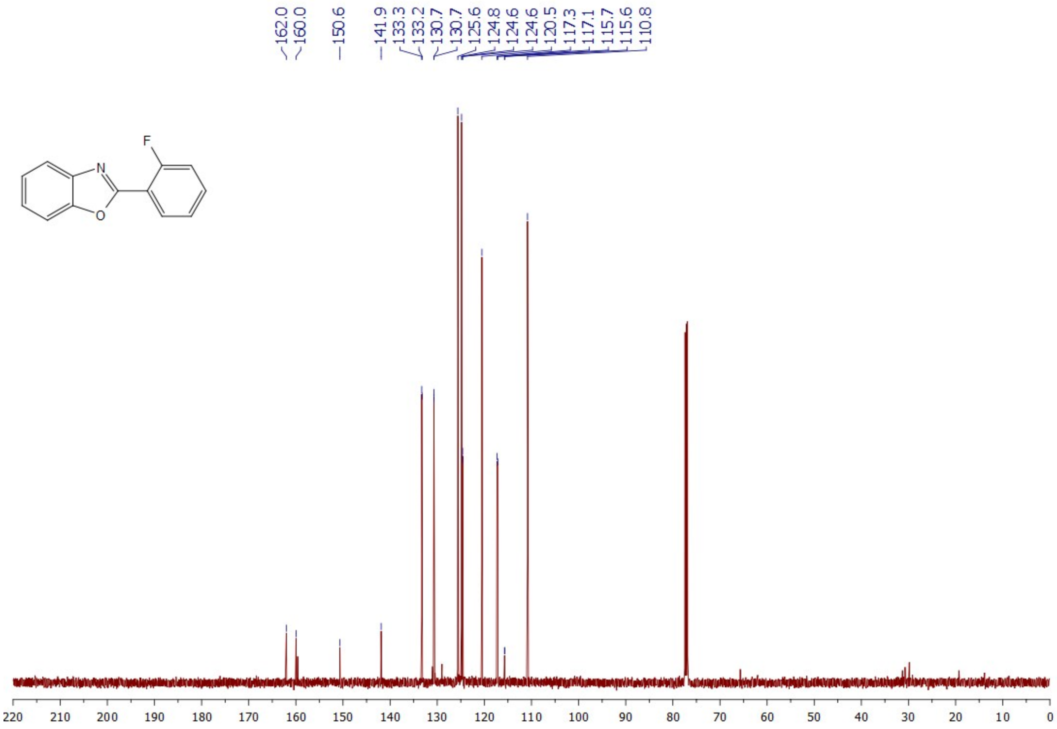

Figure S12. ^{1}H and ^{13}C NMR spectra of 2-(2-fluorophenyl)benzoxazole

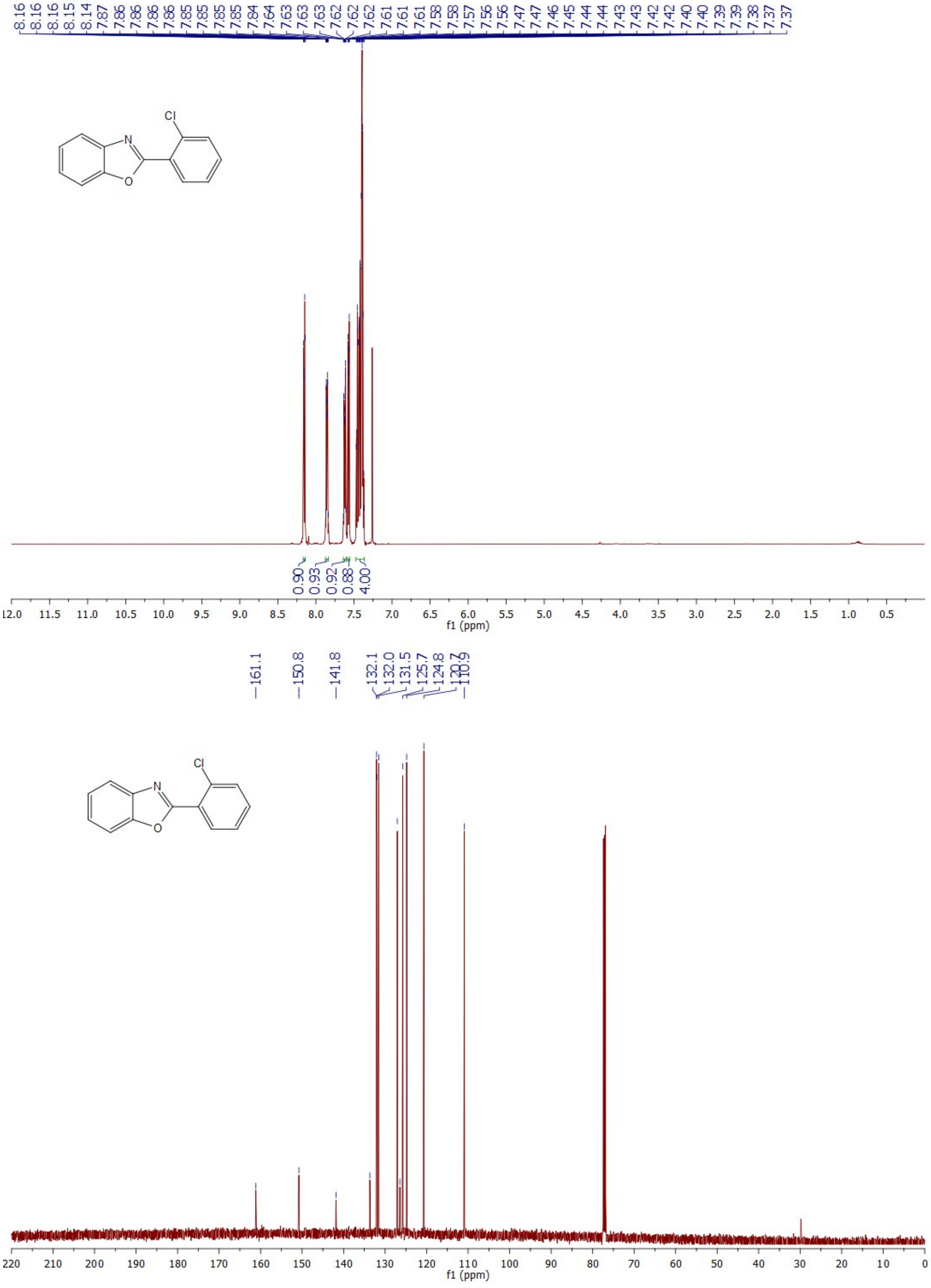

Figure S13. ^{1}H and ^{13}C NMR spectra of 2-(2-chlorophenyl)benzoxazole

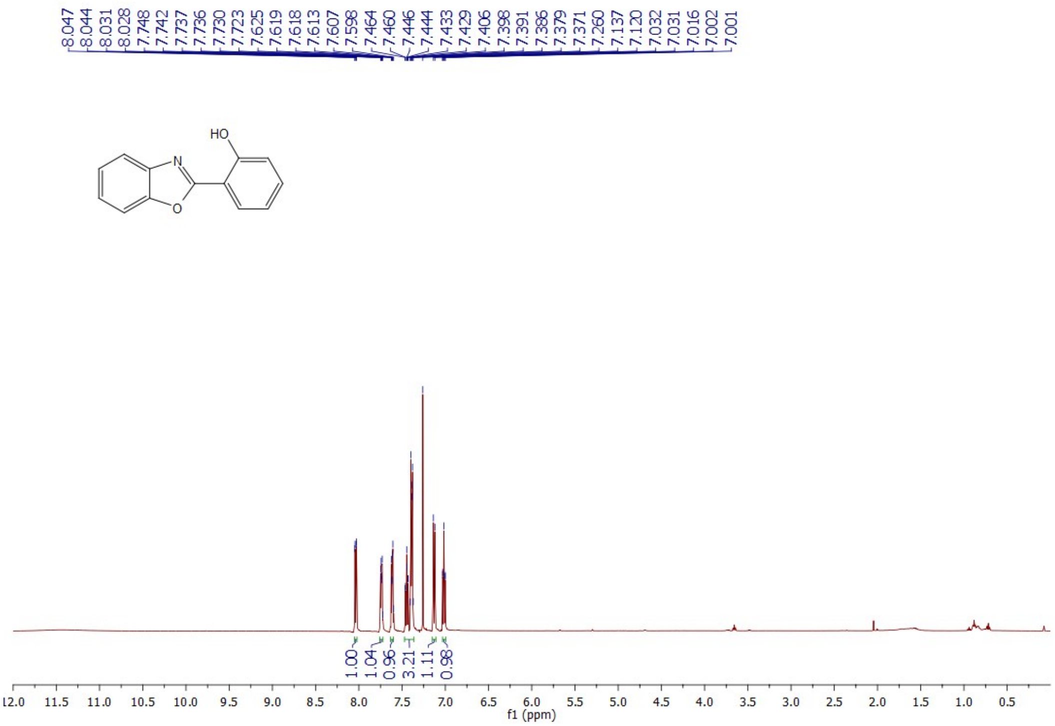

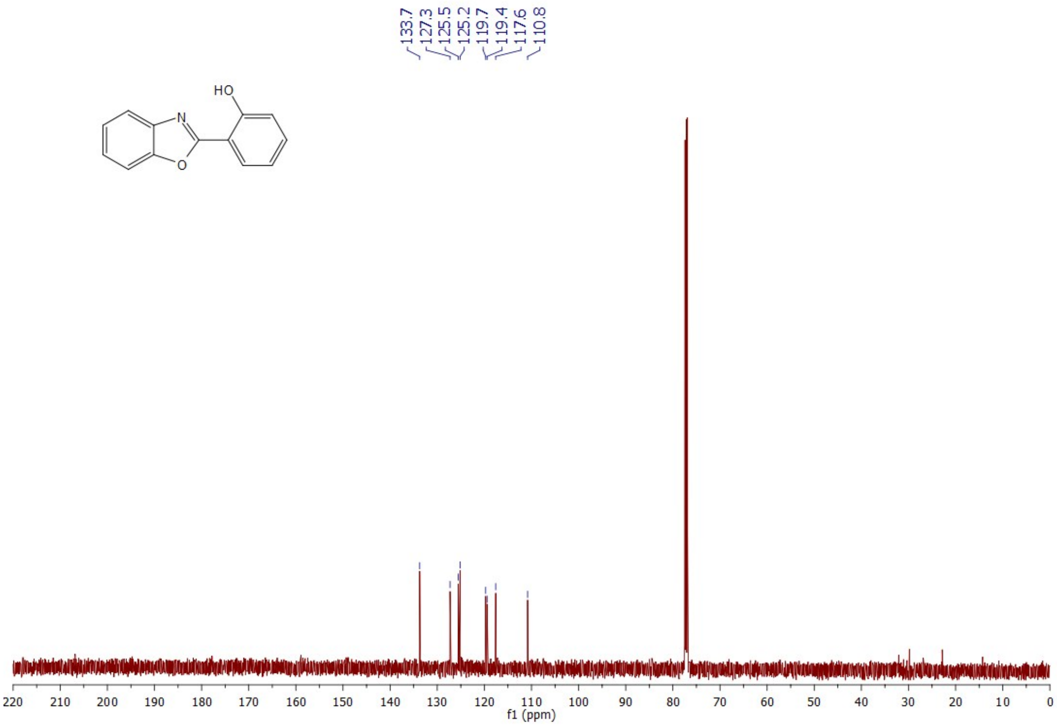

Figure S14. ^{1}H and ^{13}C NMR spectra of 2-(2-hydroxyphenyl)benzoxazole

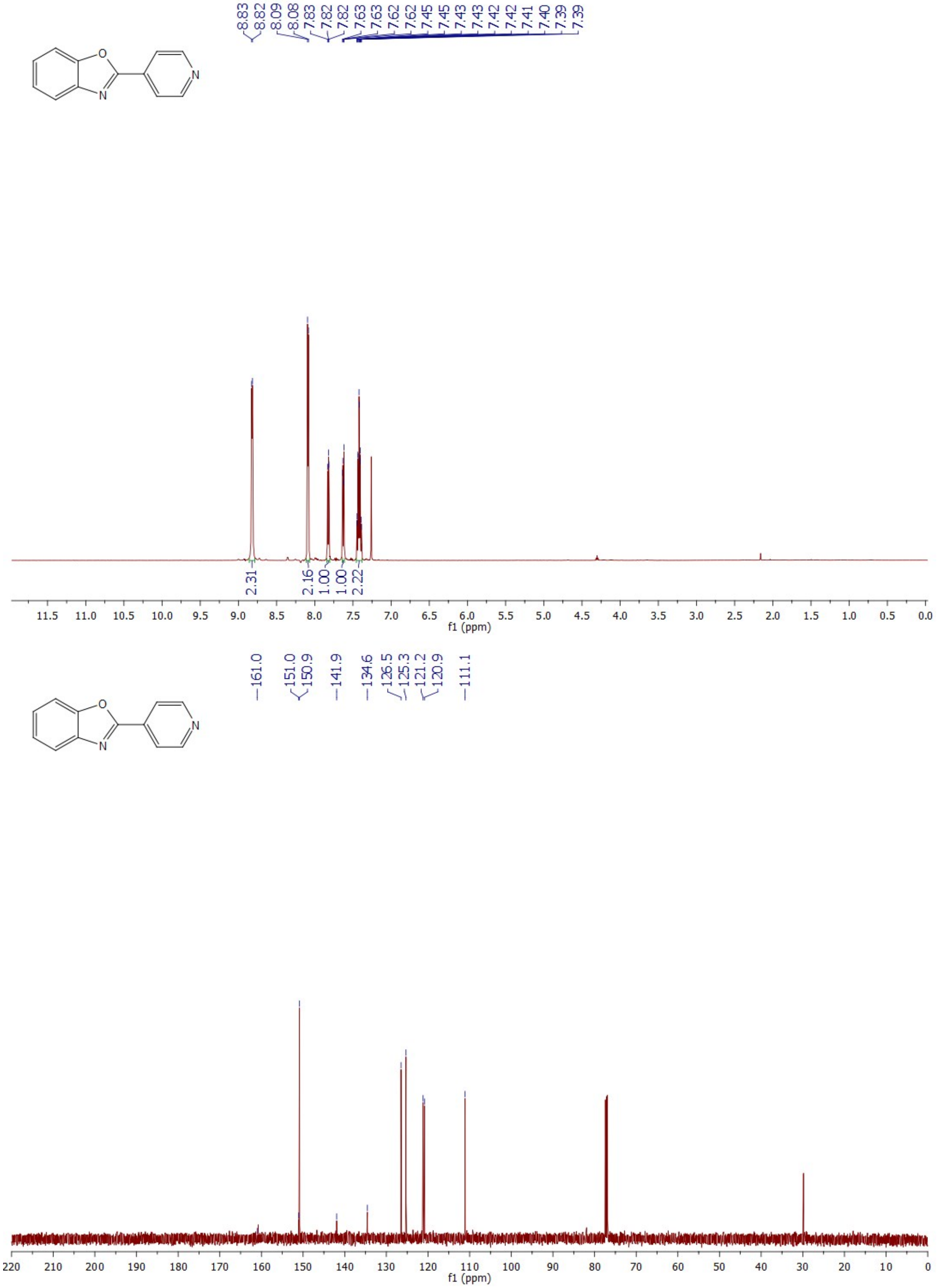

Figure S15. ^{1}H and ^{13}C NMR spectra of 2-(pyridine-4-yl)benzoxazole

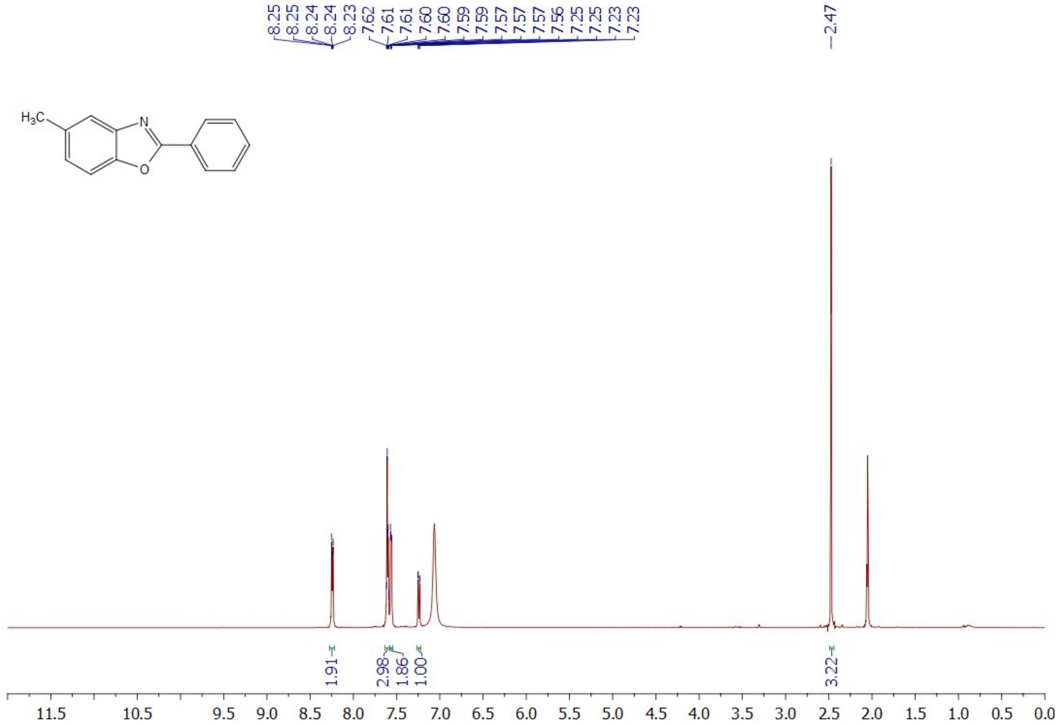

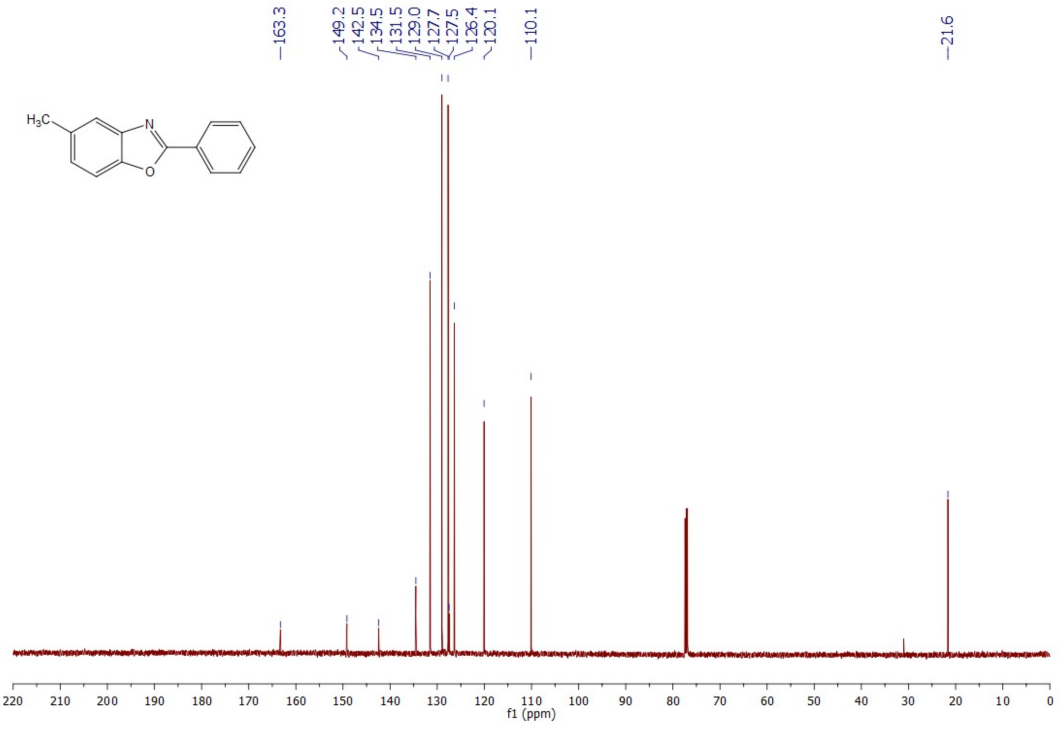

Figure S16. ^{1}H and ^{13}C NMR spectra of 5-methyl-2-phenylbenzoxazole

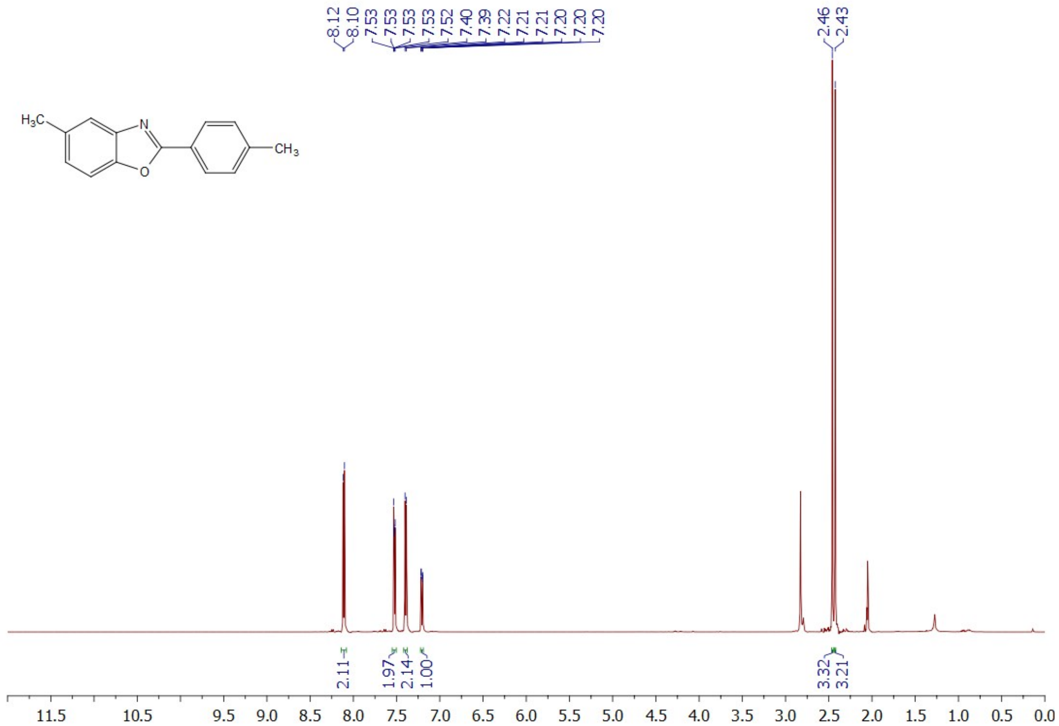

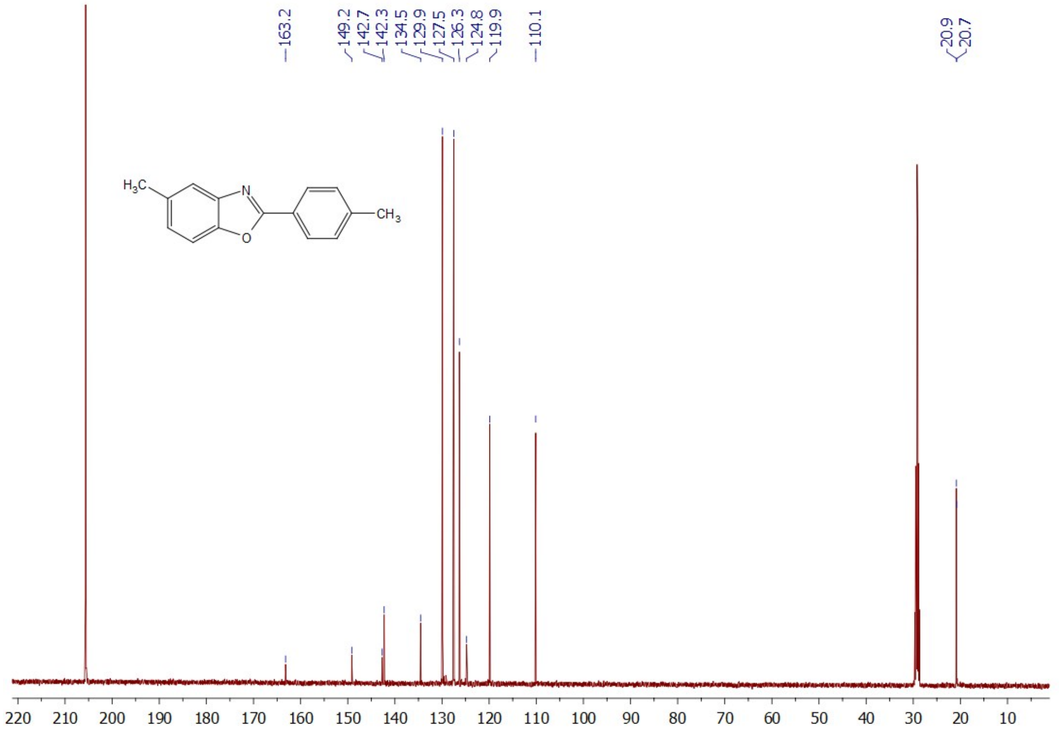

Figure S17. ^{1}H and ^{13}C NMR spectra of 5-methyl-2-(*p*-tolyl)benzoxazole

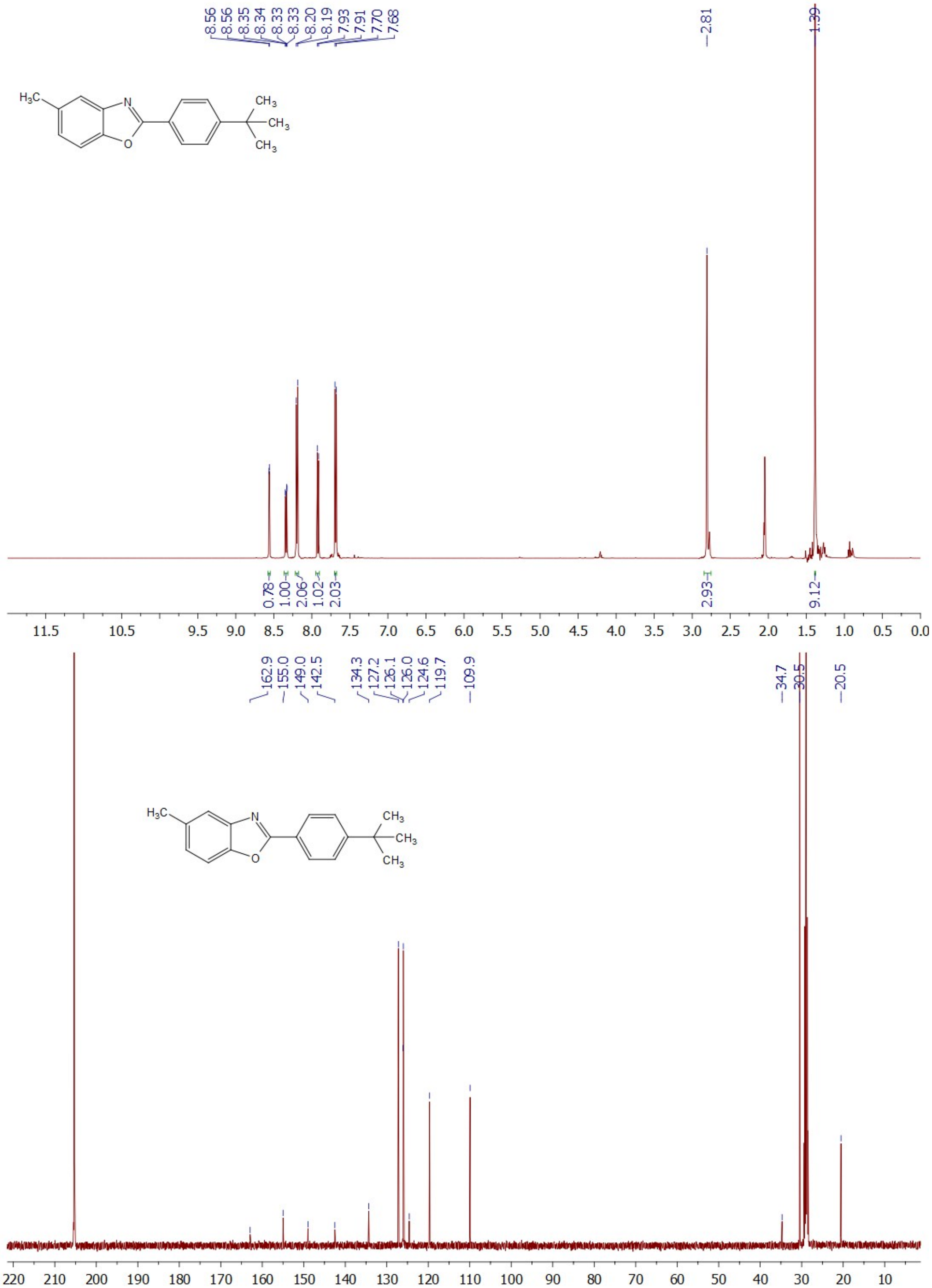

Figure S18. ^{1}H and ^{13}C NMR spectra of 5-methyl-2-(4-*tert*-butylphenyl)benzoxazole

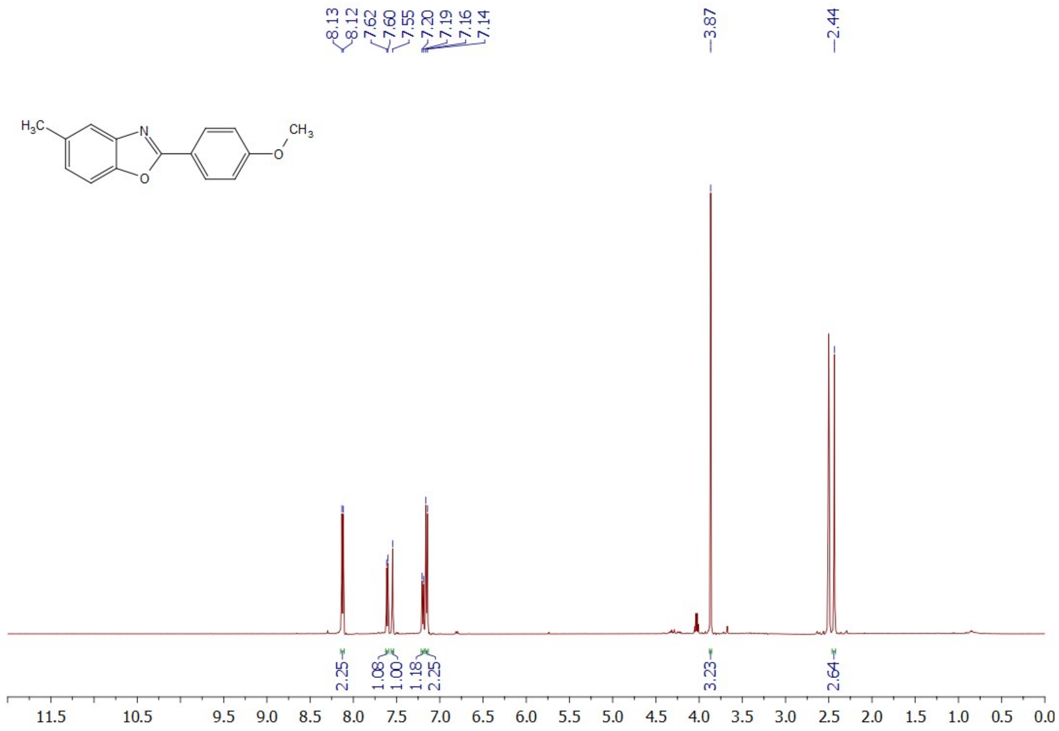

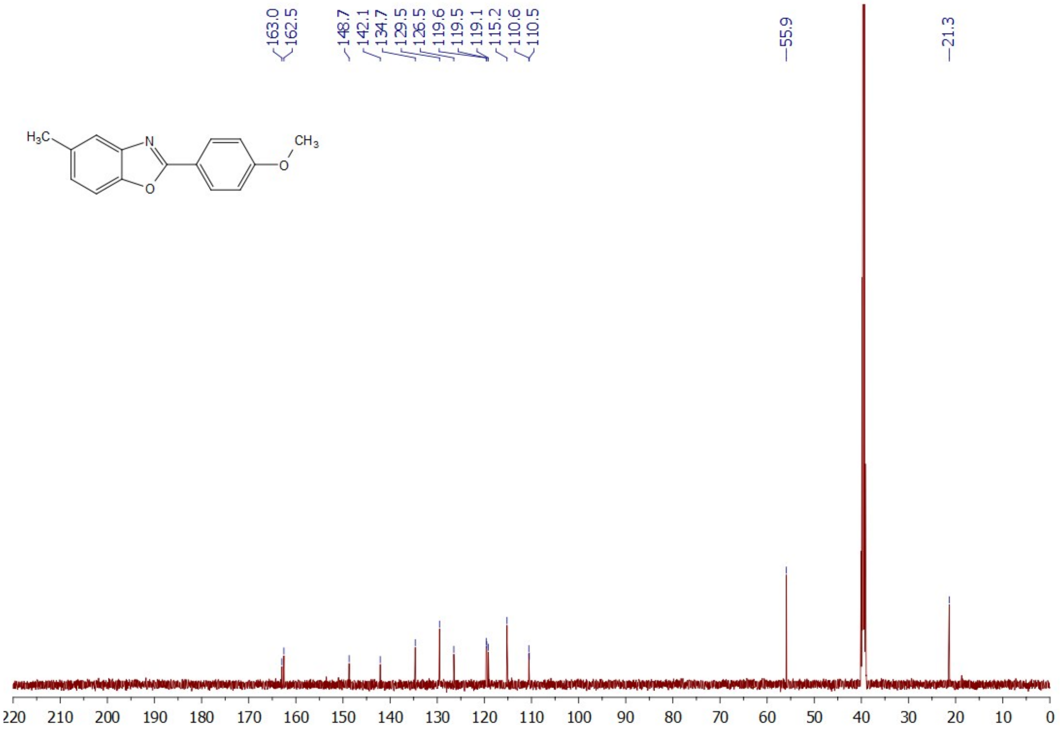

Figure S19. ^{1}H and ^{13}C NMR spectra of 5-methyl-2-(4-methoxyphenyl)benzoxazole

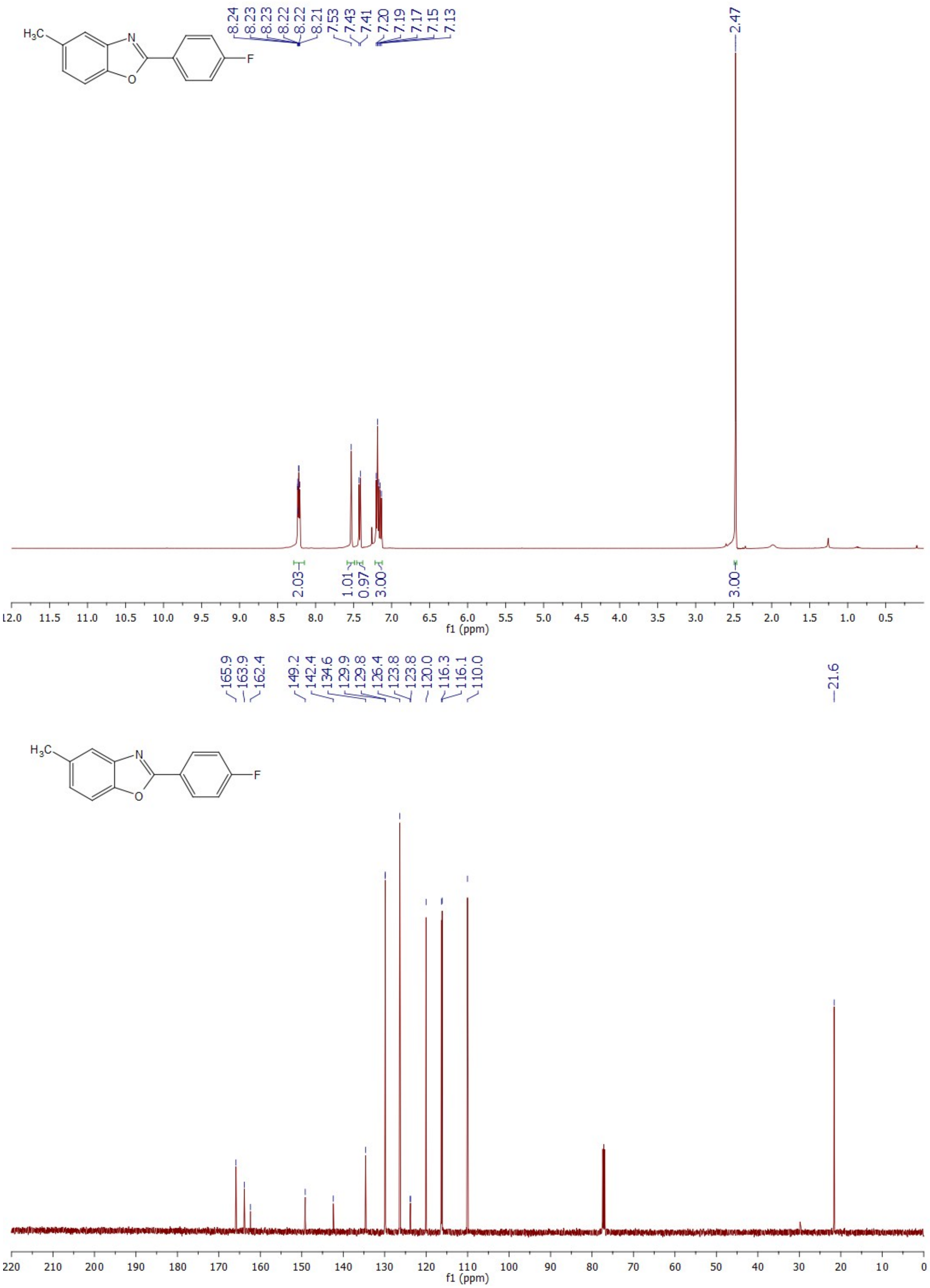

Figure S20. ^{1}H and ^{13}C NMR spectra of 5-methyl-2-(4-fluorophenyl)benzoxazole

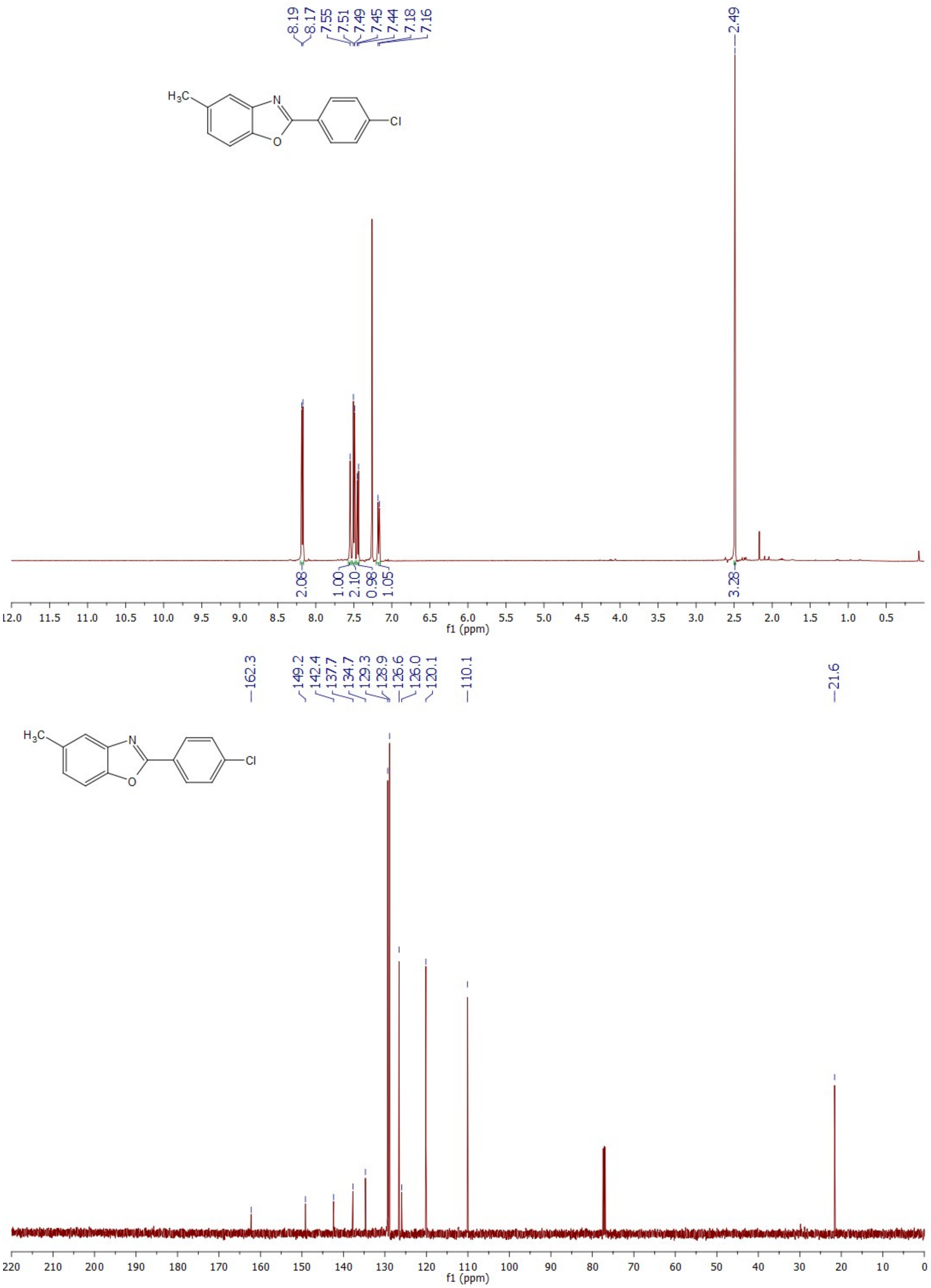

Figure S21. ^{1}H and ^{13}C NMR spectra of 5-methyl-2-(4-chlorophenyl)benzoxazole

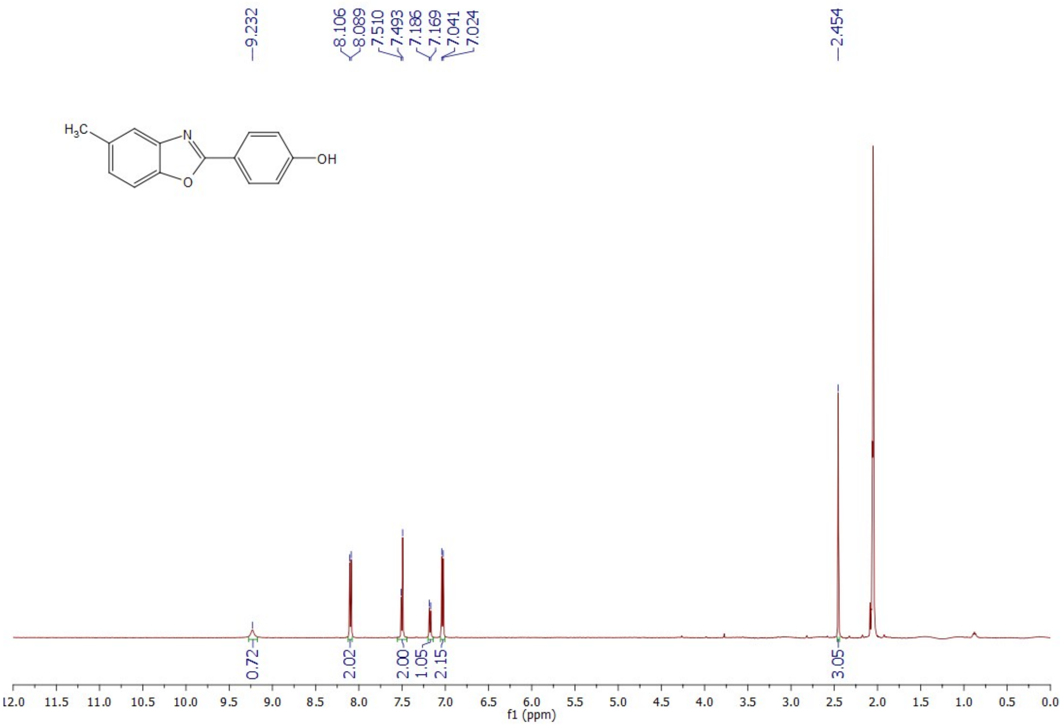

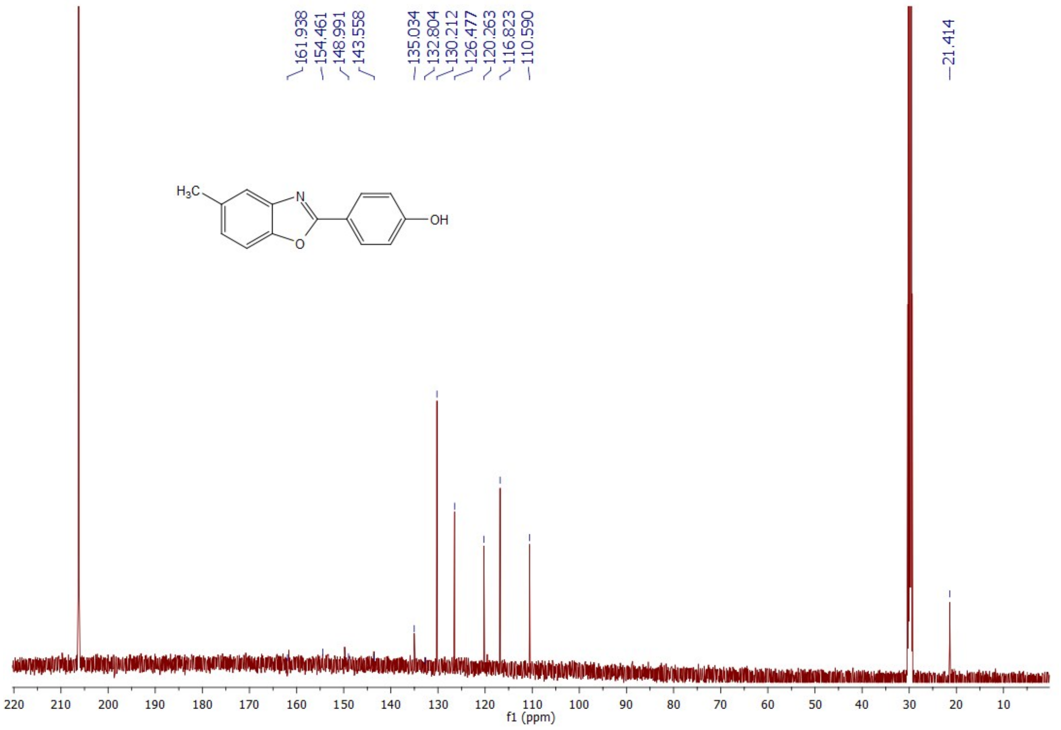

Figure S22. ^{1}H and ^{13}C NMR spectra of 5-methyl-2-(4-hydroxyphenyl)benzoxazole

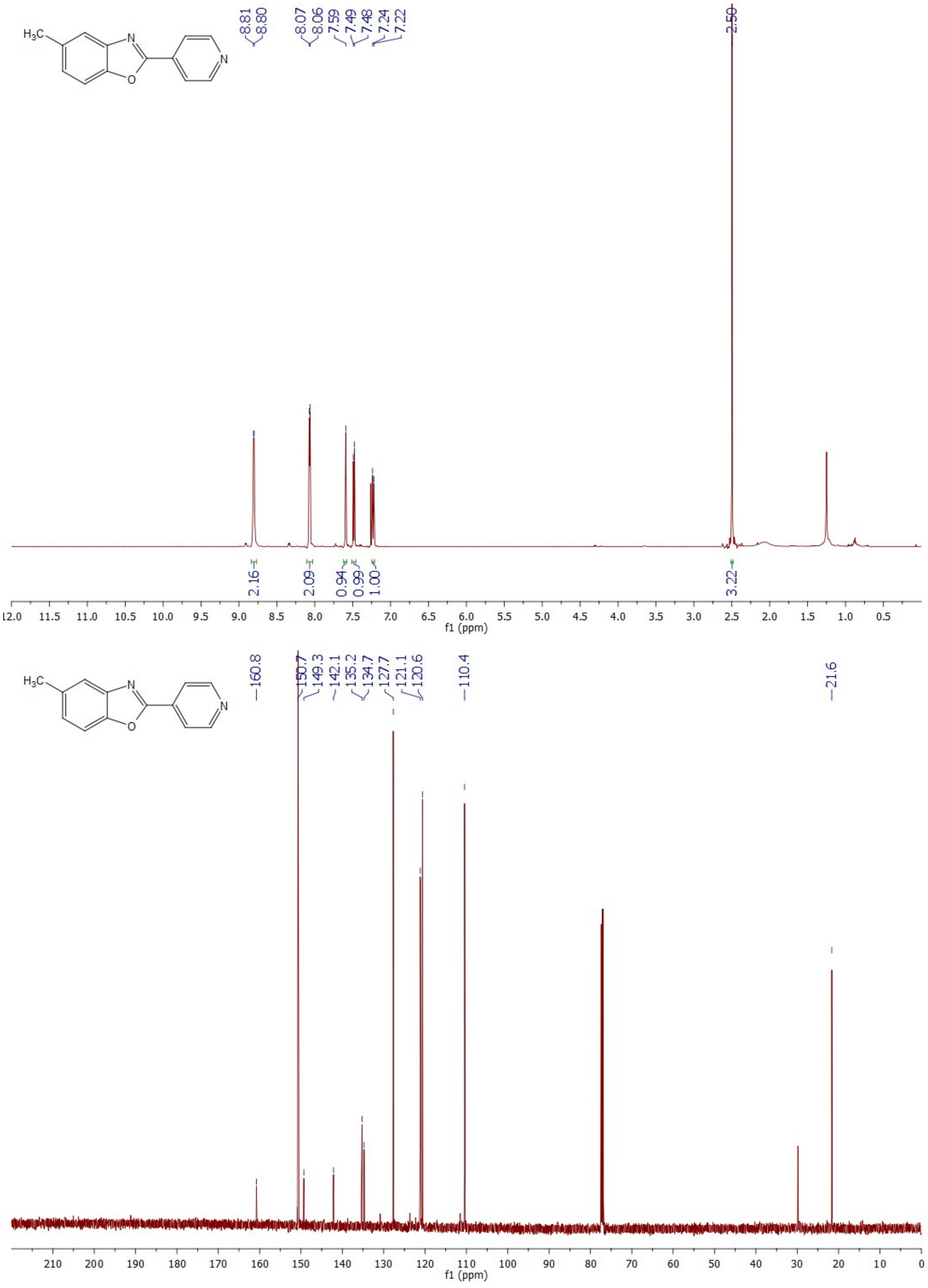

Figure S23. ^{1}H and ^{13}C NMR spectra of 5-methyl-2-(pyridine-4-yl)benzoxazole

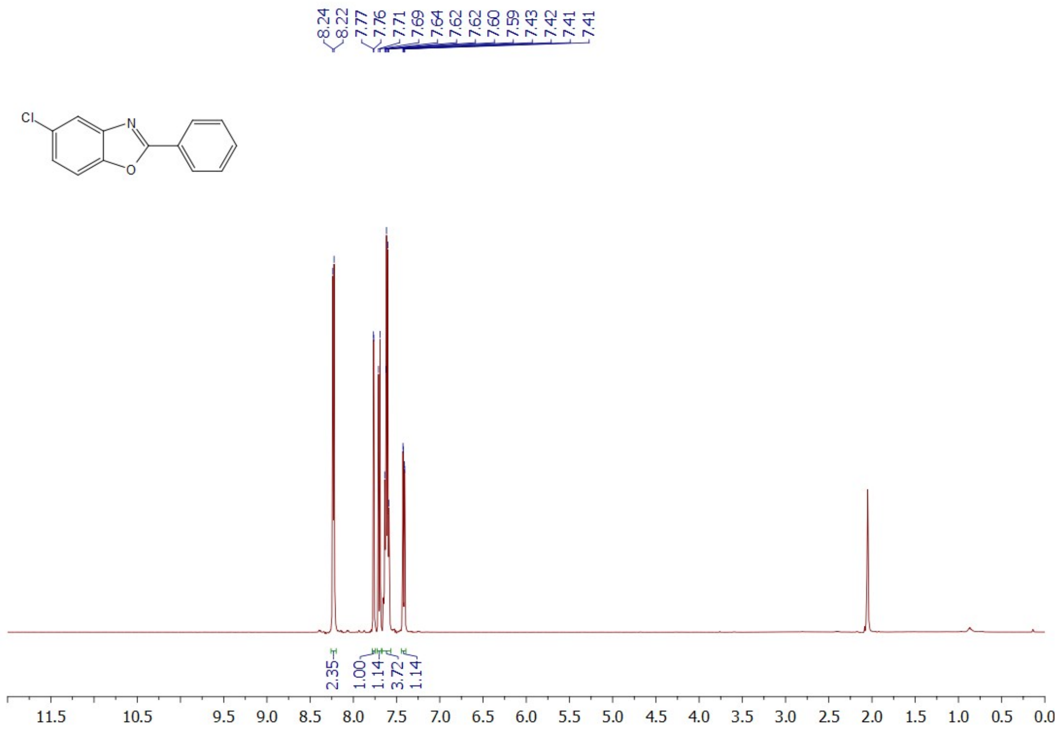

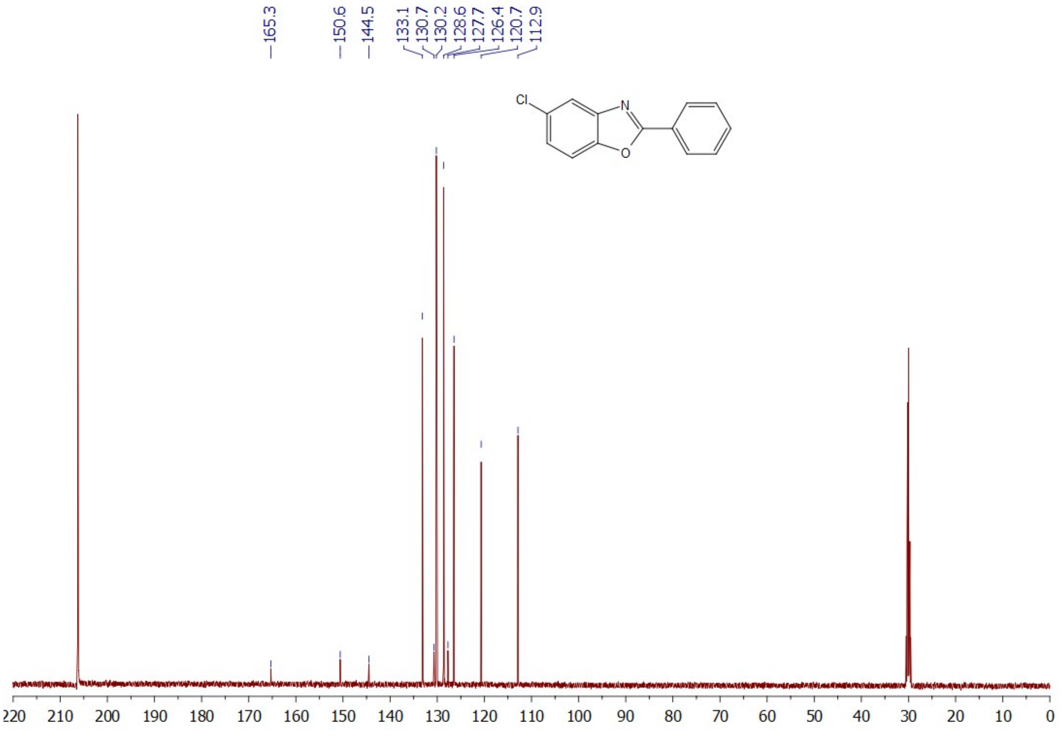

Figure S24. ^{1}H and ^{13}C NMR spectra of 5-chloro-2-phenylbenzoxazole

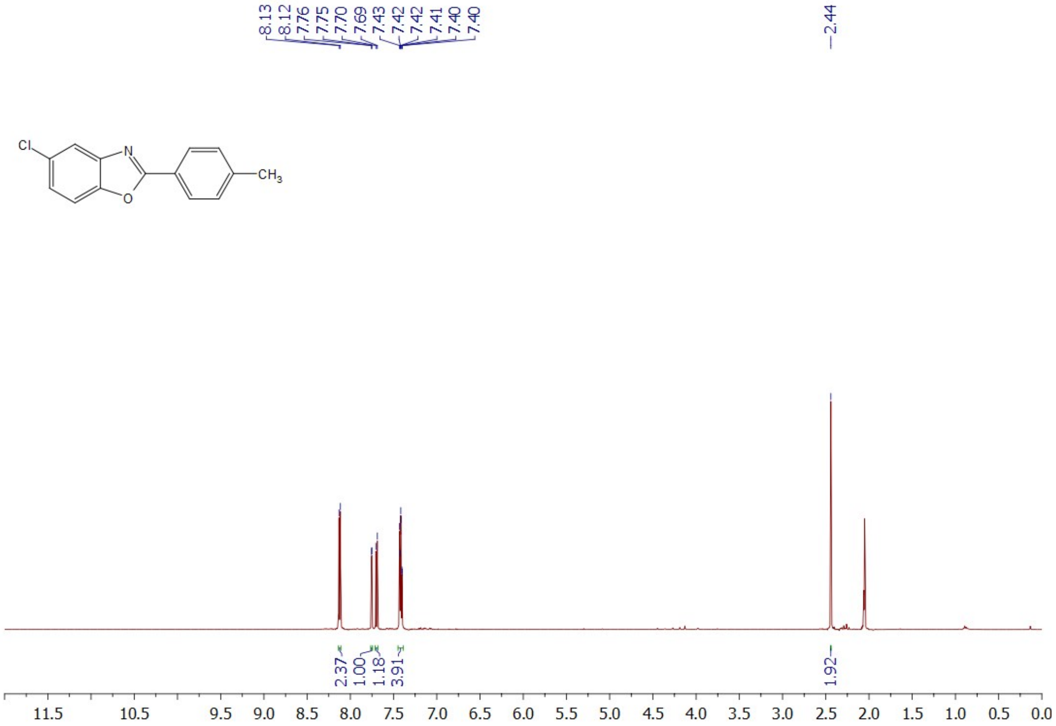

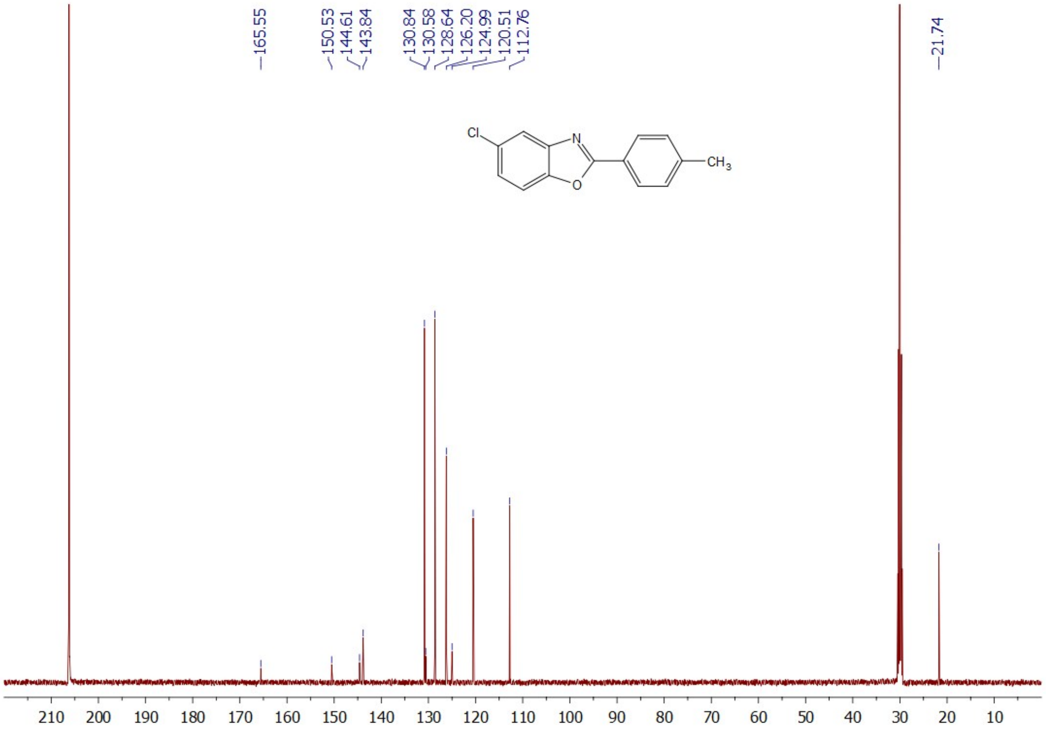

Figure S25. ^{1}H and ^{13}C NMR spectra of 5-chloro-2-(*p*-tolyl)benzoxazole

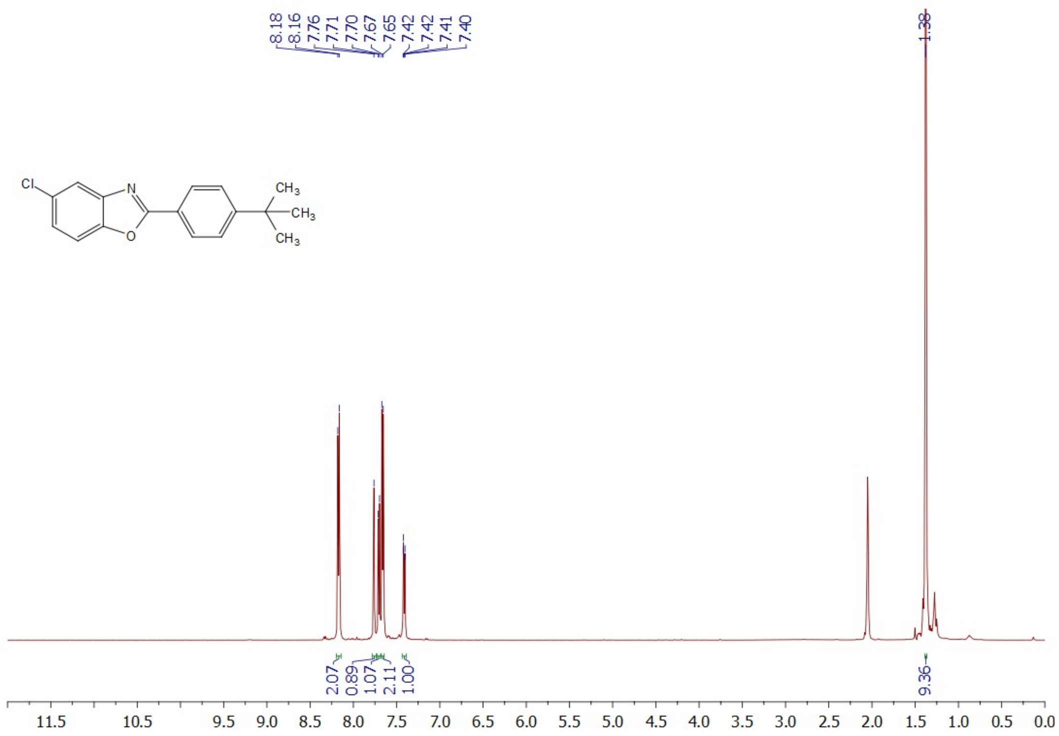

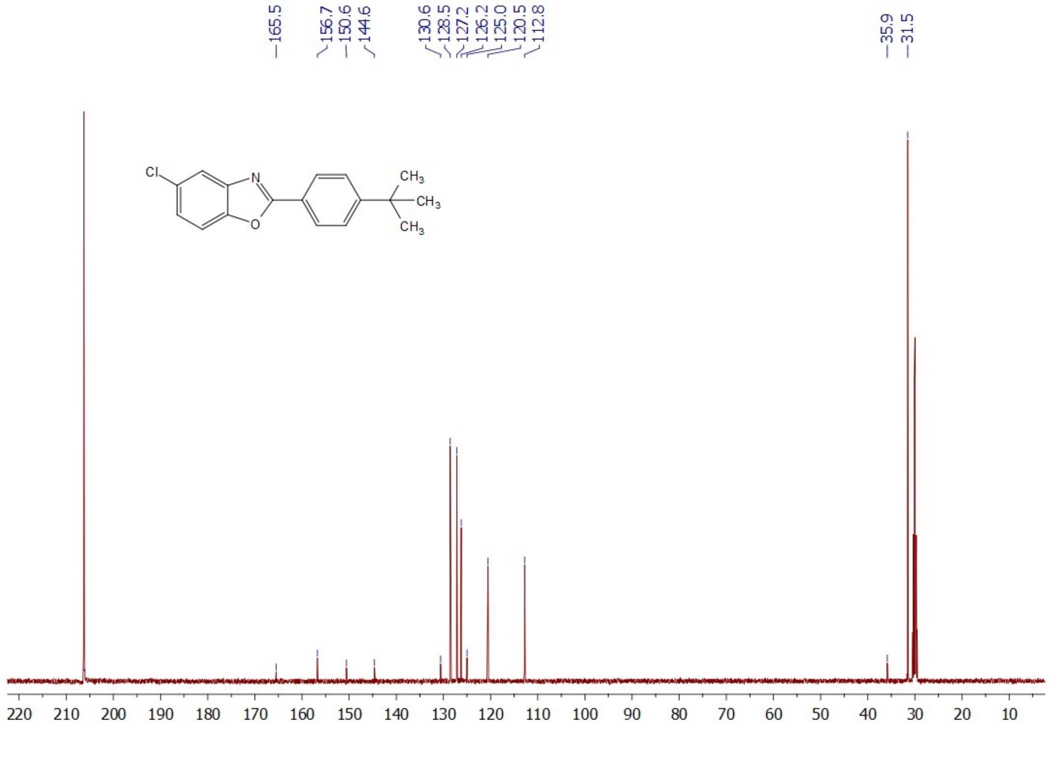

Figure S26. ^{1}H and ^{13}C NMR spectra of 5-chloro-2-(4-*tert*-butylphenyl)benzoxazole

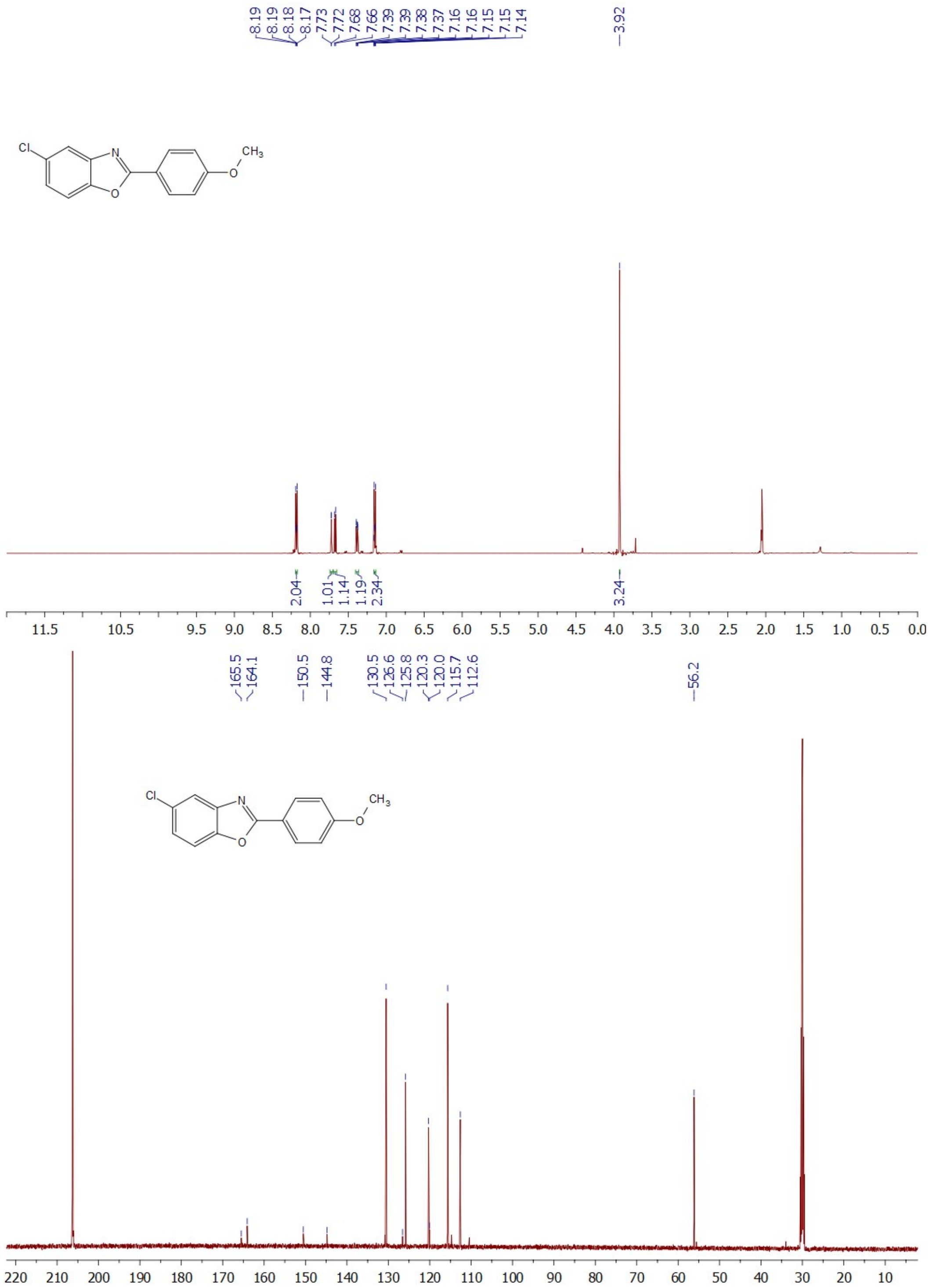

Figure S27. ^{1}H and ^{13}C NMR spectra of 5-chloro-2-(4-methoxyphenyl)benzoxazole

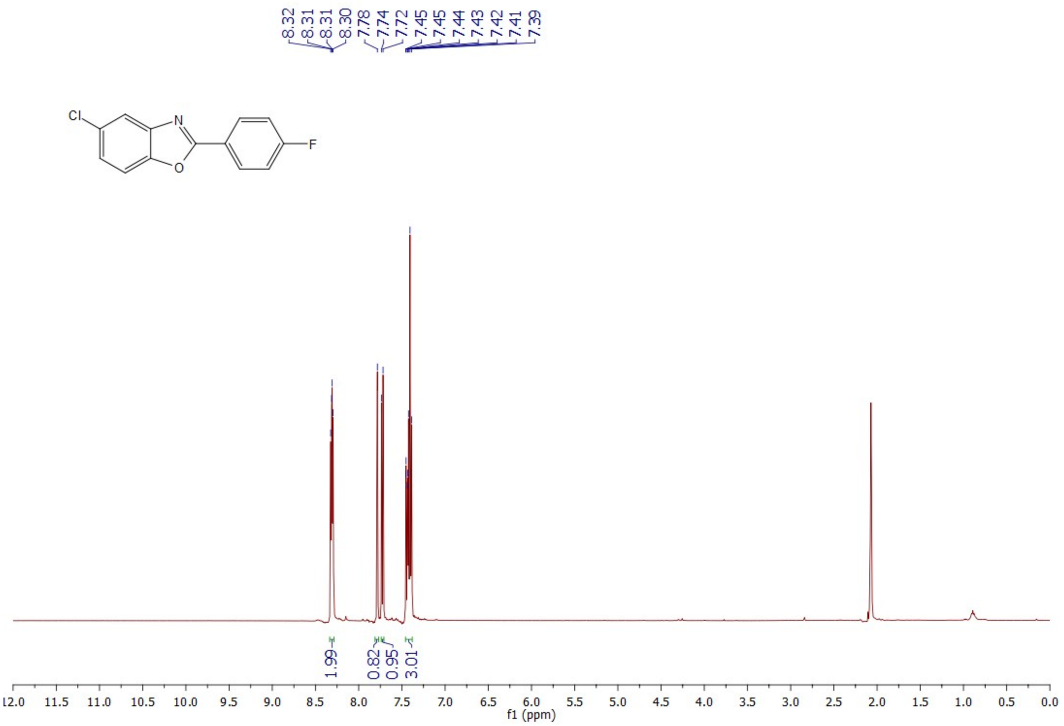

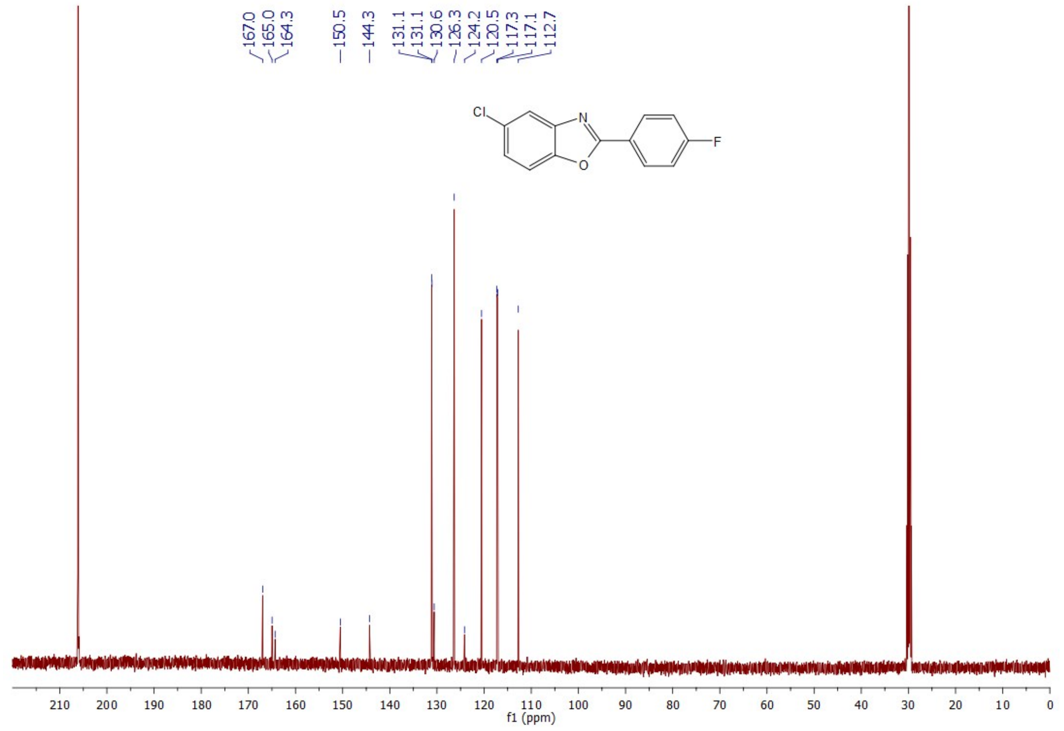

Figure S28. ^{1}H and ^{13}C NMR spectra of 5-chloro-2-(4-fluorophenyl)benzoxazole

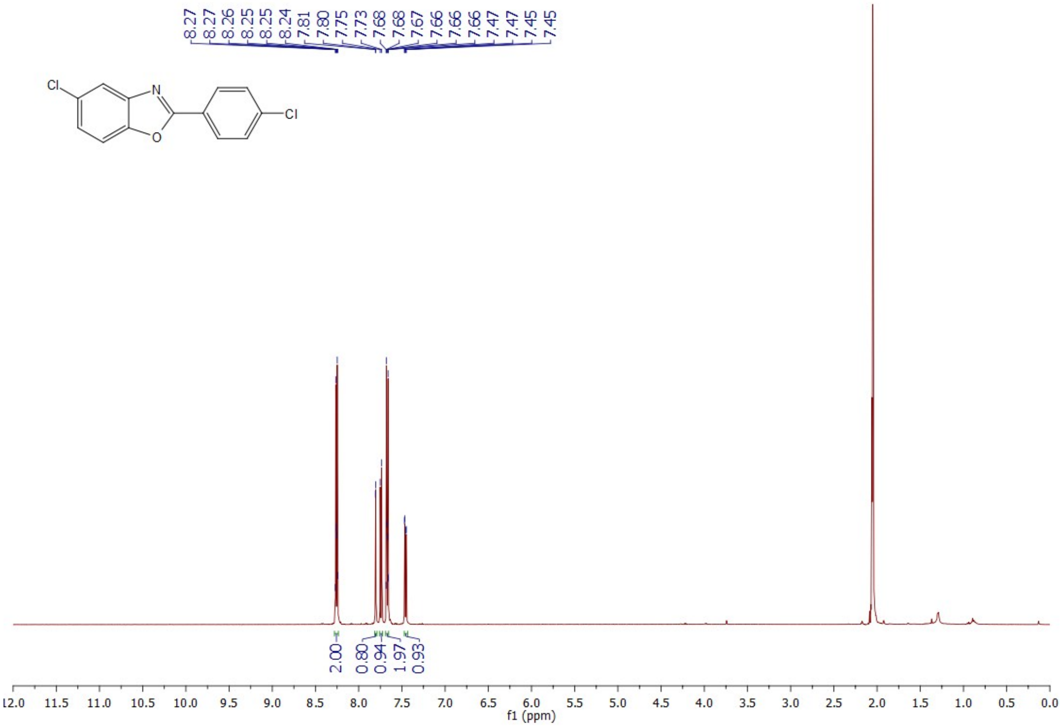

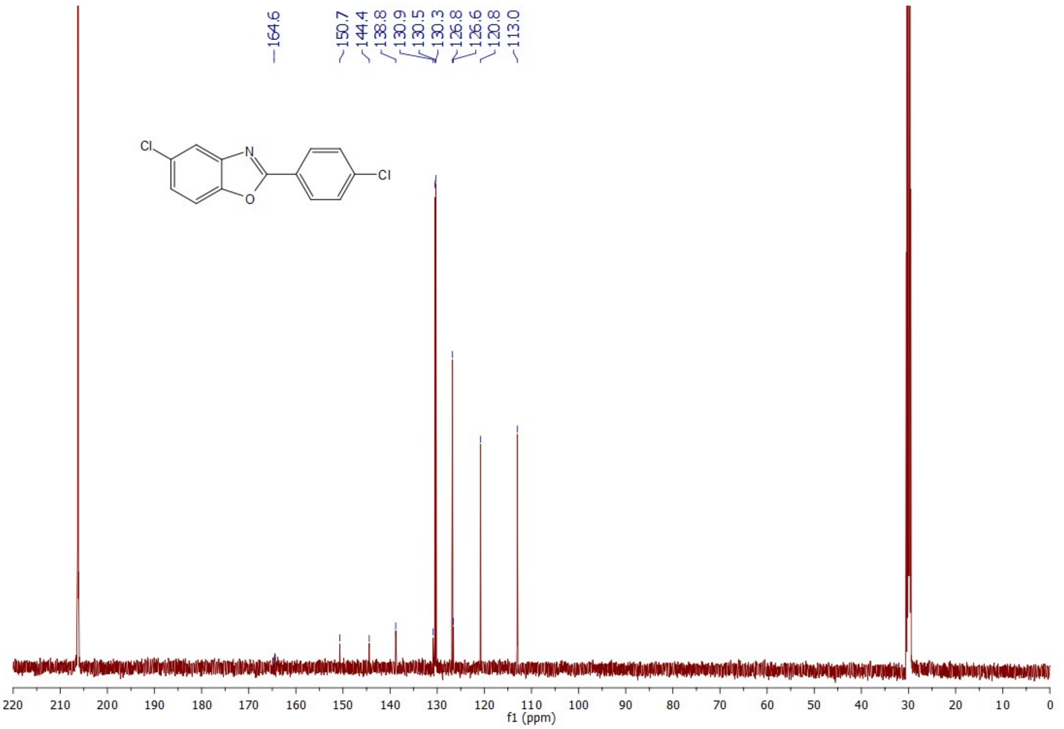

Figure S29. ^{1}H and ^{13}C NMR spectra 5-chloro-2-(4-chlorophenyl)benzoxazole

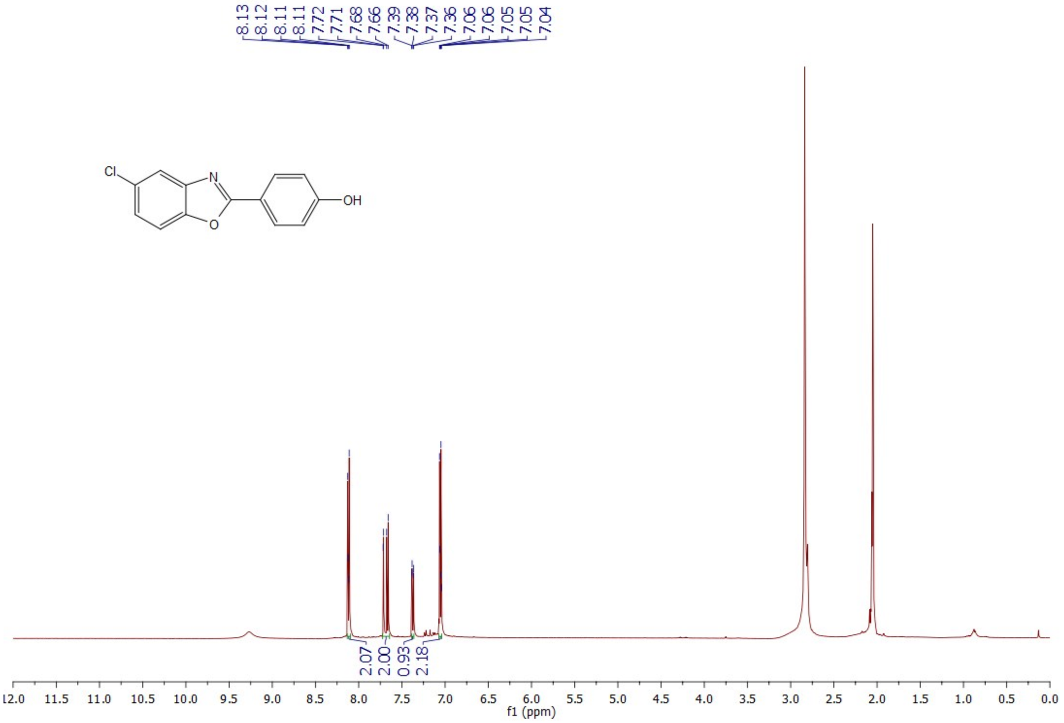

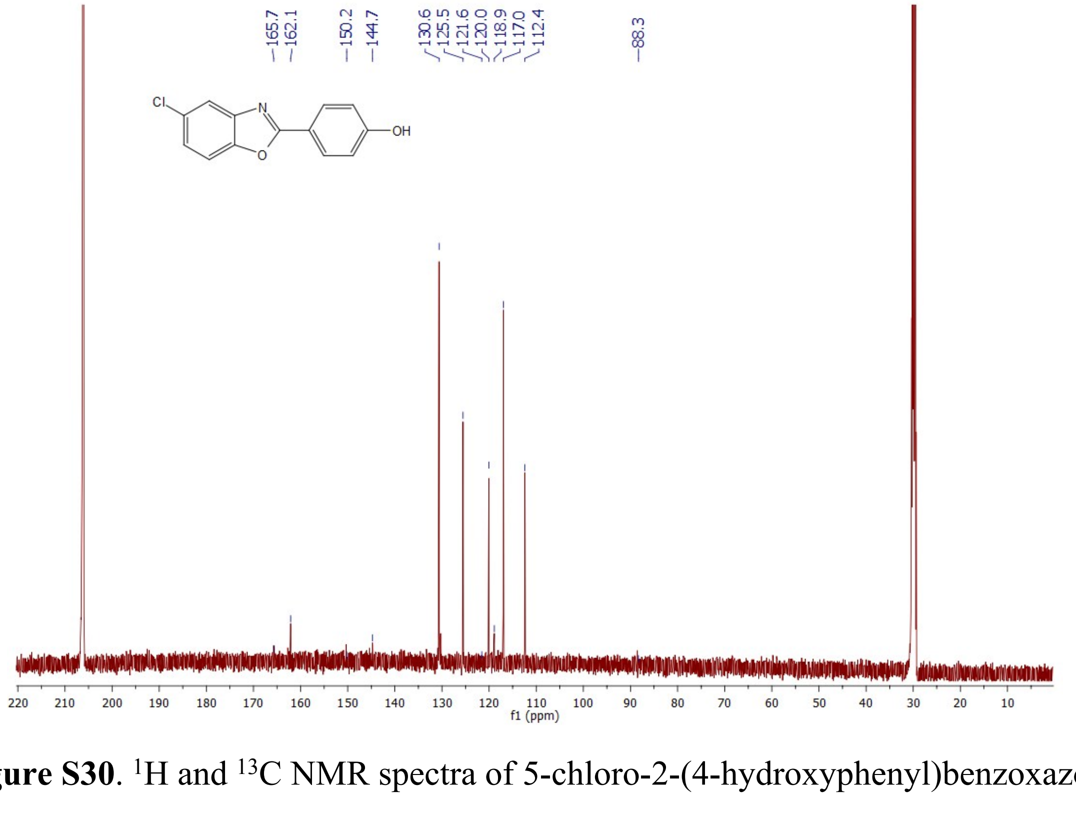

Figure S30. ^{1}H and ^{13}C NMR spectra of 5-chloro-2-(4-hydroxyphenyl)benzoxazole

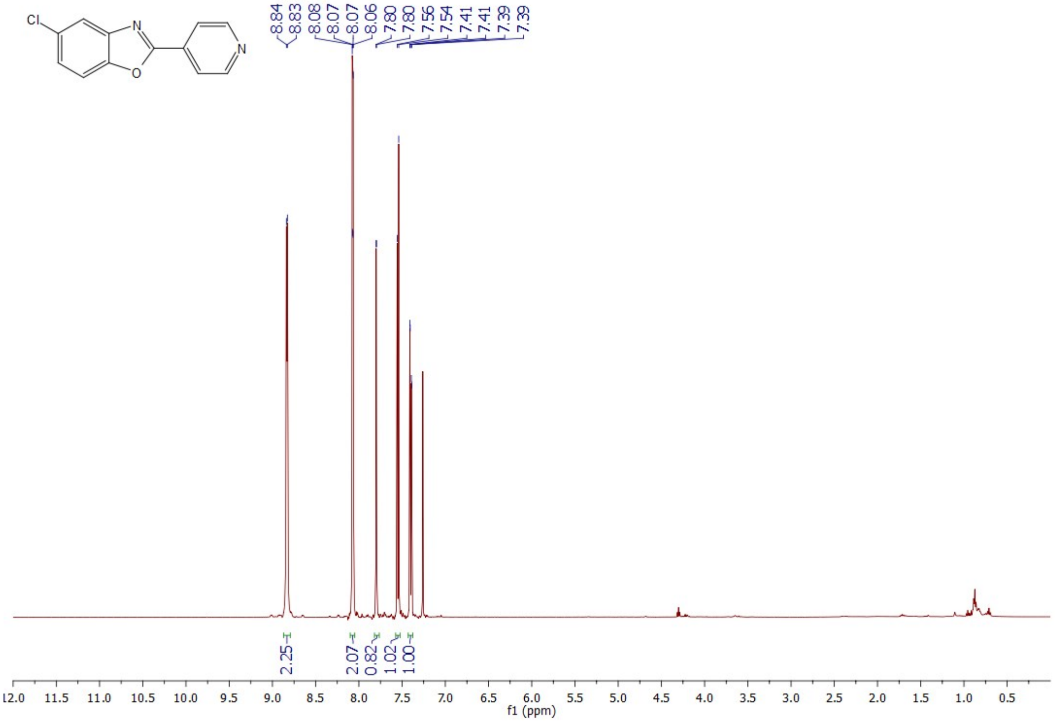

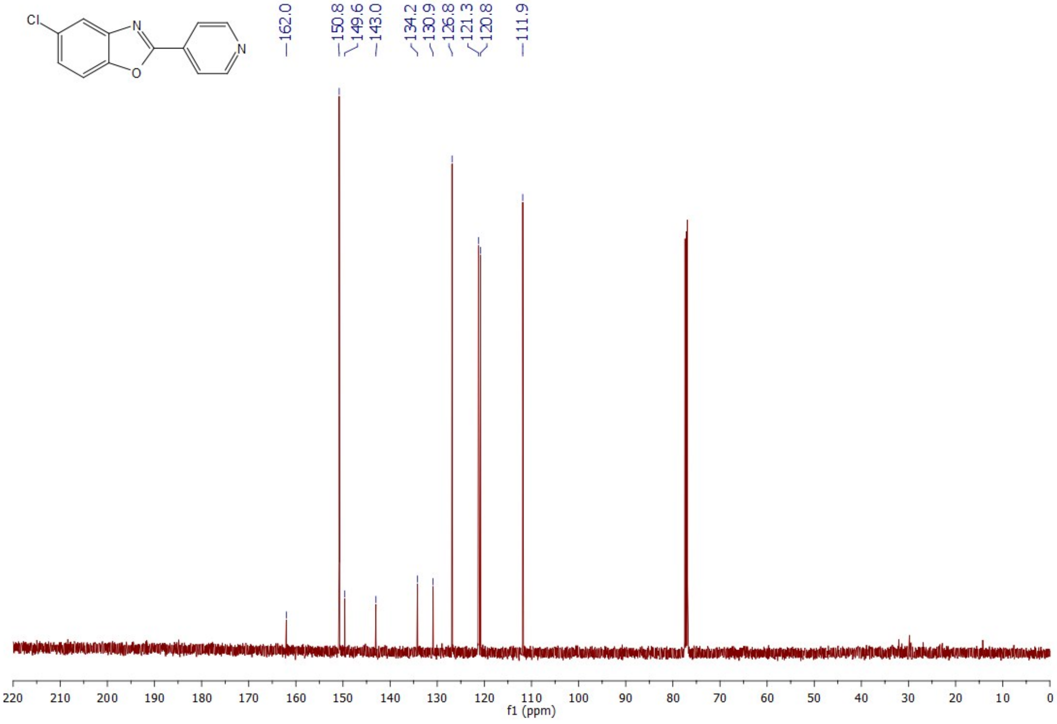

Figure S31. ^{1}H and ^{13}C NMR spectra of 5-chloro-2-(pyridin-4-yl)benzoxazole

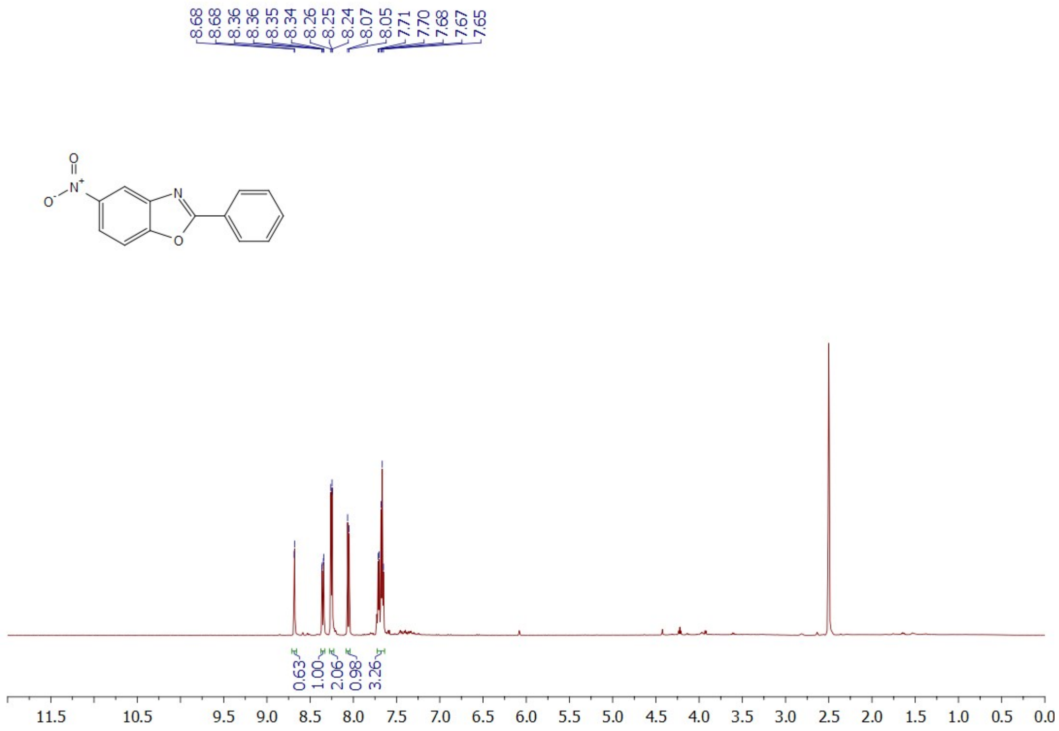

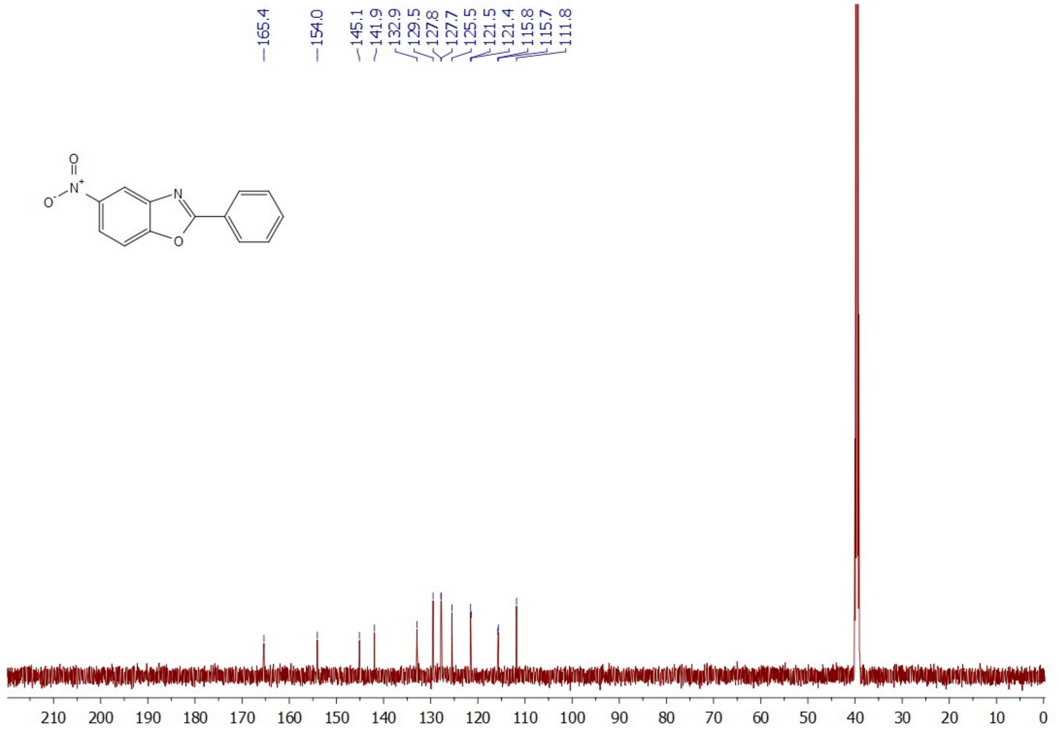

Figure S32. ^{1}H and ^{13}C NMR spectra of 5-nitro-2-phenylbenzoxazole

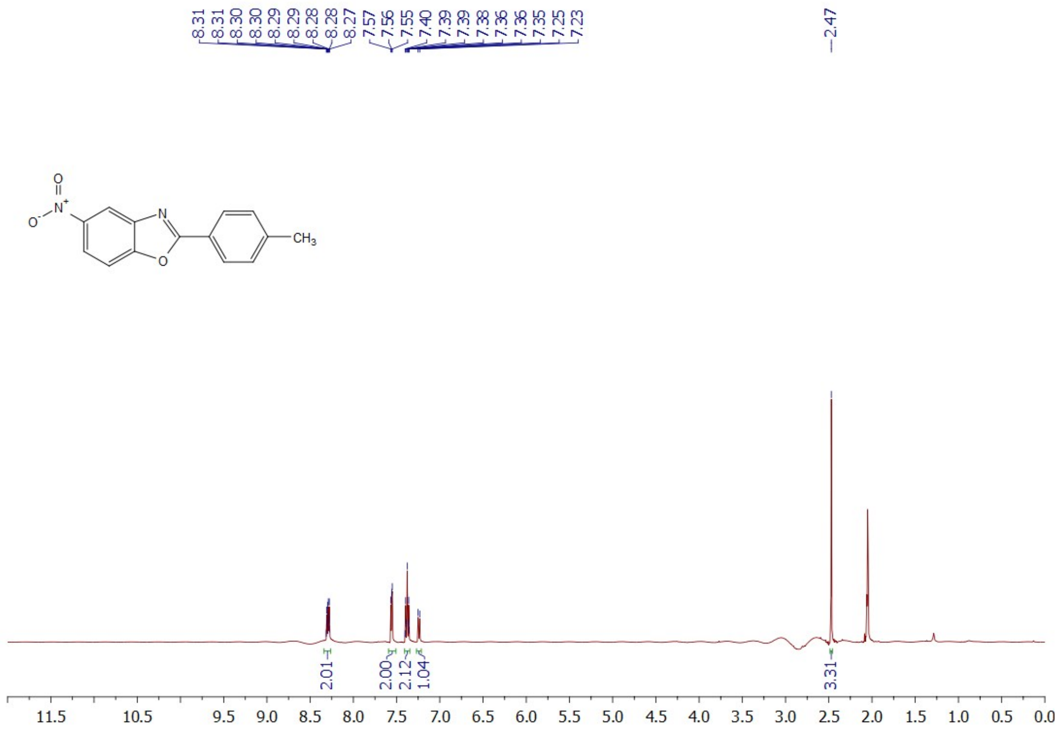

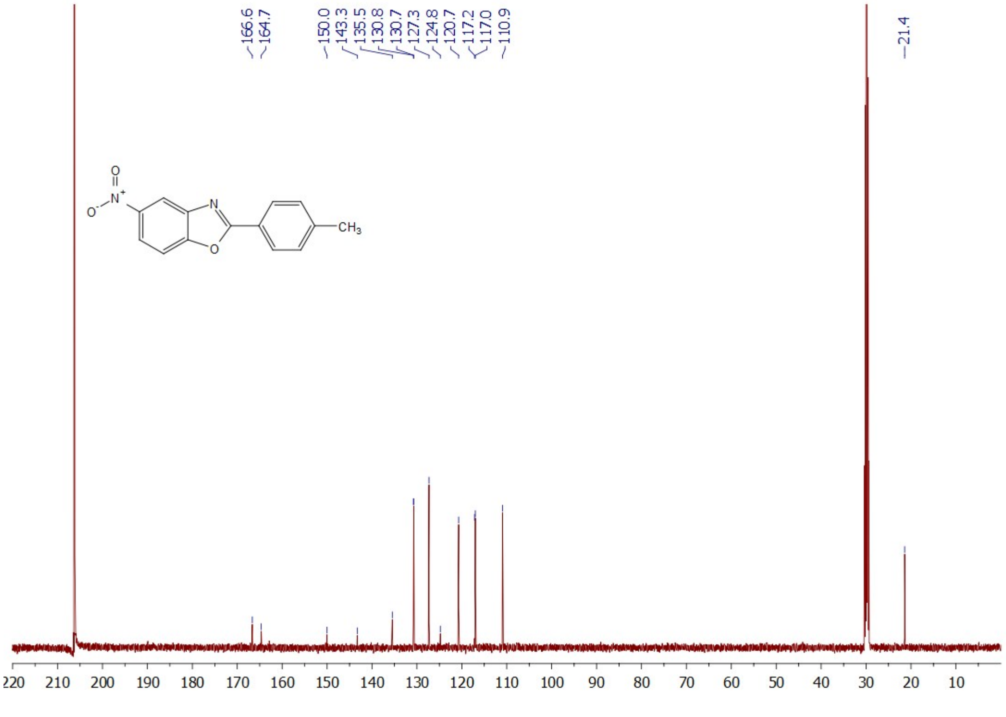

Figure S33. ^{1}H and ^{13}C NMR spectra of 5-nitro-2-(4-methylphenyl)benzoxazole

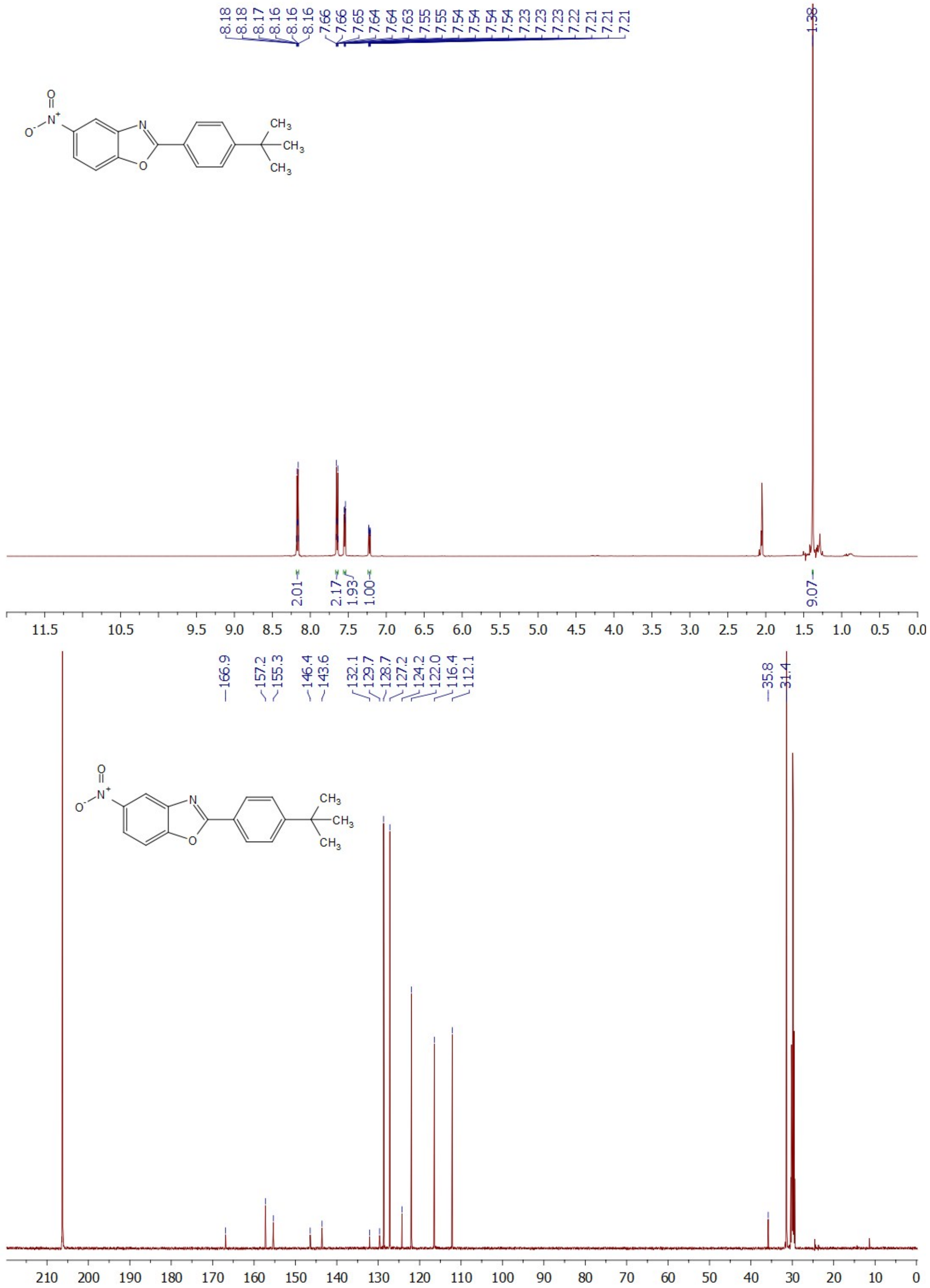

Figure S34. ^{1}H and ^{13}C NMR spectra of 5-nitro-2-(4-*tert*-butylphenyl)benzoxazole

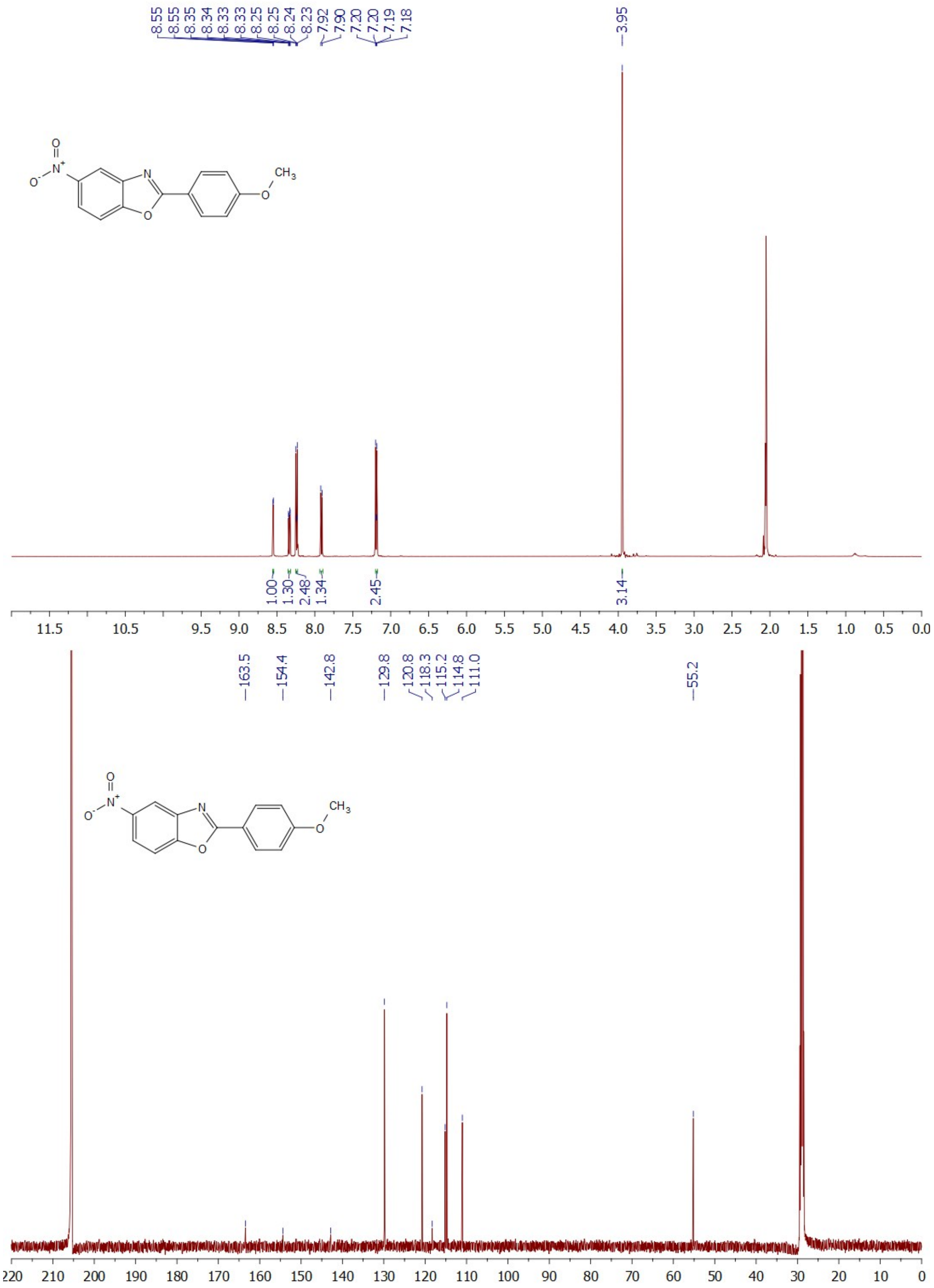

Figure S35. ^{1}H and ^{13}C NMR spectra of 5-nitro-2-(4-methoxyphenyl)benzoxazole

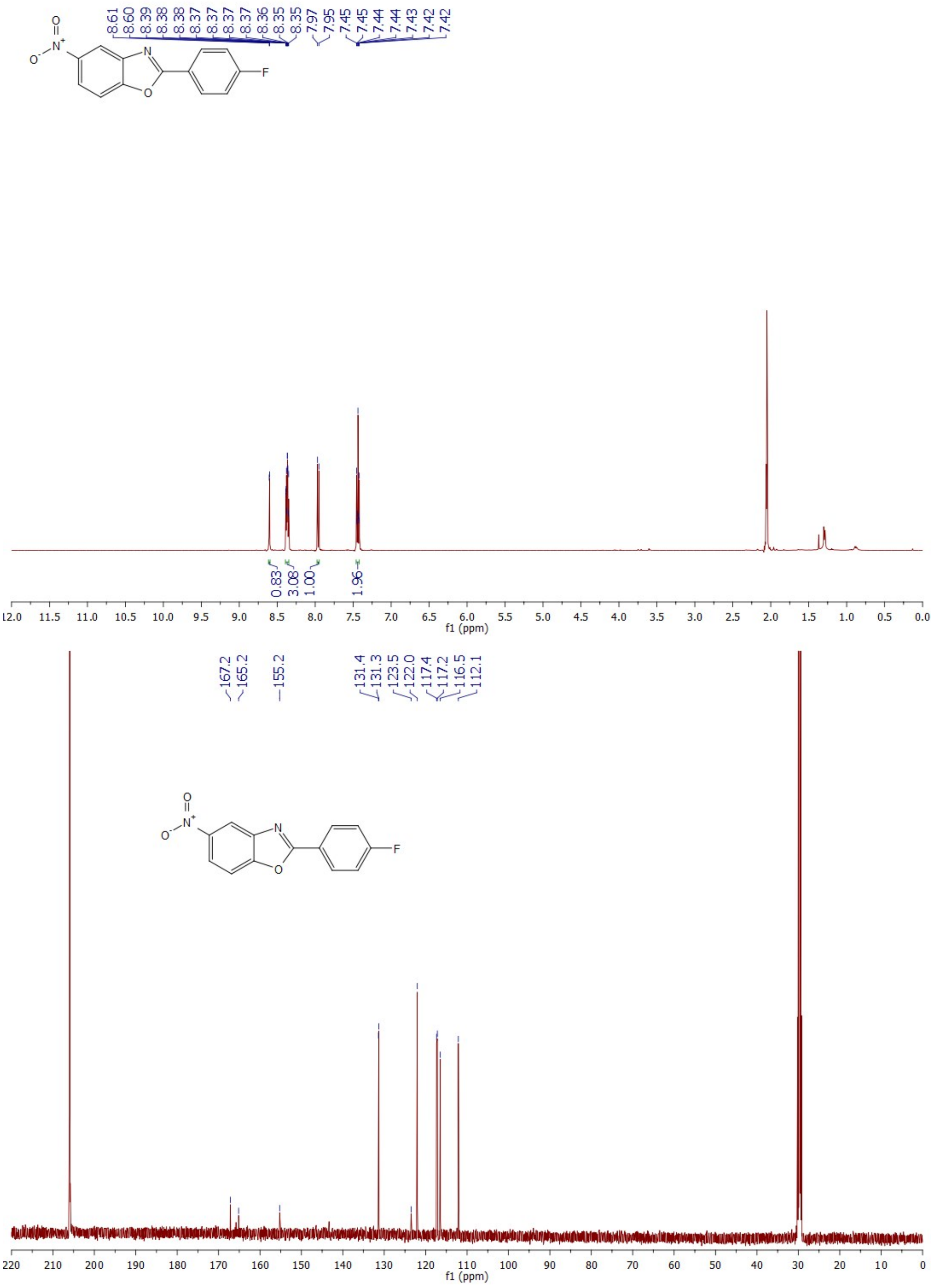

Figure S36. ^{1}H and ^{13}C NMR spectra of 5-nitro-2-(4-fluorophenyl)benzoxazole

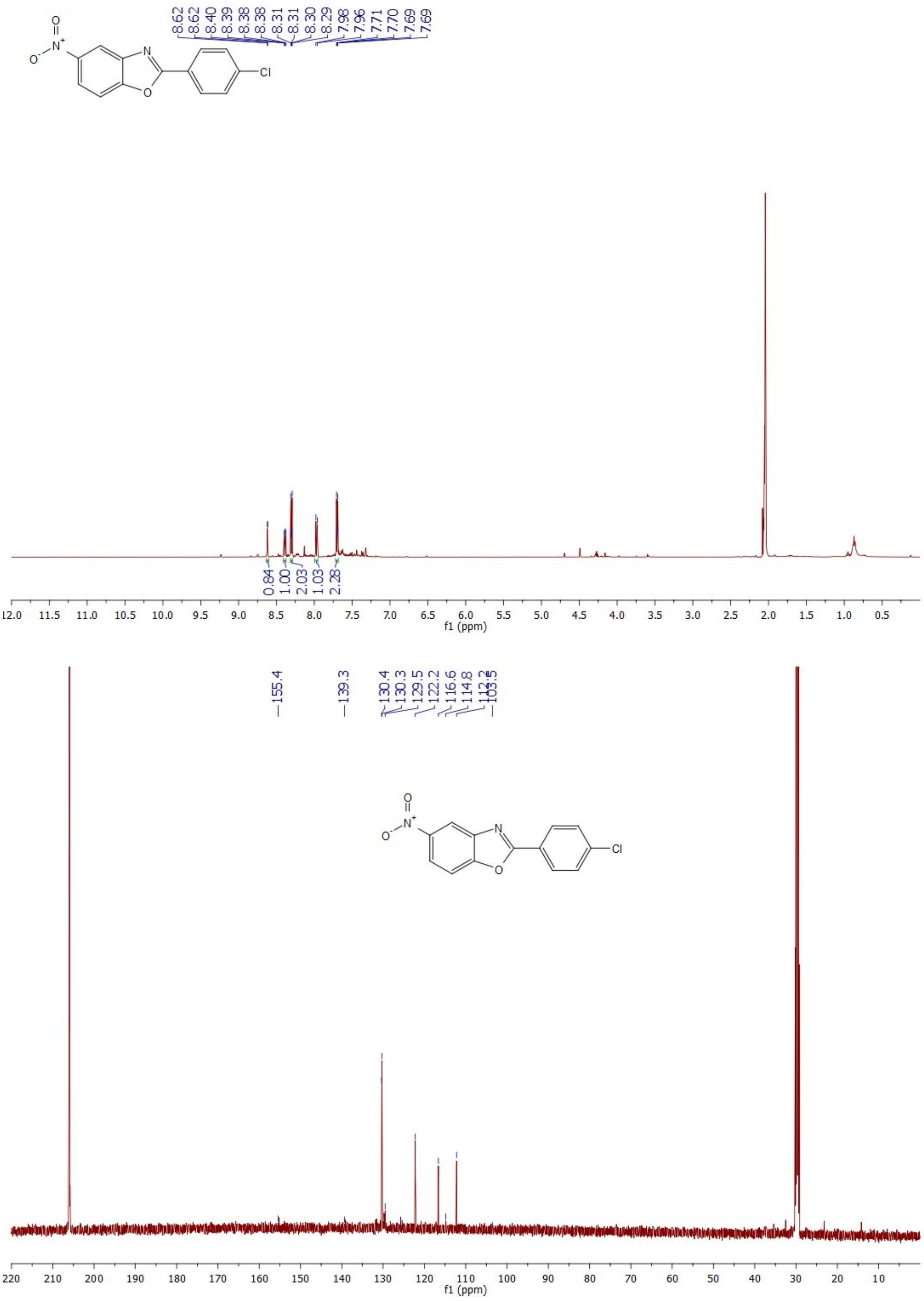

Figure S37. ^{1}H and ^{13}C NMR spectra of 5-nitro-2-(4-chlorophenyl)benzoxazole

Characterization of 2-arylbezothiazoles

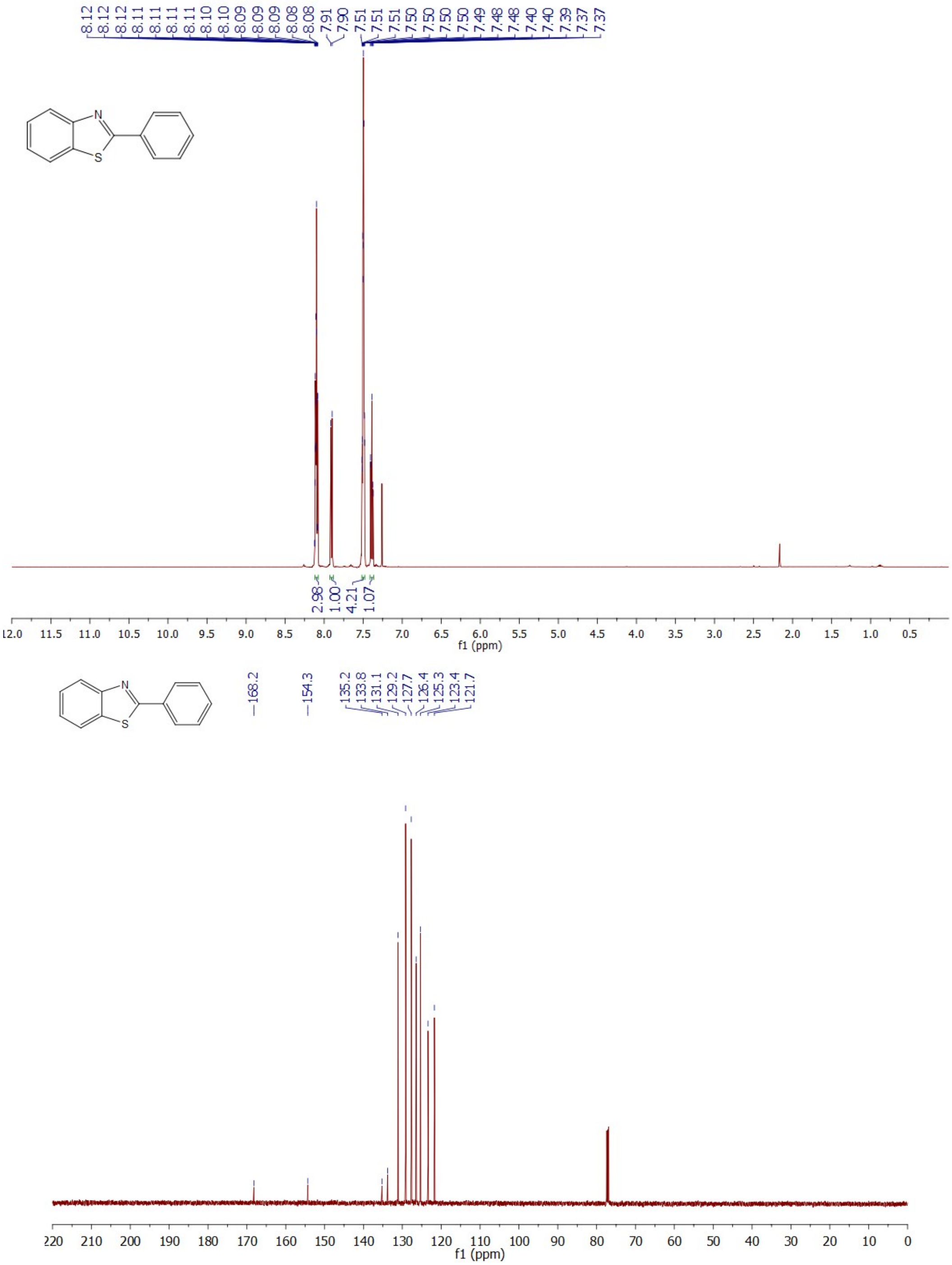

Figure S38. ^{1}H and ^{13}C NMR spectra of 2-phenylbenzothiazole

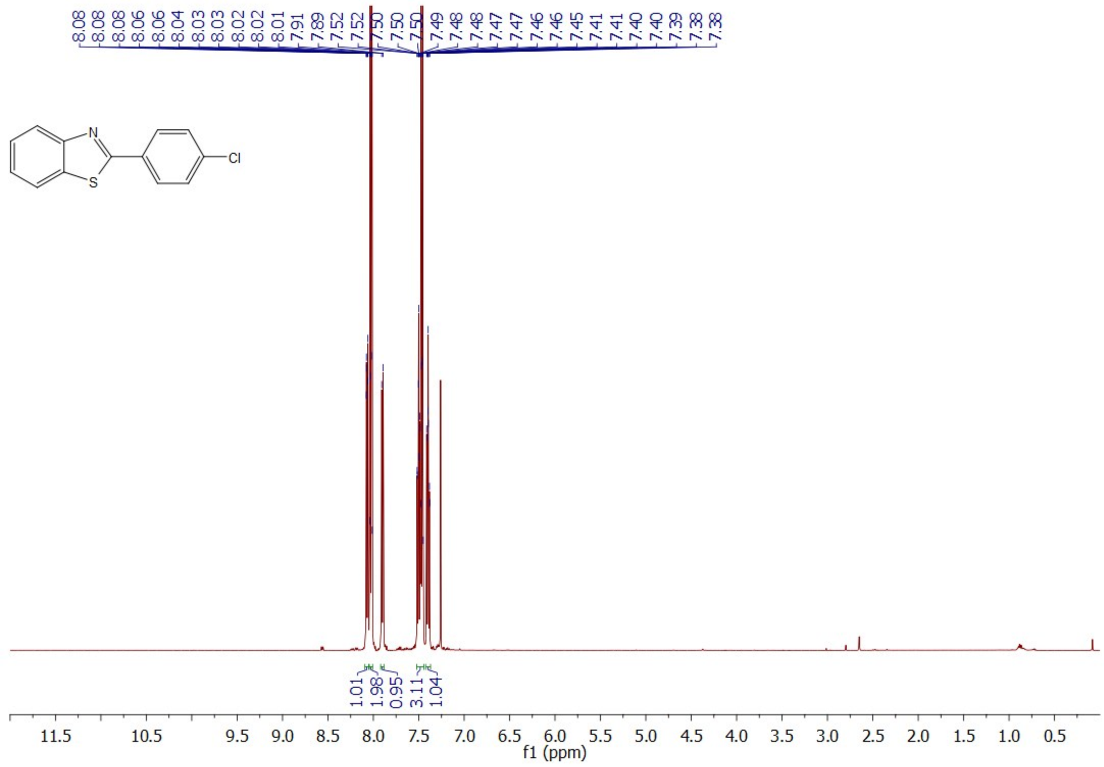

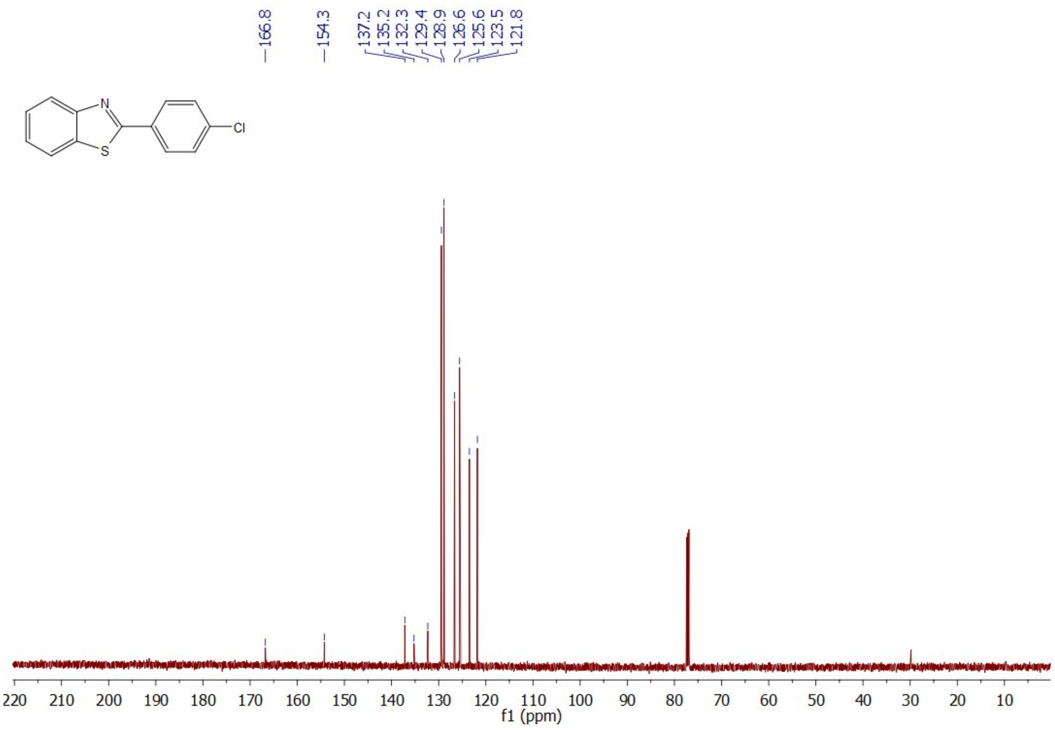

Figure S39. ^{1}H and ^{13}C NMR spectra of 2-(4-chlorophenyl)benzothiazole

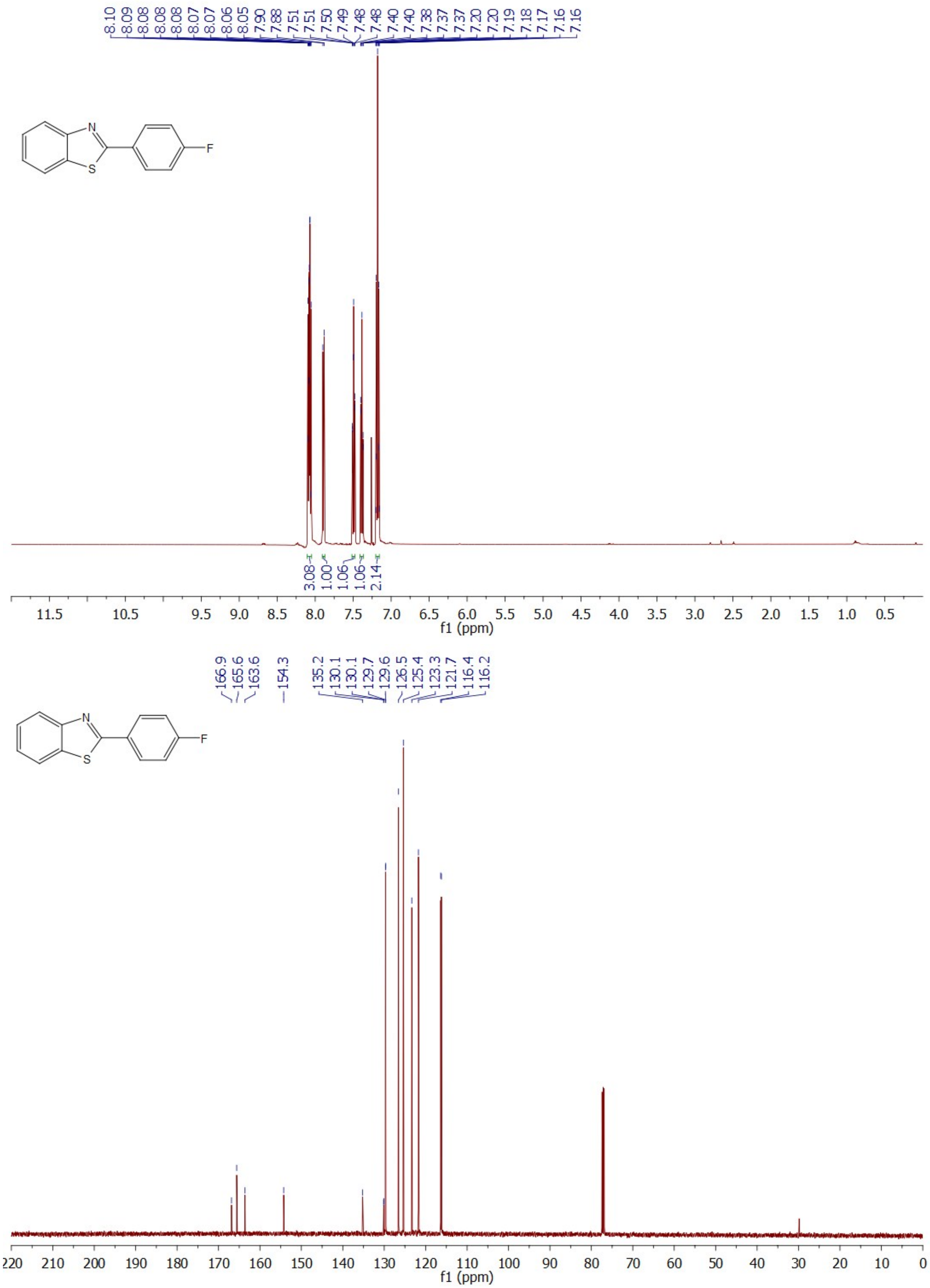

Figure S40. ^{1}H and ^{13}C NMR spectra of 2-(4-fluorophenyl)benzothiazole

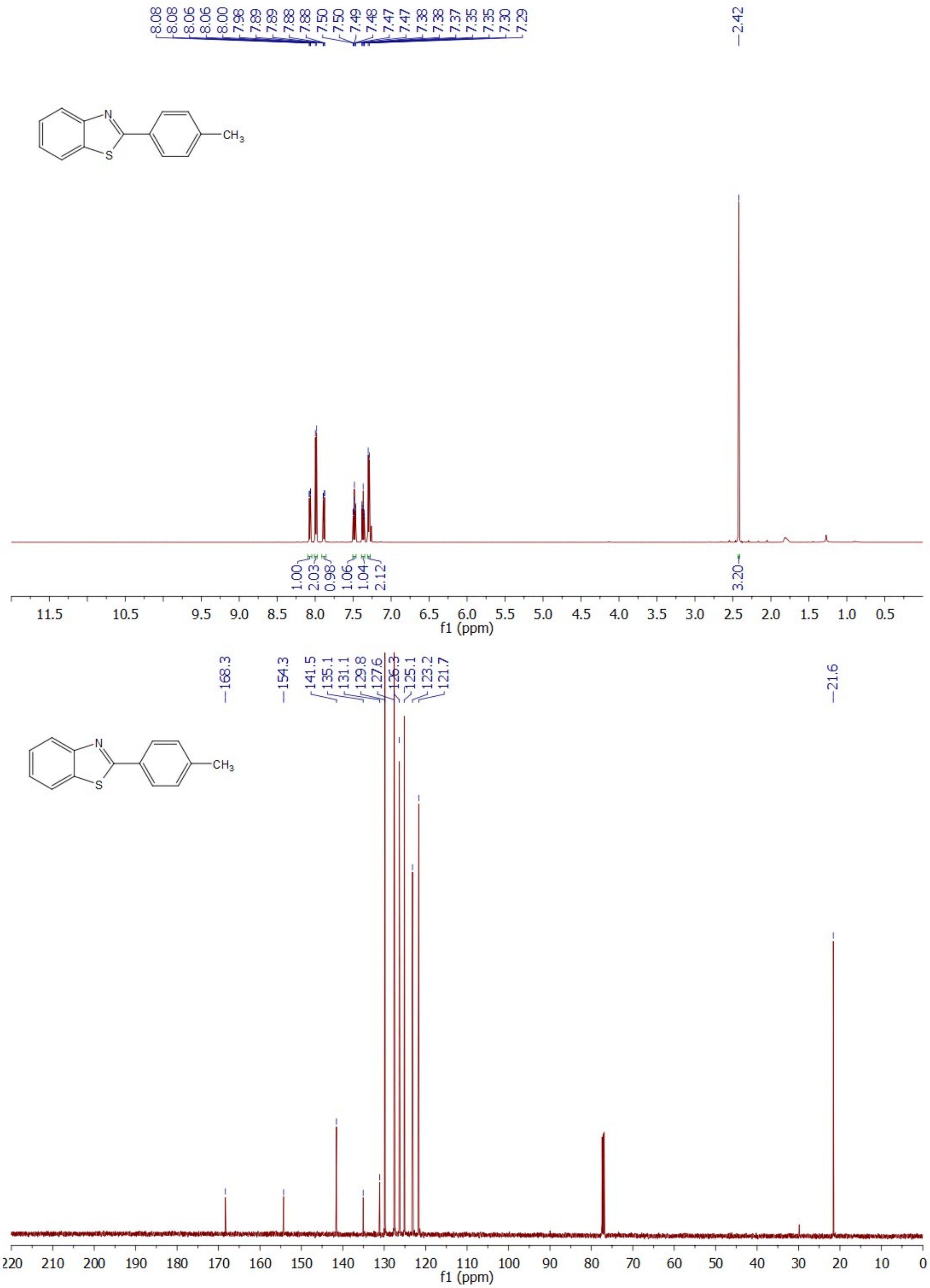

Figure S41. ^{1}H and ^{13}C NMR spectra of 2-(4-methylphenyl)benzothiazole

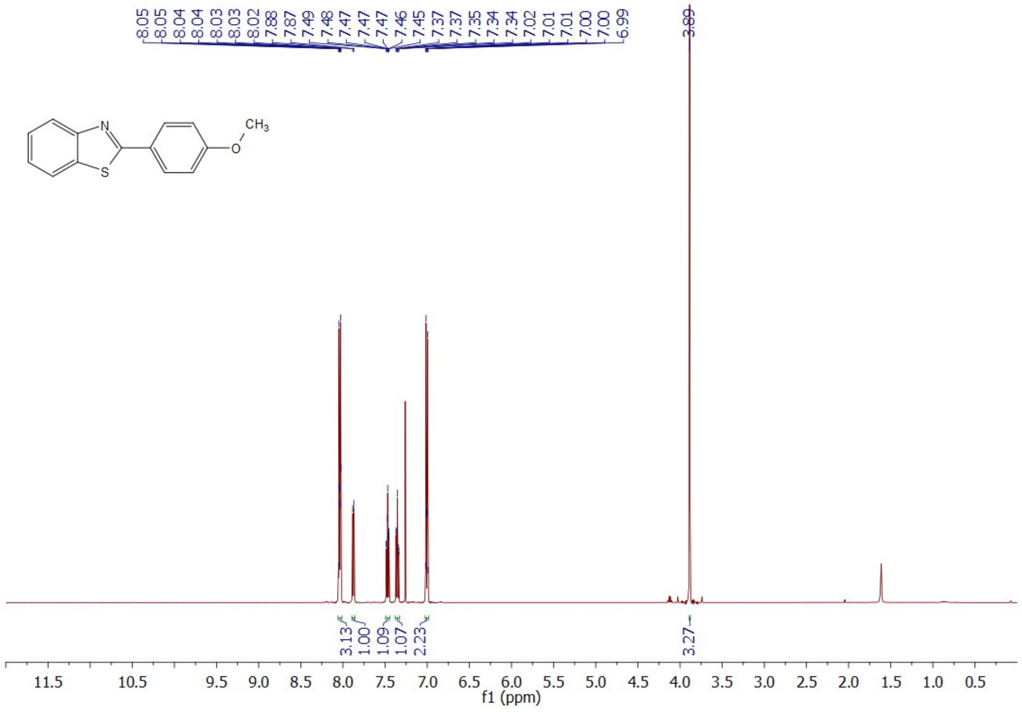

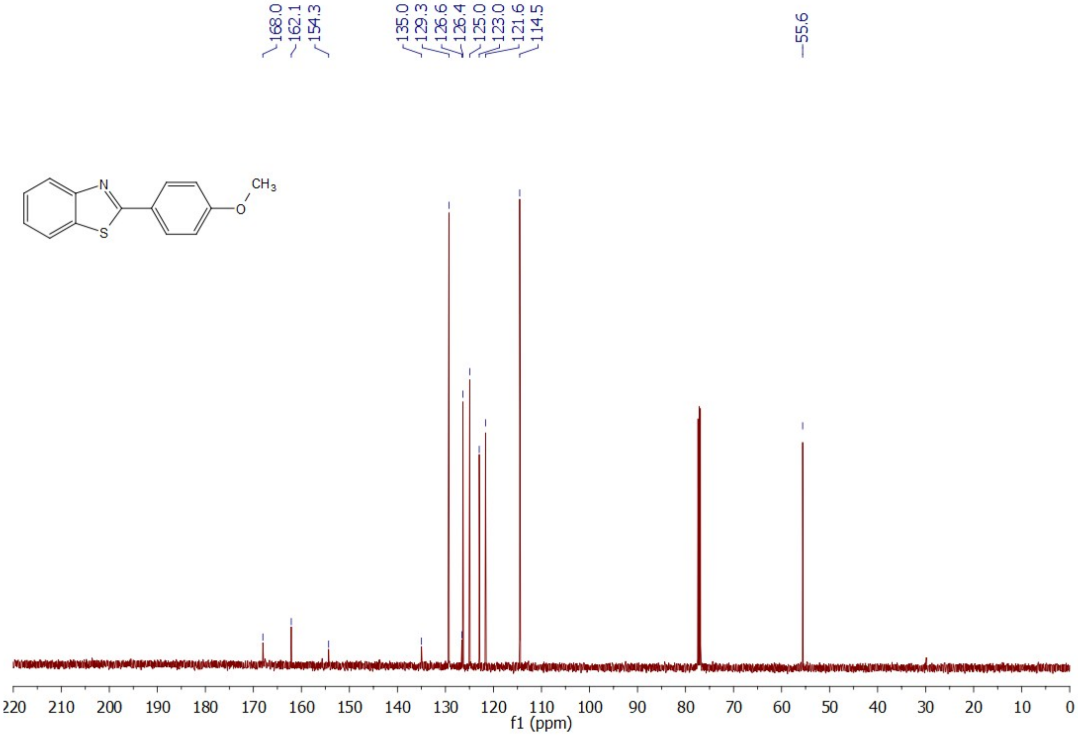

Figure S42. ^{1}H and ^{13}C NMR spectra of 2-(4-methoxyphenyl)benzothiazole

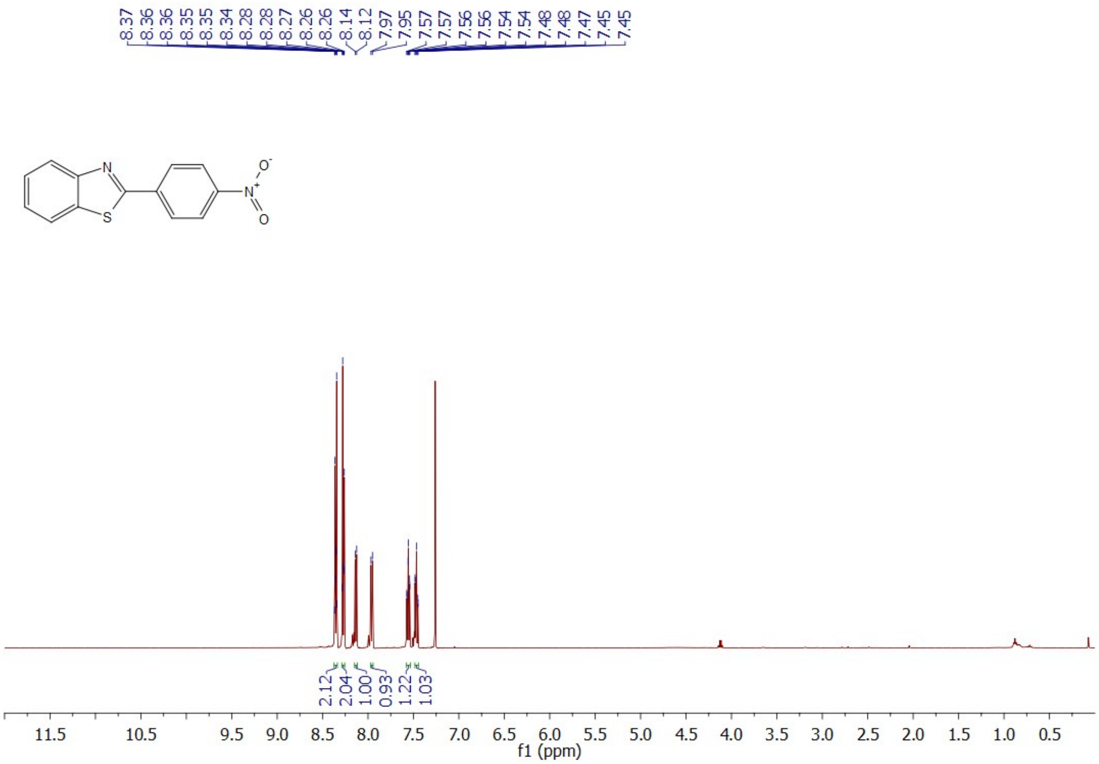

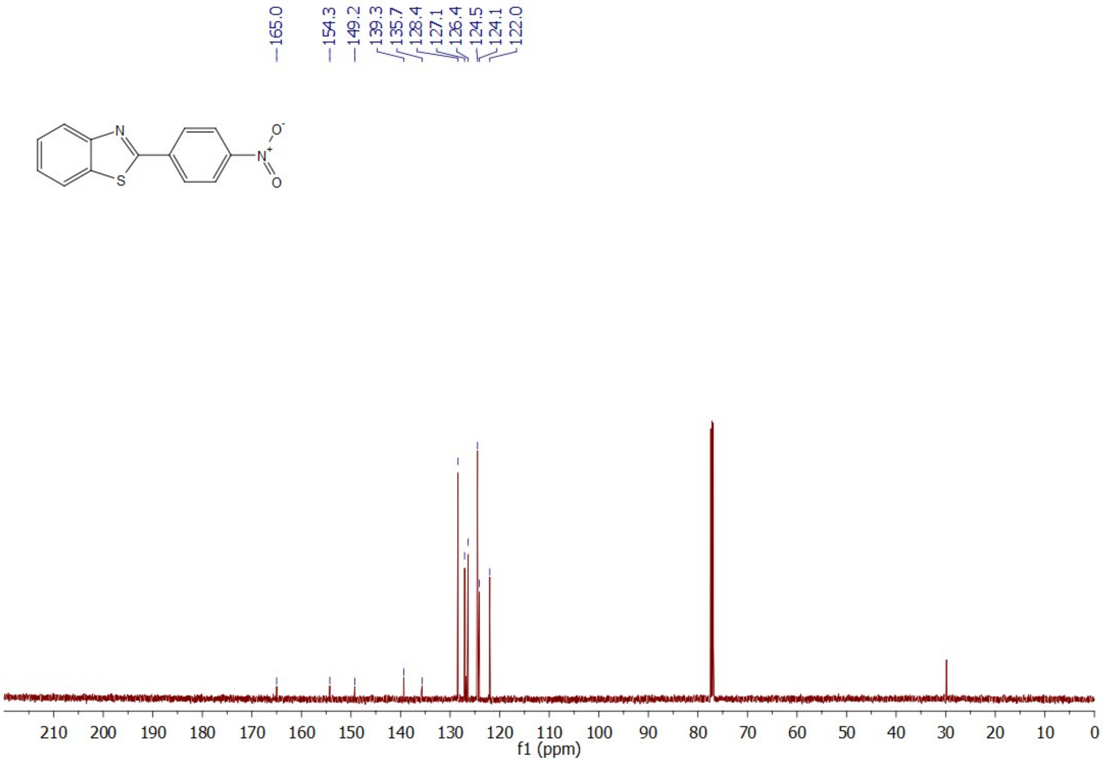

Figure S43. ^{1}H and ^{13}C NMR spectra of 2-(4-nitrophenyl)benzothiazole

Characterization of 2-arylbezimidazoles

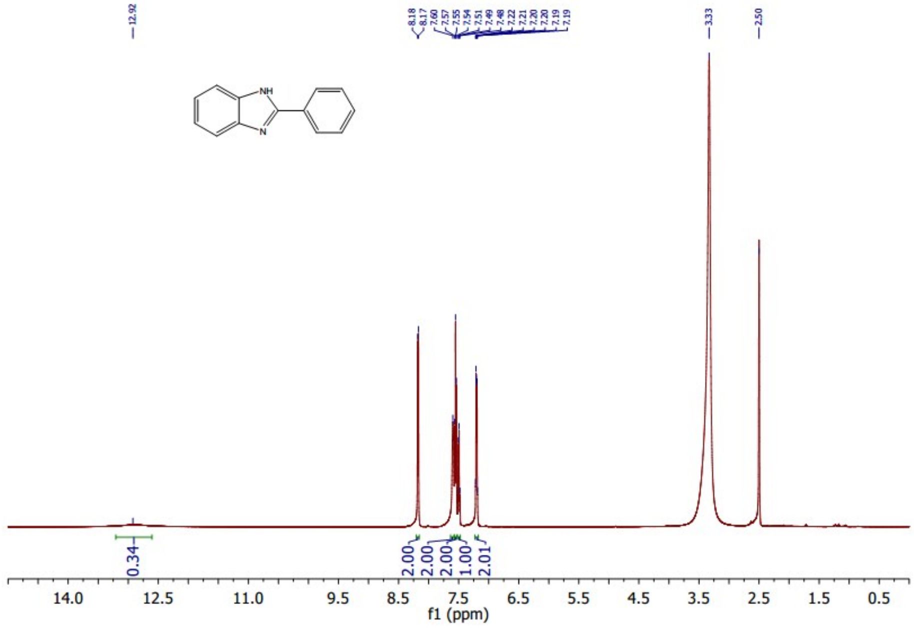

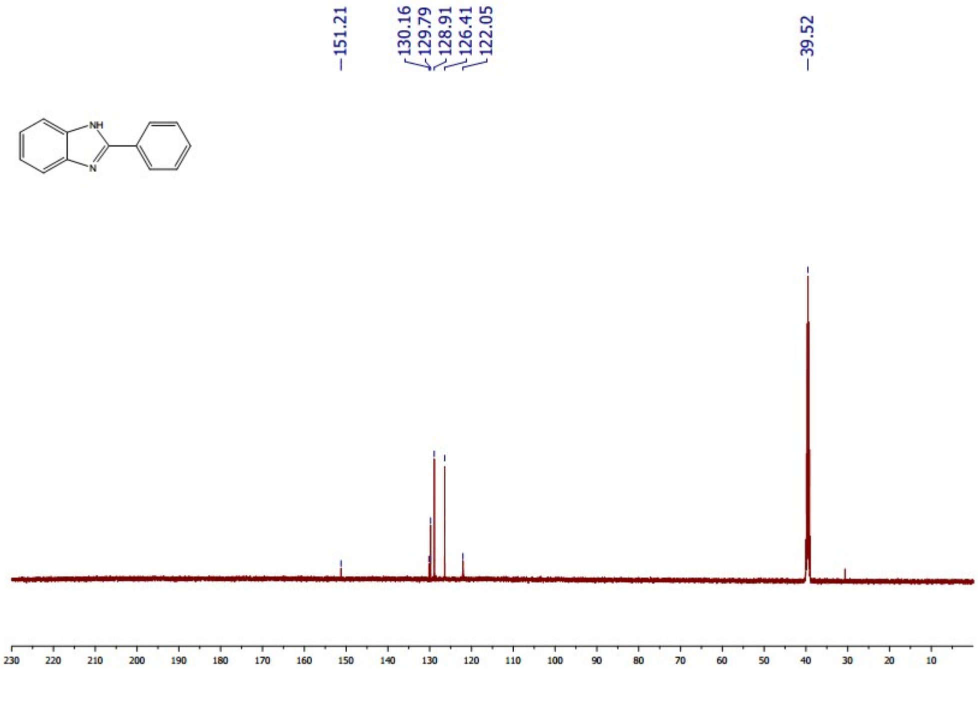

Figure S44. ^{1}H and ^{13}C NMR spectra of 2-phenylbenzimidazole.

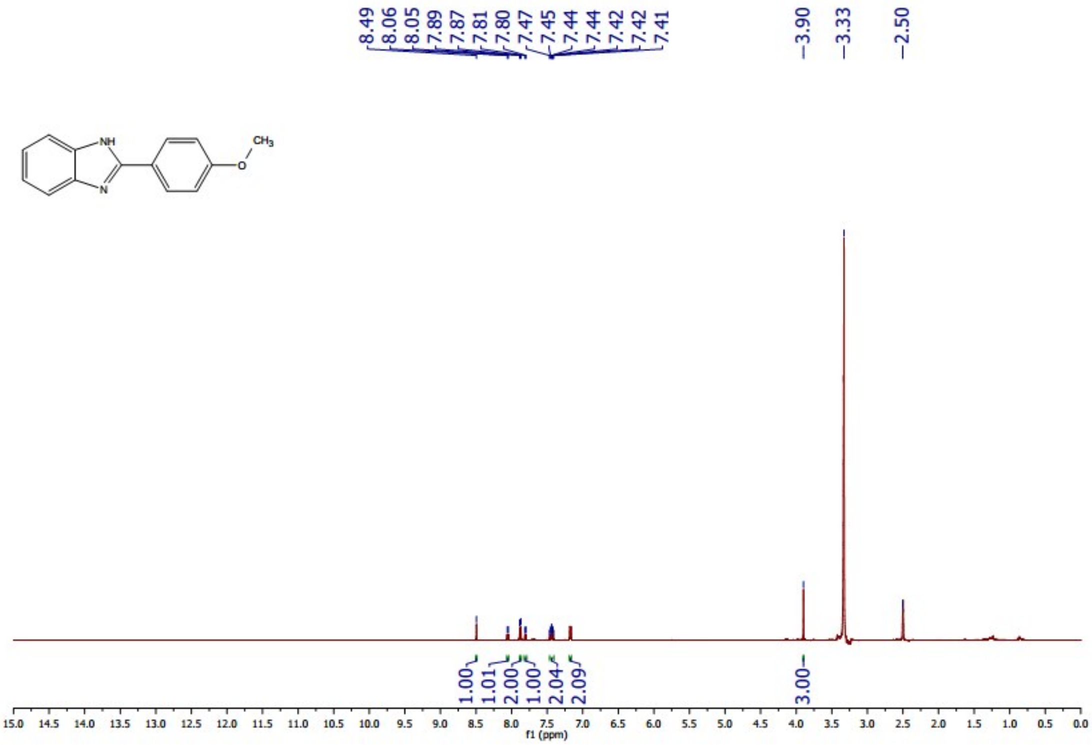

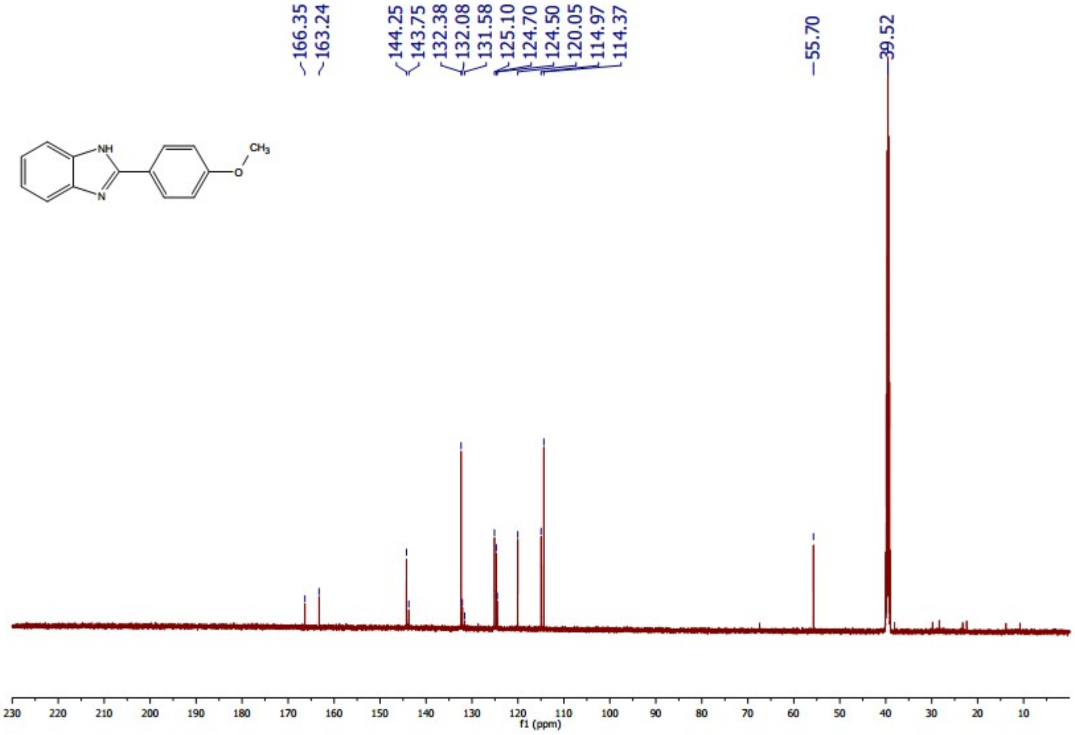

Figure S45. ^{1}H and ^{13}C NMR spectra of 2-(4-methoxyphenyl)benzimidazole.

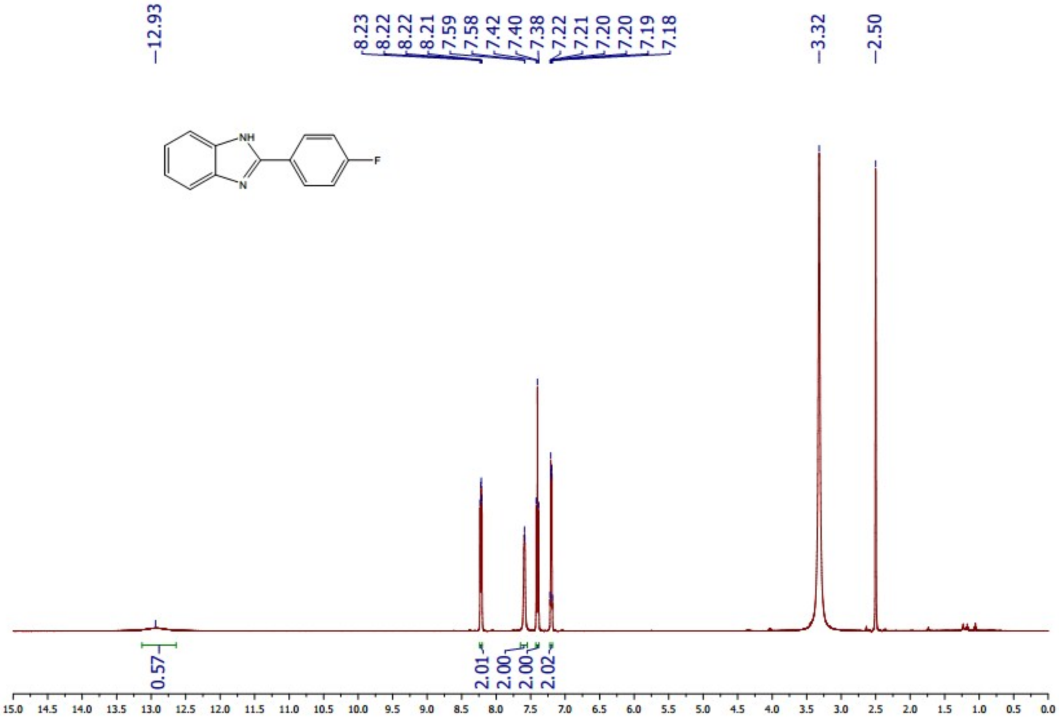

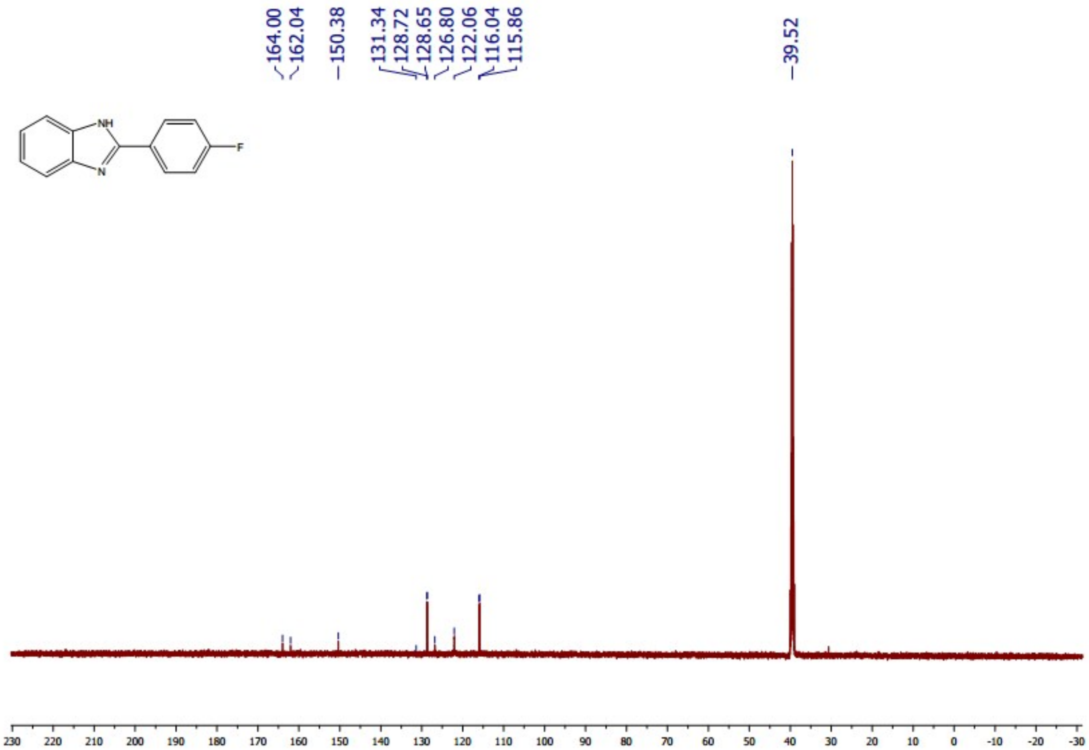

Figure S46. ^{1}H and ^{13}C NMR spectra of 2-(4-fluorophenyl)benzimidazole.

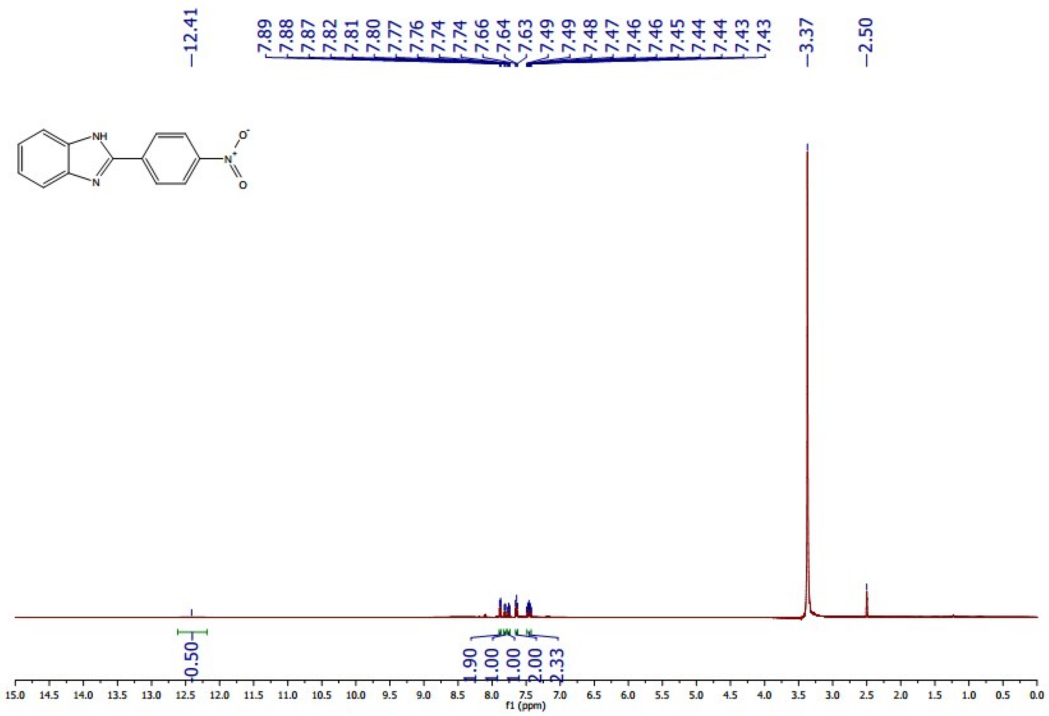

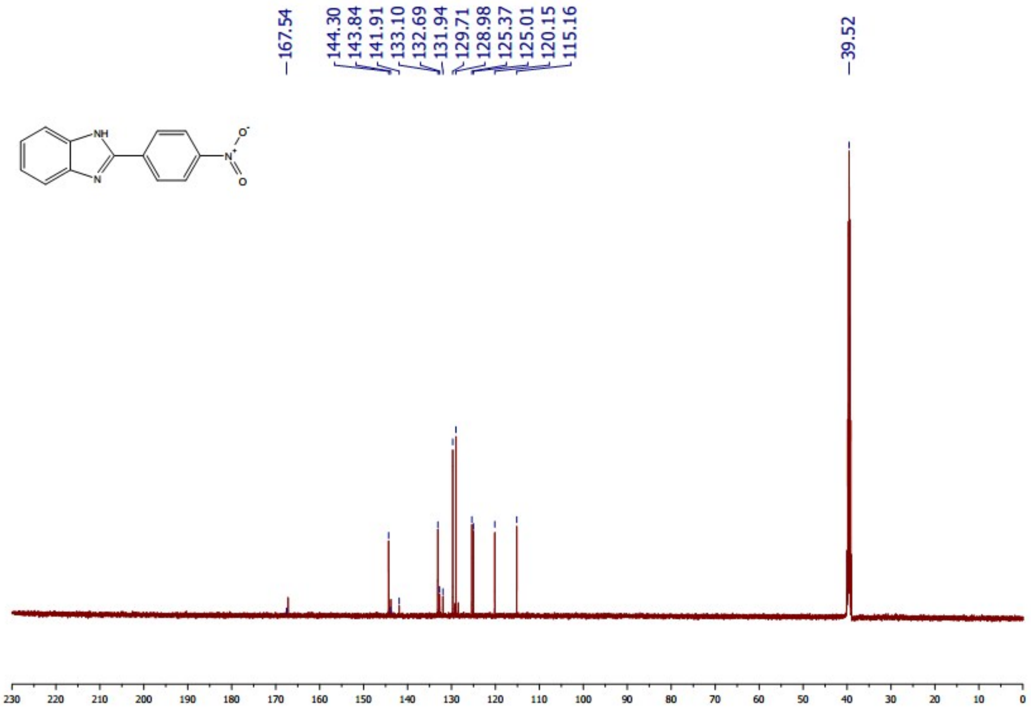

Figure S47. ^{1}H and ^{13}C NMR spectra of 2-(4-nitrophenyl)benzimidazole.

Section S5. References

1. Cole, A. C.; Jensen, J. L.; Ntai, I.; Tran, K. L. T.; Weaver, K. J.; Forbes, D. C.; Davis, J. H. *J. Am. Chem. Soc.* **2002,** *124*, 5962-5963.
2. Teo, Y. C.; Riduan, S. N.; Zhang, Y. *Green Chem.* **2013,** *15*, 2365-2368.
3. Tang, L.; Guo, X.; Yang, Y.; Zha, Z.; Wang, Z. *Chem. Commun. (Camb)* **2014,** *50*, 6145-6148.
4. Patil, M. R.; Bhanushali, J. T.; Nagaraja, B. M.; Keri, R. S. *C. R. Chimie* **2017**.
5. Azizian, J.; Torabi, P.; Noei, J. *Tetrahedron Lett.* **2016,** *57*, 185-188.
6. Zhou, Q.; Zhang, J. F.; Cao, H.; Zhong, R.; Hou, X. F. *J. Org. Chem.* **2016,** *81*, 12169-12180.
7. Gupta, R.; Sahu, P. K.; Sahu, P. K.; Srivastava, S. K.; Agarwal, D. D. *Catal. Commun.* **2017,** *92*, 119-123.
8. Wu, A.; Chen, Q.; Liu, W.; You, L.; Fu, Y.; Zhang, H. *Org. Lett.* **2017**.
9. Steinberg, D. F.; Turk, M. C.; Kalyani, D. *Tetrahedron* **2017,** *73*, 2196-2209.
10. Huang, L.; Zhang, W.; Zhang, X.; Yin, L.; Chen, B.; Song, J. *Bioorg. Med. Chem. Lett.* **2015,** *25*, 5299-5305.
11. Yamada, S.; Murakami, K.; Itami, K. *Org. Lett.* **2016,** *18*, 2415-2418.
12. Srivastava, A.; Shukla, G.; Singh, M. S. *Tetrahedron* **2017,** *73*, 879-887.
13. Reyes, H.; Beltran, H. I.; Rivera-Becerril, E. *Tetrahedron Lett.* **2011,** *52*, 308-310.
14. Prakash, O.; Batra, A.; Sharma, V.; Saini, R. K.; Verma, R. S. *J. Indian Chem. Soc.* **2003,** *80*, 1031-1034.
15. Vosooghi, M.; Arshadi, H.; Saeedi, M.; Mahdavi, M.; Jafapour, F.; Shafiee, A.; Foroumadi, A. *J. Fluorine Chem.* **2014,** *161*, 83-86.
16. Yalçın, İ.; Şener, E.; Özden, T.; Özden, S.; Akın, A. *Eur. J. Med. Chem.* **1990,** *25*, 705-708.
17. Gao, Y.; Song, Q.; Cheng, G.; Cui, X. *Org. Biomol. Chem.* **2014,** *12*, 1044-1047.
18. Sharma, H.; Singh, N.; Jang, D. O. *Green Chem.* **2014,** *16*, 4922-4930.
